# High-quality genome sequence of white lupin provides insight into soil exploration and seed quality

**DOI:** 10.1038/s41467-019-14197-9

**Published:** 2020-01-24

**Authors:** Bárbara Hufnagel, André Marques, Alexandre Soriano, Laurence Marquès, Fanchon Divol, Patrick Doumas, Erika Sallet, Davide Mancinotti, Sébastien Carrere, William Marande, Sandrine Arribat, Jean Keller, Cécile Huneau, Thomas Blein, Delphine Aimé, Malika Laguerre, Jemma Taylor, Veit Schubert, Matthew Nelson, Fernando Geu-Flores, Martin Crespi, Karine Gallardo, Pierre-Marc Delaux, Jérôme Salse, Hélène Bergès, Romain Guyot, Jérôme Gouzy, Benjamin Péret

**Affiliations:** 1 BPMP, Univ Montpellier, CNRS, INRAE, SupAgro, Montpellier, France; 2 LIPM, Université de Toulouse, INRAE, CNRS, Castanet-Tolosan, France; 3 University of Copenhagen, Frederiksberg C, Denmark; 4 CNRGV, INRAE, Toulouse, France; 5 Laboratoire de Recherche en Sciences Végétales (LRSV), Université de Toulouse, CNRS, Castanet Tolosan, France; 6 INRAE GDEC, Clermont-Ferrand, France; 7 Institute of Plant Sciences Paris-Saclay, Gif-sur-Yvette, France; 8 INRAE Agroécologie, Dijon, France; 9 Royal Botanic Gardens, Kew, UK; 10 IPK, Gatersleben, Germany; 11 CSIRO, Perth, Australia; 12 IRD, Montpellier, France INRAE / 13 Department of Electronics and Automatization, Universidad Autónoma de Manizales, Manizales, Colombia; 13 Present Address: MPIPZ, Cologne, Germany

**Keywords:** Agricultural genetics, Plant domestication, Plant genetics

## Abstract

White lupin (*Lupinus albus L*.) is an annual crop cultivated for its protein-rich seeds. It is adapted to poor soils due to the production of cluster roots, which are made of dozens of determinate lateral roots that drastically improve soil exploration and nutrient acquisition (mostly phosphate). Using long-read sequencing technologies, we provide a high-quality genome sequence of a cultivated accession of white lupin (2n = 50, 451 Mb), as well as de novo assemblies of a landrace and a wild relative. We describe a modern accession displaying increased soil exploration capacity through early establishment of lateral and cluster roots. We also show how seed quality may have been impacted by domestication in term of protein profiles and alkaloid content. The availability of a high-quality genome assembly together with companion genomic and transcriptomic resources will enable the development of modern breeding strategies to increase and stabilize white lupin yield.

## Abstract (web summary)

White lupin is an annual crop cultivated for protein rich seeds and can produce cluster roots for efficient phosphate acquisition. Here, the authors generate high quality genome assemblies of a cultivated accession, a landrace, and a wild relative and provides insight into soil exploration and seed quality.

## Introduction

Lupins are commonly known as beautiful ornamental plants, bearing numerous colorful flowers. These plants belong to the *Lupinus* genus that is richly diverse with more than 300 species^1,2^. They are grouped into Old World lupins (Mediterranean) and New World lupins (American) and display a remarkable array of ecological habitats, justifying their interest as a case study for genome evolution, adaptation and speciation^2,3^. Among them, white lupin (*Lupinus albus L*.) is a pulse that originates from the Mediterranean region, its center of origin is believed to be Greece, Western Turkey and southern Balkans where wild ‘*graecus*’ types still persist^4^. This crop is recognized as a traditional food due to its very high protein content (between 30 and 40% of the whole seed)^5^.

Cultivation of white lupin (WL) started around 4000 years ago but modern breeding efforts have been very limited and focused on a few major traits such as permeable seeds, early flowering, non-shattering pods, and low alkaloid seed content^6^. WL cultivation has the potential to solve several issues related to the future of European protein supply due to its high quality seeds (very high levels of proteins, high levels of tocopherols, lowest glycemic index of consumed grains, high dietary fiber content, gluten-free, low oil, and minimal starch)^5,7–9^. It is also a crop with low need for phosphate fertilizers due to its highly adapted root system^10^ and no need of nitrogen input as a legume.

White Lupin is one of the few crops that can produce spectacular structures called cluster roots, harboring a specific physiology dedicated towards efficient Pi acquisition^11^. Despite being an essential micronutrient, inorganic phosphate is poorly available in the soil and plants have developed various strategies to improve Pi remobilization and acquisition^12,13^. Most terrestrial plants form mycorrhizal symbiosis to improve soil exploration but WL has lost the ability to form such associations. Instead, by producing cluster roots, WL can take up almost 5 times more Pi per root length unit than soybean, a mycorrhizal legume that does not form cluster roots^14^, suggesting a strong potential for crop improvement towards better nutrient acquisition efficiency^11^.

In this study, we present a high-quality genome sequence of a modern accession of white lupin (2n = 50, 451 Mb), as well as de novo assemblies of a landrace and a wild accession. This quality reference sequence allows us to perform in-depth analysis of repetitive elements, to analyze genomic variations across 15 accessions, and to retrace the paleohistory of legumes. We then provide a comparison of soil exploration capacity between a cultivated and a wild accession, highlighting the early establishment of lateral and cluster roots in the modern cultivar. We also provide information regarding seed quality, demonstrating that modern accessions accumulate specific types of conglutins. Finally, we provide a list of candidate genes present in the *pauper* locus, which is a common QTL controlling the accumulation of toxic alkaloids in WL seeds.

## Results

### Genome assembly and annotation

We generated 164x sequencing coverage of the genome of *Lupinus albus* cv. AMIGA using 30 single-molecule real-time (SMRT) cells on PacBio Sequel platform. The production of 94 Gb of very long reads along with a depth of 208× (119 Gb) of Illumina 150 bp paired-end sequences for the assembly polishing and with the addition of Bionano optical map technology allowed a genome assembly of 451 Mb. The contig sequences obtained by a meta assembly strategy based on CANU^15^ and FALCON^16^ were scaffolded in a first step using a Bionano optical map and in a second step using a high density genetic map^17^. The chromosome-level assembly (termed Lalb, Table 1, Supplementary Fig. 1) covers the 25 nuclear chromosomes along with mitochondrial and chloroplastic genomes, leaving only 64 unanchored contigs (8.8 Mb - 2% of the assembly). The maximum number of sequence gaps is four (on chromosomes 10 and 11) and ten chromosomes contain only a single sequence gap, illustrating the high and homogenous contiguity across chromosomes (Supplementary Note 1, Supplementary Data 1, Supplementary Tables 1–3).

**Table 1:** Statistics of the white lupin genome and gene models prediction.

	Number	Size
*Assembly feature*		
Assembled sequences	89	450.972 Mb
N50	12	17.35 Mb
N90	23	14.55 Mb
GC content (%)	33.71	
*Genome annotation*		
TE proportion (%)	60	
Annotated protein-coding genes	38258	
Annotated non-protein coding genes	3129	
Complete BUSCOs	1331 (97.7%)	
Fragmented BUSCOs	3 (0.2%)	
Missing BUSCOs	29 (2.1%)	

We generated RNA-seq data from ten different organs, widely covering gene expression in WL (entire root system in +Pi and –Pi conditions, lateral roots, primary roots, cluster roots, nodulated root system, leaves, flowers, pods, and seeds). The assembled reads were mapped using EuGene-EP pipeline^18^ and protein and non-protein coding gene models were predicted. Three protein databases (Swiss-Prot, a plant subset of Uniprot proteins and the proteome of *Medicago truncatula*) were aligned to contribute to translated regions detection. Genome annotation identified 38,258 protein-coding genes and 3129 non-protein-coding genes (Table 1 and Supplementary Note 2). Evidence of transcription was found for 92% of the annotated genes. Quality of the annotation was evaluated with a Benchmarking of Universal Single-Copy Orthologs (BUSCO^19^) analysis, yielding a completeness score of 97.7%. The WL Genome portal (www.whitelupin.fr) provides a genome browser and several other user-friendly tools for molecular analysis.

### Repetitive elements and structure of centromeric regions

**Fig. 1:**
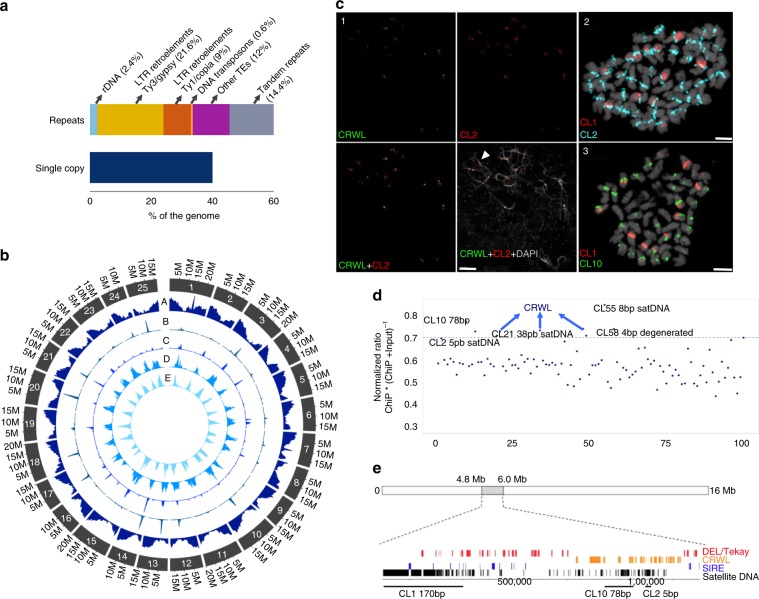
Repeated elements abundance in white lupin genome. **a** Proportion of single copy and repeated sequences for the different classes of repeats. **b** Density distribution along the chromosomes of the repetitive classes. A. genes, B. CRM, C. satDNA, D. Tekay, and E. SIRE. Density is represented in 0.5 Mb bins. **c** FISH mapping of the main repeats by super-resolution microscopy (3D-SIM). (1) Co-localization of CRWL and CL2 satDNA on centromeric regions of meiotic (pachytene) chromosomes. (2–3) Distribution of the most abundant satellite DNAs CL1, CL2, and CL10 in somatic metaphase chromosomes. Bars are 10 µm (1) and 2 µm (2 and 3). **d** LalbCENH3-ChIPseq reads mapped against the first 100 RepeatExplorer clusters of the WL genome. The main centromeric sequences found in LalbCENH3-ChIPseq are highlighted. **e** Typical centromere composition of a WL chromosome, chromosome 14. Source data underlying Fig. 1d are provided as a Source Data file.

De novo identification of repeated elements revealed a highly repetitive genome (60%), with over 75% repeats matching known transposable elements (TEs, Fig. 1a, Supplementary Note 3, Supplementary Tables 4). Chromosomal scale genome-wide annotation of repetitive sequences revealed the in silico annotation of the main classes of repeats (Fig. 1b). TEs were most commonly long terminal repeats (LTRs) retrotransposons (34%), with remarkable accumulation of Ty3/gypsy Tekay, CRM chromoviruses and Ty1/copia SIRE towards the central regions of chromosome assemblies along presumed (peri)centromeric regions (Fig. 1b). Class II TEs accounted for ca. 0.8% of the genome (Supplementary Note 3) and is in accordance to the lower abundance of this class of repeats in other legume species^20,21^. A high amount of satellite DNA (satDNA) sequences was found, comprising ~15% of the genome (Supplementary Note 3, Supplementary Table 5). A narrow peak for the distribution of CRM (Centromeric Retrotransposon of Maize) clade retroelements was observed in all assembled chromosomes, we therefore presumed that the observed peak defines the centromeric regions of WL chromosomes. Thus, we refer to this element as CRWL for Centromeric Retrotransposon of While Lupin. Remarkably, a high association of CRWL and satellite DNA peaks was observed, suggesting a more specific distribution of these repeats compared to other TEs (Fig. 1b, c) whereas a more diverse distribution of repetitive elements towards the peri-centromeric regions of WL chromosomes was observed (Supplementary Fig. 2). To further characterize the repeat portion of WL genome we performed in situ hybridization of the most abundant repeat clusters identified by the RepeatExplorer analysis (Fig. 1c). As expected, CRWL FISH signals were observed as narrow distributed signals at the centromeric regions of most WL chromosomes (Fig. 1c, Supplementary Fig. 2), giving an indication of functional centromeres positioning.

Raising an anti-LalbCENH3 specific antibody, we mapped functional centromeres using immunostaining (Supplementary Fig. 3) and performed LalbCENH3-ChIPseq confirming the association of CRWL main clusters (Fig. 1d) with functional centromeres. Analysis of ChIPseq reads demonstrated that CRWL elements (CL13, CL20, CL34, CL48, and CL49) are among the clusters that showed the highest levels of association in the immunoprecipitated fraction (Fig. 1d). Although CRWL is highly abundant on centromeric regions of WL chromosomes (Fig. 1b, c), detailed analysis of ChIPseq data revealed that CENH3-containing chromatin is also associated with at least four families of centromeric tandem repeats: CL2-5bp, CL10-78bp, CL21-38bp and CL55-8bp (Fig. 1c, d). Super-resolution microscopy of pachytene and somatic chromosomes confirmed a centromere-specific localization for CRWL, CL2, CL10, CL21, and CL55 repeats, while CL1 repeat localizes aside core centromeres (Fig. 1c, Supplementary Fig. 2 arrowheads). The total amount of cenDNA represents about 11% (49.55 Mb) of the genome. In contrast, the most abundant satDNA CL1-170bp did not show significant enrichment with the immunoprecipitated DNA, suggesting that this element is excluded from functional centromeres. A typical (peri)centromeric region of a WL chromosome contains the most abundant CL1-170bp repeats representing 18% of the region. These sequences are organized in blocks separated by SIRE retrotransposons. Centromere-associated satellite repeats are present in shorter arrays such as CL2-5bp and CL10-78bp intermingled with CRWL elements (Fig. 1e, Supplementary Fig. 3, Supplementary Table 6). Thus, the functional centromeres of WL are preferentially associated with CRWL and with different families of tandem repeats in a chromosome-specific pattern. These results identify a specific centromeric sequence pattern with a highly diverse structure in WL that strongly differs from known centromeric sequences.

### White lupin diversity and genomic structural variations

**Fig. 2:**
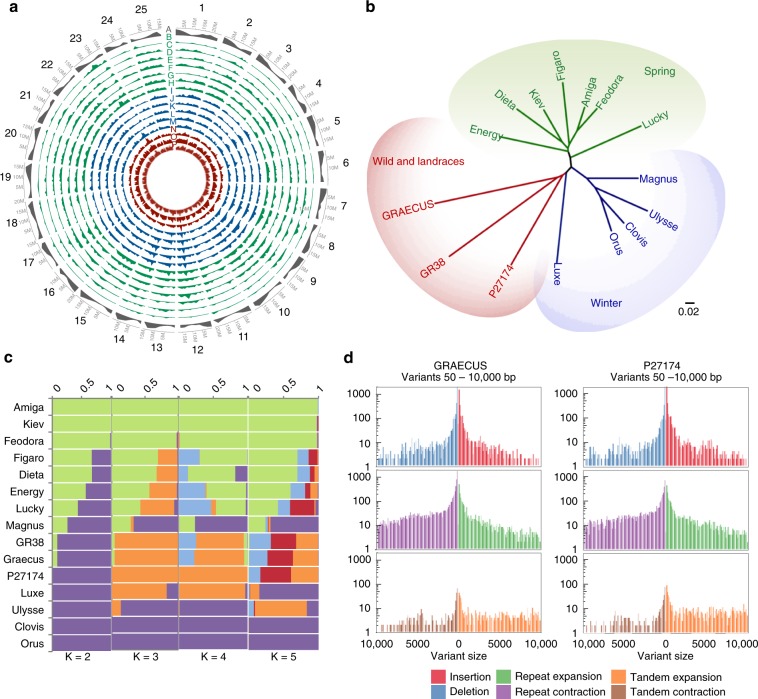
White Lupin diversity and evolution history. **a** SNP density identified by resequencing of 15 accessions of white lupin. In the outer track (A in gray) gene density is shown. Spring accessions are represented in green (B–H), winter accessions are represented in blue (I–M) and wild/landrace accessions are represented in red (N-P). From the outer to inner track: (B) AMIGA, (C) FEODORA, (D) KIEV, (E) DIETA, (F) FIGARO, (G) ENERGY, (H) LUCKY, (I) ORUS, (J) CLOVIS, (K) MAGNUS, (L) ULYSSE, (M) LUXE, (N) P27174, (O) GR38, AND (P) GRAECUS. The SNP density is represented in 1 Mb bins. **b** Neighbor-joining phylogenetic tree of white lupin accessions based on SNPs. The 15 accessions are divided in three clades: winter, spring and wild/landrace. **c** Admixture representation of the 15 accessions with population clustering for *K* = 2–5. Each individual is represented by a horizontal bar and each color represents a subpopulation. The color of each individual accession represents their proportional membership in the different populations. **d** Structural variants (SVs) between *L. albus* cv. AMIGA and the de novo assembly of GRAECUS (left) and P27174 (right). The biggest proportion of variants are the repeated elements. SVs represent 18.08 Mb of the GRAECUS genome and 18.67 Mb of P27174 genome. Source data underlying Fig. 2b are provided as a Source Data file.

To provide a first overview of WL diversity and possible domestication patterns we re-sequenced 14 WL accessions, including 11 modern accessions, 1 landrace and 2 wild relatives (Supplementary Note 4, Supplementary Tables 7 and 8). The accessions presented a total of 2,659,837 SNPs (Fig. 2a, Supplementary Table 9) when compared to the reference genome. Pairwise dissimilarities analyses allowed the identification of three clusters reflecting white lupin recent breeding history: winter accessions (vernalization responsive, slow growth, cold adapted), spring accessions (vernalization unresponsive, fast growth, strong vigor, and reduced life-cycle) and landraces/wild types (Fig. 2b, Supplementary Data 2).

To further verify the clustering observed in the phylogenetic tree, a principal component analysis (PCA) was conducted using the same samples and SNP set. More than half of total genetic variance (58.2%) could be explained by the two first components, which replicates the phylogenetic tree results (Supplementary Fig. 4).

The population structure was explored with the same set of SNPs using STRUCTURE^22^. We tested for a population structure ranging from 2 subpopulations (*K* = 2) up to 5 subpopulations (*K* = 5, Fig. 2c). Additionally, a Evanno’s test^23^ indicated that these 15 WL accessions might be divided into two subpopulations, one formed by the spring accessions and the other with the winter and non-domesticated accessions (Supplementary Fig. 5).

We estimated the level of linkage disequilibrium (LD) using the *r*^2^ parameter between all pairwise SNP comparisons by using these 15 accessions in the 25 chromosomes (Supplementary Fig. 6). We used a subset of 46,783 high-quality genomic-random distributed SNPs. LD decay distance showed apparent variation, indicating that selection pressure in different chromosomal regions varied, probably due to different selection goals in breeding.

We selected the wild accession GRAECUS and an Ethiopian landrace (P27174) to further investigate the possible impact of domestication on WL genome. We sequenced these two genotypes using Nanopore long-read technology, at a depth of 27.6x and 32.4x for GRAECUS and P27174, respectively, and generated de novo assemblies (Supplementary Table 10). Using Assemblytics^24^ (based on whole genomes alignments generated with MUMmer^25^), we identified a high level of structural variations (SVs, Fig. 2d, Supplementary Data 3). This analysis reveals genomic regions that are strongly altered between the modern accession AMIGA and the two accessions that have not undergone a breeding program. P27174 assembly has a total length of 18.67 Mb of structural variations (SVs) affected and the GRAECUS accession was similarly affected by SVs (18.08 MB – Fig. 2d, Supplementary Data 3).

The majority of the SVs in both GRAECUS and P27174 are located in intergenic regions (62 and 53%, respectively). Considering a promoter region of 2 Kb upstream of 5′-UTR, a total of 8166 genes are impacted by SVs in the GRAECUS genome, whereas only 6524 genes are impacted in P27174. A total of 3463 common genes are altered in both accessions and 671 of these genes have common exons impacted (Supplementary Fig. 7). These SVs highlight genomic regions that may help understand major events associated with WL domestication.

### White lupin genome evolution

**Fig. 3:**
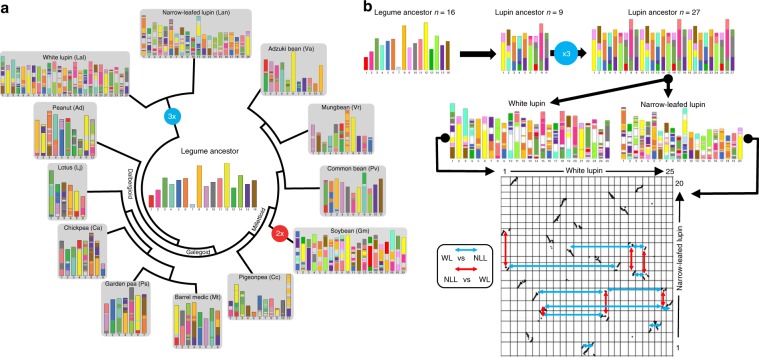
White Lupin genome evolution. **a** Legumes evolutionary history. Evolutionary scenario of the modern legumes (white and narrow-leafed lupin, garden pea, peanut, Lotus, barrel medic, chickpea, pigeonpea, soybean, common bean, mungbean, and adzuki bean) from the reconstructed ancestral legume karyotype (ALK, center). The modern genomes are illustrated with different colors reflecting the origin from the ancestral chromosomes. Polyploidization events are shown with red (duplication) and blue (triplication) dots on the tree branches. **b** TOP- Evolution of white and narrow-leafed lupin genomes from ALK (with 16 CARs) and the lupin ancestral genomes (with 9 and 27 CARS excluding the small CAR #7 in ALK). BOTTOM- Synteny relationships between white (horizontal) and narrow-leafed (vertical) lupin illuminating 17 major chromosomal shuffling events (red and blue arrows for respectively narrow-leafed lupin compared to white lupin and white lupin compared to narrow-leafed lupin).

We retraced the paleohistory of 12 legume genomes including WL and covering the Genistoid, Dalbergioid, Galegoid, and Millettoid clades. Independent blocks of synteny (Supplementary Note 5) allowed the identification of an ancestral legume karyotype (ALK) made of 16 conserved ancestral regions (CARs), Supplementary Data 4. The ancestral genome consists of a minimal shared ancestral genome, which lacks components of the ‘real’ (unknown) ancestral genome that were either lost from all of the investigated descendants and/or retained by only one modern species (Supplementary Data 4). This reveals specific rearrangements (chromosome fusions and fissions) and polyploidization events in the case of soybean and lupins (WL and narrow-leafed lupin, NLL), so that modern legume genomes are composed of a mosaic of 16 shuffled CARs (Fig. 3a, Supplementary Fig. 8). ALK experienced 15 chromosomal fissions and 21 fusions to reach a lupin ancestor of 9 chromosomes that experienced a whole genome triplication to reach a *n* = 27 ancestor intermediate. The modern karyotypes of WL and NLL evolved from the lupin ancestors through 17 major chromosomal shuffling events followed by numerous small-scale rearrangements such as inversions and translocations (Fig. 3b). This comparative genomics-based evolutionary scenario unravels the complex legume paleohistory from the reconstructed ALK, revising previous inferences of legume genomes synteny in delivering the complete catalog of paralogous and orthologous gene relationships between 12 modern legume genomes as well as the ancestral genomes of this major botanical family^21,26–32^.

**Fig. 4:**
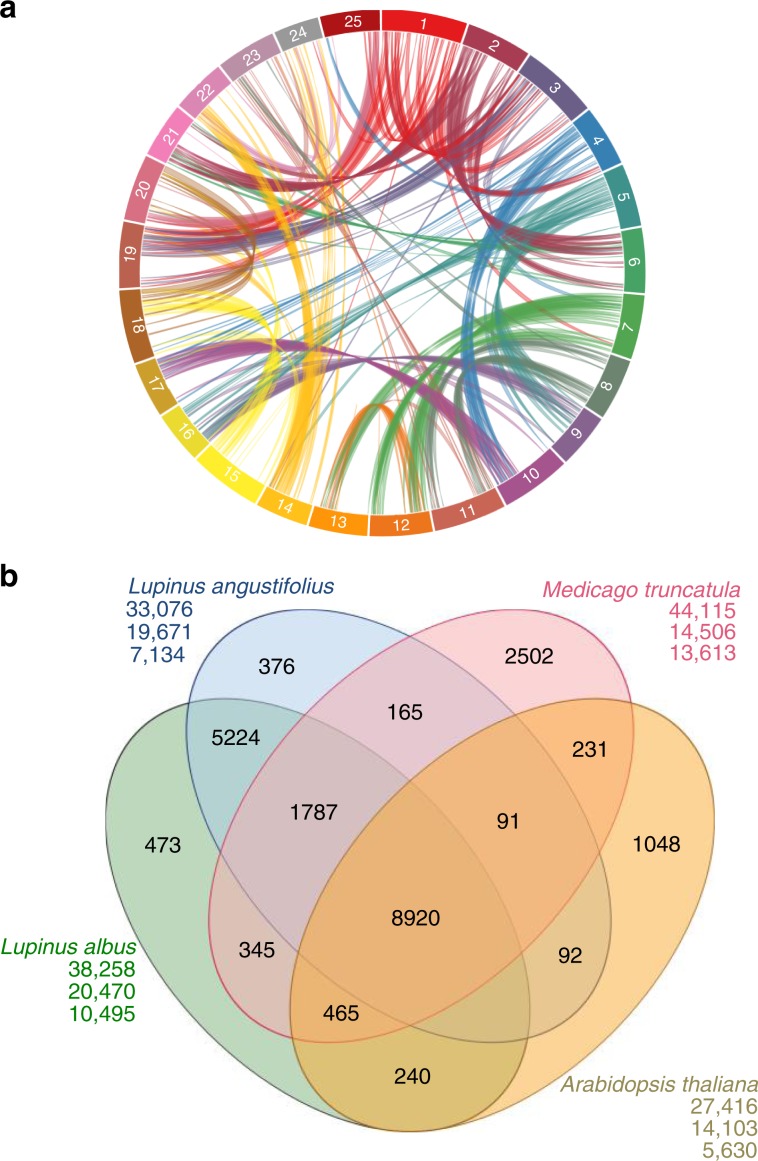
Lupin genome intragenomic duplications and genomic synteny. **a** Syntenic regions inside white lupin genome. The colored lines link colinearity blocks that represent syntenic regions that are bigger than 50 kb. **b** OrthoMCL clustering of white lupin genes with those of *L. angustifolius*, *M. truncatula* and *A. thaliana*.﻿ Numbers in the sections of the diagram indicate the number of clusters (gene groups). ﻿The first number below each species name is the total number of genes of the species, the second number is the number of genes in clusters and the third number is the number of genes that did not cluster.

An intragenomic analysis for segmental duplications (Fig. 4a, Supplementary Note 5) identified 928 blocks bigger than 10 kb pinpointing a triplication feature that can be observed in several chromosome segments (e.g. Chr07, which has two homolog regions with Chr12 and one with Chr13). These blocks have an average size of 65 kb and the largest duplication consists of a 4.1-Mb block shared between Chr18 and Chr20 (Fig. 4a, Supplementary Data 5). Reciprocal pairwise comparisons^33^ of the 38,258 WL genes with 104,607 genes from its closest relative NLL, the model legume *Medicago truncatula*^34^ and *Arabidopsis thaliana* identified 25,615 orthologs clusters (Fig. 4b). 473 out of these groups contain only WL paralog genes (1242 in total), probably as a result of the predicted genome triplication event (Supplementary Data 6). Gene Ontology^35^ terms representation revealed an enriched annotation of serine-type carboxypeptidase activity proteins (GO:0004185), however most of the clusters have no GO term associated (58%, Supplementary Data 6). The WL genome shared highly conserved syntenic blocks with the genome of NLL and *Medicago truncatula*, the reference genome within this family (Supplementary Fig. 9).

### Soil exploration

**Fig. 5:**
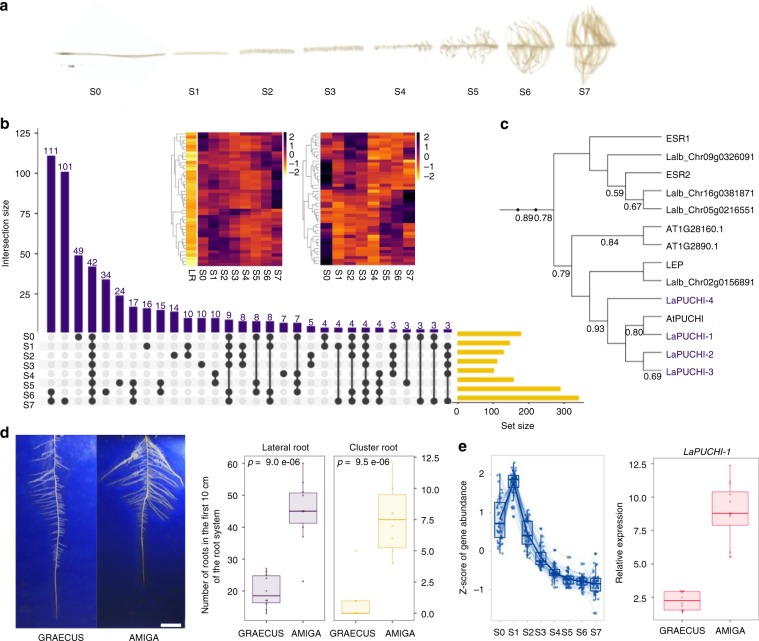
Molecular events of root system establishment in white lupin. **a** 8 developmental stages of cluster root development used for transcriptomic studies, showing the formation of numerous rootlets. **b** Comparative matrix layout of up-regulated genes in the 8 CR segments. Black dots indicate which sample parts (S0 to S7) are grouped and the number of up-regulated genes in the group is indicated on top of each bar. Set size indicates the total number of differentially expressed genes for each sampled fragment. Inserts: heatmap of the 42 genes over expressed in all CR regions (left) and miRNA expressed in the entire CR. **c** Partial phylogenetic tree of AP2/EREBP subfamily B-1 of Arabidopsis and white lupin orthologs, highlighting the 4 PUCHI orthologs in white lupin. **d** Left. Visualization of white lupin root system in 2D. Right. Number of lateral roots and cluster roots in GRAECUS (wild) vs. AMIGA (modern) accessions in the first 10 cm of the root system (*n* = 10). Bar is 5 cm. **e** Left. Expression pattern of 63 genes that are overexpressed in S1 region, which include *LaPUCHI-1*. Right. Relative expression of *LaPUCHI-1* in AMIGA and GRAECUS in top lateral roots of 11-day-old plants. Box edges represent the 0.25 quantile and 0.75 quantile with the median values shown by bold lines. Whiskers extend to data no more than 1.5 times the interquartile range, and remaining data are indicated by dots. Source data underlying Fig. 5d, e are provided as a Source Data file.

Most terrestrial plants can form mycorrhizal symbioses that greatly improve mineral nutrition. Lupins however, lost the ability to form such associations (est. 12–14 My) and the ability to form cluster roots appeared ca. 2.5 My ago^36^. The former was accompanied by the loss of all mycorrhizal specific genes in the WL genome whereas common symbiotic genes remained functional (Supplementary Fig. 10, Supplementary Note 6 and Supplementary Data 7 and 8). This suggests that WL favored a new type of root adaptive mechanism towards nutrient acquisition^11^. Despite the importance of cluster roots, no gene controlling their development has been described to date. We therefore generated a detailed transcriptomic dataset of WL cluster root developmental zones. Our RNA-seq survey (mRNA and miRNA) covered 8 sections of mature clusters that mimic the temporal stages of their development (Fig. 5a and Supplementary Note 7, Supplementary Fig. 11).

We produced a matrix representing all intersections of up-regulated (Fig. 5b, Supplementary Data 9) and down-regulated (Supplementary Fig. 12, Supplementary Data 10) genes in the CR parts. Mature rootlets (S6 and S7) showed the highest number of up-regulated genes, compared to an ordinary lateral root, i.e. devoid of cluster roots (Fig. 5b). This set of genes have a strong enrichment in GO terms associated with membrane components linked with their highly active physiology required to remobilize and acquire phosphate efficiently (Supplementary Figs. 13–15). Interestingly, a list of 42 genes overexpressed in all cluster roots parts (Supplementary Data 11, Fig. 5b detail and Supplementary Fig. 16) showed a strong enrichment in transcription factors (43%) and 9 of them belong to the AP2/EREBP family^37^. This is a large multigene family, and they are key regulators of several developmental processes, like floral organ identity determination, control of leaf epidermal cell identity and control of lateral root development. In WL, we identified 217 genes in this family.

Similarly, a list containing only the genes overexpressed in the S1 region, where CR initiation occurs, is also enriched with transcription factors (6 out of 16). There is an overexpression of 3 genes of AP2/EREBP family that are homologs of *AtPUCHI*, a gene that is required for morphogenesis in the early lateral root primordium of Arabidopsis^38^. We performed an identification of all the homologs genes of the AP2/EREBP subfamily B-1, to which the gene *AtPUCHI* belongs. We identified 20 homologs in the white lupin genome and 4 homologs of the gene *PUCHI* (Fig. 5c, Supplementary Fig. 17).

In parallel, we identified all mature microRNAs that are expressed in cluster root sections. We identified 103 miRNA cluster families, among which 29 are predictions (Fig. 5b detail, Supplementary Data 12 and Supplementary Fig. 18). Some of the known miRNA families that we identified were already described as related with Pi-deficiency response, such as miRNA156, miRNA166, and miRNA2111^39^. We also detected members of miRNA399 family, a key regulator for the phosphate starvation response^40^, that were not detected previously in CR of white lupin^41,42^ (Supplementary Fig. 18a, Supplementary Data 12). We identified that 14 genes out of the 42 overexpressed in all cluster root zones are possible miRNA targets, including 5 transcription factors (Supplementary Data 11). Likewise, in the group of 16 genes that are only overexpressed in the region S1, we identified 5 genes that are targets of the detected miRNAs, comprising transcription factors *LaWRKY* (Lalb_Chr07g0182001) and *LaPUCHI-3* (Lalb_Chr18g0055601).

A possible impact of domestication on WL soil exploration capacity was investigated using a 2D-phenotyping platform. We identified that the root system architecture of AMIGA develops earlier than the wild-relative GRAECUS as a result of a strong increase in lateral and cluster root number in the upper part of the root system (Fig. 5d, Supplementary Fig. 19). This difference was correlated with an increased level of expression of the regulatory gene *LaPUCHI-1* (Fig. 5c, e), whose genetic sequence is identical in AMIGA and GRAECUS. A list of candidate genes selected on their high induction level at the S1 stage (*LaCLE1*, *LaMYB1*, *LaPEP1*, *LaPME41*, and *LaSTART*), also showed a higher expression level in AMIGA compared to its wild relative GRAECUS (Supplementary Fig. 20). This suggests that activation of key regulatory genes may trigger the early establishment of the root system, a trait that has been characterized in other crops to be key for more efficient phosphate acquisition (*e.g*. the *pup1* QTL in rice where the *PSTOL1*^43^ gene controls early root system establishment).

### Seed quality

**Fig. 6:**
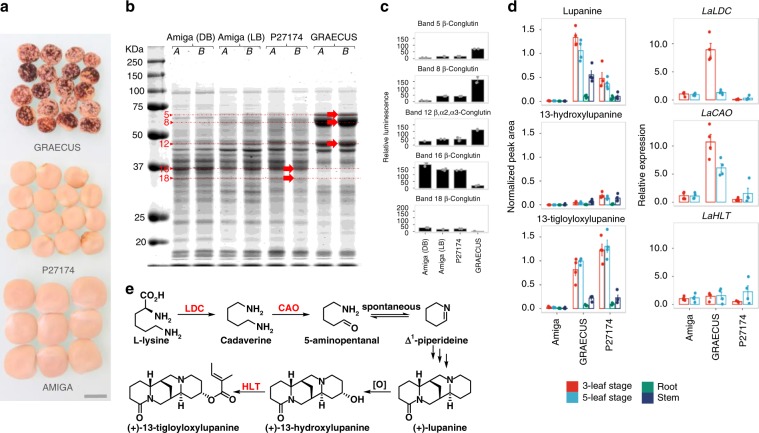
White lupin seed protein and leaf alkaloid content. **a** Grain appearance in GRAECUS (wild-type), Ethiopian landrace P27174 and *cv*. AMIGA. Bar is 0.5 cm. **b** Seed protein composition of AMIGA (DB, dark brown seeds; LB, light brown seeds), P27174 and GRAECUS (A and B: two independent extractions of proteins using Tris-SDS, separation in 12% SDS-PAGE). The bands extracted for MS/MS analysis are highlighted with red arrows. **c** Protein content quantification of each band extracted for each accession expressed in normalized gel volume. Data are presented as means ± SD, *n* = 2. **d** Abundance of the three major alkaloids in young leaf (3- and 5-leaf growth stage), stem and root tissues of the three accessions of *L. albus* as measured by LC-MS, and expression of the known alkaloid biosynthesis genes in young leaves as measured by qRT-PCR. *n* = 5 for AMIGA and *n* = 4 for P27174 and GRAECUS. **e** Putative biosynthetic pathway of tetracyclic quinolizidine alkaloids in lupins. Characterized steps and respective enzyme names are marked in red. More information about these analyses is described in Supplementary Note 9. LDC: lysine decarboxylase; CAO: copper amine oxidase; HLT: 13-hydroxylupanine O-tigloyltransferase. Data are presented as means ± SD, *n* = 4. Source data underlying Figs. 6b, d are provided as a Source Data file.

We compared seed protein composition between the AMIGA reference accession, the Ethiopian landrace (P27174) and the wild GRAECUS relative by quantitative 1D gel analysis followed by mass spectrometry identification of specific protein bands (Fig. 6b, Supplementary Note 8, Supplementary Fig. 21 and Supplementary Data 13). AMIGA seeds displayed a disappearance of high molecular weight ß-conglutins (Fig. 6c, Supplementary Data 14), which are precursor forms normally synthesized in developing cotyledons to give rise to mature polypeptides of lower molecular weight. Their degradation starts as part of the germination process and the appearance of simpler forms in the domesticated variety AMIGA could be linked with its increased seed vigor^44^. The long chain ß-conglutins present in the wild accession is also associated with a high allergenicity of lupin seeds^45^, a trait that might have been counter-selected during domestication.

Measurement of the abundance of key alkaloids in various organs revealed that the modern accession AMIGA has very low levels of lupanine, 13-hydroxylupanine and 13-tigloyloxylupanine (Fig. 6d) but we were not able to match this lack of alkaloids with reduced expression of candidate genes involved in the pathway (Fig. 6d, e, Supplementary Data 15). Instead, we identified a list of candidate genes on Chr18 as associated to the *pauper* QTL that is responsible for the sweet trait of modern WL accessions, including AMIGA^17,46^ (Supplementary Note 9). This region of 958 kb contains 66 genes amongst which several strong candidates encoding for protein with enzymatic activity such as cinnamoyl-CoA reductase and acyltransferases (Supplementary Data 16). Further functional characterization of these genes will certainly lead to a better understanding of the alkaloid content reduction observed in modern accessions and provide the genetic mechanisms underlying the *pauper* locus.

## Discussion

WL is a pulse that is becoming more and more attractive to consumers seeking plant-based sources of proteins^47^. The large size of its seeds is considered to be a result of direct selection both for consumer’s preferences (when used as a snack for human consumption) and as an adaptation to its Mediterranean environment (larger seeds display early vigor that is needed to complete their lifecycle before summer drought)^48^. The adaptive capacity of WL is therefore noticeable not only in the large seed size, but also in their early root system that derives from it^49^. Seed vigor allows a quick establishment of the seedling root, a trait that has been identified as a key parameter for the *pup1* QTL in rice resistance to low phosphate^43^. Interestingly, we report here that WL modern accessions present a large seed size with a specific protein composition as well as a strong capacity for early soil exploration through lateral and cluster root formation, compared to wild accessions.

The striking ability of WL to form cluster roots is shared by plants from 10 different botanical families^50^ (including monocots from the Cyperaceae family). This raises the question whether these developmental structures appeared independently several times during evolution due to the lack of mycorrhizal associations in these species or whether they were present in a common ancestor and subsequently lost in most plants. The high-quality genome sequence of WL, the only annual crop producing cluster roots and showing a reduced need for phosphate fertilizers, will help to understand the molecular mechanisms behind these adaptations. Since phosphate is a limited resource^51^, improved phosphate acquisition could represent an important trait for the future improvement of nutrient acquisition in other crops.

Although WL seeds already present protein contents that are similar or higher than soybean, it remains a crop with fluctuating yields. Also, the ability to use a large gene pool for breeding has been hampered by the presence of alkaloids in most wild accessions. The characterization of the alkaloid pathway and the identification of genes responsible for the sweet trait of cultivated accessions will certainly help to take advantage of the wide variability available in this species. The high-quality reference genome sequence and companion resources of WL will help reinforce breeding programs aimed at improving yield stability and maintaining a low content of anti-nutritional alkaloids.

## Methods

### Genome assembly and annotation of *L. albus* L. cv. AMIGA

A meta-assembly strategy similar to the one developed to assemble the Rosa genome^52^ was applied. The Supplementary Data 1 provides details of the different steps of the process including data, software and the evolution of the metrics of the assembly. Firstly, three assemblies were performed with CANU^15^ using different level of stringency (errorRate = default, 0.015 and 0.025 respectively). Corrected reads generated by CANU^15^ were also used to run FALCON^16^. The graph of overlaps of FALCON was filtered using three different sets of parameters of the program til-r^52^, in order again to generate alternative assemblies with different level of stringency.

The N50 metrics of the primary assemblies ranged from 1.6 to 7.1 Mb. The sequences of these six primary assemblies were first transformed in pseudo long reads of 100 kb with an overlap of 50 kb. Then, the pseudo long reads were assembled with CANU 1.6 in the mode –trim-assemble to enable the trimming of sequence ends specific to a single primary assembly.

The meta-assembly result displays a N50 of 8.9 Mb in only 129 contigs. The Bionano hybridScaffold.pl software was run in order to scaffold the contigs of the meta-assembly using the Bionano Optical map (N50 2.3 Mb). In all, 15 putative breakpoints were identified and corrected by the scaffolder. The scaffolds were polished twice, firstly using arrow and the pacbio raw data mapped with blasr, then with Pilon^53^ using 100x of illumina data mapped with glint software (http://lipm-bioinfo.toulouse.inra.fr/download/glint/). Finally the pseudo-chromosomes were obtained with ALLMAPS^54^ by scaffolding the polished scaffolds with the high density genetic map^17^. A total 96.2% of the data were anchored on the linkage map and 95.3% were oriented (Supplementary Fig. 1). Detailed information about the genome annotation is presented in Supplementary Note 1.

### Evaluation of AMIGA heterogeneity

In order to evaluate the heterogeneity of cv. AMIGA, a bulk of 90 AMIGA plants was resequenced using Illumina HiSeq300, with paired-end 2 × 150 bp reads. This produced 193,734,276 clean reads corresponding to a total of 64.47x depth. Cutadapt^55^ has been used to remove Illumina Truseq adapter from the sequencing data and to remove bases with a quality score lower than 30, in both 5′ and 3′ end of the reads. Reads with a length lower than 35 have been discarded. We used BWA-MEM version 0.7.17^56^ to map the resequencing reads to the white lupin reference genome. Picard tools (https://github.com/broadinstitute/picard/issues) have been used to detect and remove PCR and Optical duplicates. We then used GATK 4.0^57^ HaplotypeCaller tool to call variants. This identified ca. 300,000 SNPs without filtering the data. All the SNPs are evenly distributed on the 25 chromosomes and contigs. We generated a VCF file with this information, available in the white lupin Genome Browser.

### Assembly of mitochondrial and chloroplastic genomes

A de novo assembly protocol was used to assemble both cytoplasmic genomes. They were generated using NOVOPlasty 3.2^58^, by using the aforementioned Illumina reads, after adapter-removing step. Assembly of chloroplastic genome (plastome) was performed using as reference a publicly available *L. albus* plastome (GenBank accession NC_026681) and mitochondrial genome (mitogenome) was assembled using the Vicia faba mitogenome (GenBank accession KC189947) as reference. The assemblies were checked with Geneious v. 9.1.9 mapper tool by mapping Illumina and PacBio reads. For the mitogenome annotation we used as reference other legume species with available annotated mitogenomes on NCBI, whereas for the plastome annotation we used as a reference the available L. albus plastome. The assembly of the cytoplasmic genomes resulted in single circularized contigs of 151,915 bp for the plastome (Supplementary Fig. 22) and 405,575 bp for the mitogenome (Supplementary Fig. 23).

### Annotation of repeats

Identification and characterization of moderately to highly repeated genomic sequences was achieved by graph-based clustering of genomic Illumina reads using RepeatExplorer2 pipeline^59^. A total of 1,144,690 of 150 bp paired reads, representing ~0.5× genome coverage, were used for the clustering and the 145 largest clusters with genome proportions of at least 0.01% were examined in detail. Clusters containing satellite DNA (satDNA) repeats were identified based on the presence of tandem sub-repeats within their read or assembled contig sequences with TAREAN^60^. Genome-wide TE repeat annotation was performed using the DANTE (Domain-based ANnotation of Transposable Elements) tool^60^. Consensus sequences of satDNA repeats and rDNA genes were used to perform genome-wide annotation of satDNA and rDNA arrays using the Geneious v. 9.1.8 annotation tool (https://www.geneious.com). The generated GFF3 files were further incorporated on the *L. albus* genome browser.

### Chromosome preparation for in situ hybridization

Chromosome preparations for in situ hybridization analysis were conducted as described in Marques et al.^61^. with modifications. First, young roots (pre-treated with 8-hydroxyquinoline 2 mM for 3–5 h at room temperature) and anthers were fixed in 3:1 (ethanol:acetic acid) for 2–24 h. The fixed tissues were treated with an enzyme mixture (0.7% cellulase R10, 0.7% cellulase, 1.0% pectolyase, and 1.0% cytohelicase in 1× citric buffer) for 1 h at 37 °C. Material was then washed twice in water and fragmented in 7 μl of 60% freshly prepared acetic acid into smaller pieces with the help of a needle on a slide. Another 7 μl of 60% acetic acid was added, and the specimen was kept for 2 min at room temperature. Next, a homogenization step was performed with an additional 7 μl 60% acetic acid and the slide was placed on a 55-°C hot plate for 2 min. The material was spread by hovering a needle over the drop without touching the hot slide. After spreading of cells, the drop was surrounded by 200 μl of ice-cold, freshly prepared 3:1 (ethanol:acetic acid) fixative. More fixative was added and the slide was briefly washed in fixative, then dipped in 60% acetic acid for 10 min and dehydrated in 96% ethanol. The slides were stored until use in 96% ethanol at 4 °C.

### Probe preparation and fluorescence in situ hybridization

FISH probes were obtained as 5′-Cy3 or 5′-FAM-labeled oligonucleotides (Eurofins MWG Operon, http://www.eurofinsdna.com), or were PCR-amplified as described below. All DNA probes, except oligonucleotides, were labeled with Cy3- or Alexa 488-dUTP (Jena Bioscience) by nick translation, as described in Kato et al.^62^. The sequences of all oligonucleotides and primers are listed in Supplementary Table 5. FISH was performed as described in Marques et al.^61^. Probes were then mixed with the hybridization mixture (50% formamide and 20% dextran sulfate in 2× SSC), dropped onto slides, covered with a cover slip and sealed. After denaturation on a heating plate at 80 °C for 3 min, slides were hybridized at 37 °C overnight. Post-hybridization washing was performed in 2× SSC for 20 min at 58 °C. After dehydration in an ethanol series, 4′,6–diamidino-2–phenylindole (DAPI) in Vectashield (Vector Laboratories, http://www.vectorlabs.com) was applied. Microscopic images were recorded using a Zeiss Axiovert 200 M microscope equipped with a Zeiss AxioCam CCD. Images were analyzed using the ZEN software (Carl Zeiss GmbH). Primer and oligo-probes information is presented in Supplementary Note 3, Supplementary Table 4.

### Labeling of tandem repeat and retroelement fragments

Fragments for probe labeling were amplified using genomic DNA from *L. albus* using the forward and reverse primers as supplied on Supplementary Table 4. Eight PCR reactions for each target repeat were performed in 50 μL reaction volume containing 100 ng of gDNA, 1 μM primers, 1 × PCR buffer, 0.2 mM dNTPs, and 1U of Taq polymerase (Qiagen). Thirty-five amplification cycles with proper conditions for each set of primers were run. PCR reactions were sampled, purified and concentrated using Wizard® SV Gel and PCR Clean-Up System (Promega). Sanger sequencing confirmed correct amplification of PCR fragments. After confirmation, the PCR products containing the same class of repeat were collected and used for probe labeling by nick translation as described above.

### LalbCENH3-ChIP and ChIP-seq analyses

Chromatin immunoprecipitation experiments were done with Abcam ChIP Kit - Plants (ab117137) following the manufacturer’s instructions. First, 1 g of young *L. albus* cv. AMIGA leaves were collected and cross-linked with formaldehyde 1% for 15 min on ice. Leaves were then ground in liquid nitrogen and sonicated using a Diagenode Sonicator. Sonicated chromatin-DNA ranging from 200–1000 bp was immunoprecipitated using anti-LalbCENH3 (lifetein.com, 1:300 dilution). Immunoprecipitated DNA samples and, as a control, an input chromatin DNA samples (3–7 ng for each sample) were sent for ChIPseq at BGI. The original ChIPseq sample data are available at White Lupin Genome Website (http://www.whitelupin.fr). To identify repeats associated with CENH3-containing chromatin, reads from the ChIPseq experiment obtained by sequencing DNA from isolated chromatin prior to (the input control sample) and after immunoprecipitation with the CENH3 antibody (1:200 dilution, the ChIP sample) were separately mapped to the repeat clusters. The mapping was based on read similarities to contigs representing individual clusters, using BLASTn with parameters ‘-m 8 -b 1 -e 1e-20 -W 9 -r 2 -q -3 -G 5 -E 2 -F F’ and custom Perl scripts for parsing the results. Each read was mapped to a maximum of one cluster, based on its best similarity detected among the contigs. Ratio of ChIP/input reads assigned to individual clusters was then used to identify repeats enriched in the ChIP sample as compared to the input.

### Data generation with short-reads technology

We selected 14 white lupin accessions to evaluate a broader range of the genetic diversity and determine population structure and linkage disequilibrium. More information about these accessions can be found in Supplementary Note 4. Young leaves of 30 plants were used to extract genomic DNA of each accession using the QIAGEN Genomic-tip 100/G kit following the supplier’s recommendations. The accessions were sequenced using Illumina technology using paired-end 2 × 150 bp short-reads. It was generated a total of 310.95 Gb of data with average sequencing depth of 45.99× (Supplementary Table 8).

### Mapping and SNP detection

Cutadapt^55^ was used to remove Illumina Truseq adapters from the sequencing data and to remove bases with a quality score lower than 30, in both 5’ and 3’ end of the reads. Reads with a length lower than 35 were discarded. We then used BWA-MEM version 0.7.17^56^ to map the resequencing reads from all 15 genotypes to the white lupin reference genome. PCR and Optical duplicates have been detected and removed using Picard Tools. After that, GATK 4 HaplotypeCaller tool have been used in emit-ref-confidence GVCF mode to produce one gvcf file per sample. These files have been merged using GATK CombineGVCFs. Finaly, GATK GenotypeGVCFs have been used to produce a vcf file containing variants from all the 15 samples. This identified a total of 6,620,353 SNPs/indel. After filtering for minimum allele frequency of 0.15 and heterozygosity frequency of 0–0.2, 2,659,837 SNPs were retained to further analysis.

### Phylogenetic analysis and population structure

The genetic distance matrix was calculated based on identity-by-state similarity method and an average cladogram constructed using neighbor-joining algorithm implemented on TASSEL 5.2.51^63^. Then, a phylogenetic tree was prepared using the iTOL v 4.3^64^. A principal component analysis (PCA) was also performed in R (http://www.R-project.org/) function ‘prcomp’. A Bayesian model-based clustering method implemented with STRUCTURE v2.3.4^22^ was used to investigate the population structure using all the filtered SNPs. The program was run 10 times for each K value, ranging from 1 to 5, with a 1000 burn-in time and 1000 iterations. The optimal *K* value was determined based on the ΔK from the Structure Harvester v0.6.94^65^ program, through Evanno’s test^23^.

### De novo assembly of GRAECUS and P27174

Long-read sequencing was realized using Oxford Nanopore technology, using a GridION 18.04.1-0, with a software Minknow 1.10.24-1 at platform at Get-PlaGe core facility (INRA, Toulouse, France). High MW DNA was used to prepare a library with the Ligation Sequencing Kit 1D (sqk-lsk109). DNA was sequenced using a single ONT MinION R9.4 flowcell (FLO-MIN106) for 48 h and base-calling was performed using Albacore 2.1.10-1. This produced 1,280,206 sequences for GRAECUS, corresponding to 12.45 Gb of data with a N50 length of 13.6 Kb (27.6 x of sequencing depth). For the accession P27174 this produced a total of 1,738,579 reads corresponding to 14.59 Gb of data with N50 length of 11.8 Kb (32.36 x of sequencing depth). The de novo assembly of the two genotypes were performed using CANU^15^. For P27174-4, two round of correction have been made prior to the assembly step, using the parameters correctedErrorRate = 0.16 and corMaxEvidenceErate = 0.15. For GRAECUS, only one round of correction have been made, using minOverlapLength = 400, correctedErrorRate = 0.16 and corMaxEvidenceErate = 0.15. The Illumina paired-end data described in 3.1 were used to polish two times the two genome assemblies using Pilon^53^. BUSCO v 3.0.0^66^ was run on the set of predicted transcripts. The assessment software detected for GRAECUS 96.8% of complete gene models (1142 complete single copy and 188 duplicated respectively) plus 9 additional fragmented gene models. For P27174 97.8% of complete gene models (1125 complete single copy and 220 duplicated respectively) plus 4 additional fragmented gene models. Structural variation of these two accession were performed using Assemblytics^24^ based on whole genomes alignments generated with MUMmer^25^. Details of the de novo genome assembly and analysis of structural variation of these two accessions are provided in Supplementary Note 4.

### Evolutionary analysis of the legume genomes

The proposed evolutionary scenario was obtained following the method described in Pont et al.^67^ based on synteny relationships identified between *L. albus* and other 11 legume species. Briefly, the first step consists of aligning the investigated genomes to define conserved/duplicated gene pairs on the basis of alignment parameters referenced to as Cumulative Identity Percentage (CIP) and Cumulative Alignment Length Percentage (CALP). The second step consists of clustering or chaining groups of conserved genes into synteny blocks (excluding blocks with less than 5 genes) corresponding to independent sets of blocks sharing orthologous relationships in modern species. In the third step, conserved gene pairs or conserved groups of gene-to-gene adjacencies defining identical chromosome-to-chromosome relationships between all the extant genomes are merged into Conserved Ancestral Regions (CARs). CARs are then merged into protochromosomes based on partial synteny observed between a subset (not all) of the investigated species. The ancestral karyotype can be considered as a ‘median’ or ‘intermediate’ genome consisting of protochromosomes defining a clean reference gene order common to the modern species investigated. From the reconstructed ancestral karyotype an evolutionary scenario was then inferred taking into account the fewest number of genomic rearrangements (including inversions, fusions, fissions, translocations), which may have operated between the inferred ancestors and the modern genomes. Additional information is provided in Supplementary Note 5.

### Genome synteny and intragenomic collinearity

To identify intragenomic colinearity blocks inside the white lupin genome we used SynMap (CoGe, www.genomevolution.org) using homologous CDS pairs using the following parameters: Maximum distance between two matches (-D): 20; Minimum number of aligned pairs (-A): 10; Algorithm ‘Quota Align Merge’ with Maximum distance between two blocks (-Dm): 500.

### Gene family identification

We used a comparative analysis to examine the conservation of gene repertoires among orthologs in the genomes of white lupin, narrow-leafed lupin (v1.0) *M. truncatula* (Mt4.0) and *Arabidopsis thaliana* (TAIR10). First, we aligned all-to-all proteins using BLASTP (e-value of 1e^−5^). Genes were then clustered using OrthoMCL (1.4) implemented in OrthoVenn^33^ with a Markov inflation index of 1.5 and a minimum e-value of 1e^−15^.

### Spatial transcriptome for mRNA and small RNA

Ten cluster roots coming from four grown plants were harvested after 12 days of culture and dissected in eight parts of 0.5-cm from the apex of the lateral root that carries the cluster root (Supplementary Note 7). As control, 1-cm of lateral roots without cluster roots, sampled 1-cm away from the primary root, were collected. Four biological replications were produced for each experiment. Total RNA was extracted from all frozen samples using the Direct-zol RNA MiniPrep kit (Zymo Research, Irvine, CA) according to the manufacturer’s recommendations.

For mRNA sequencing, 36 independent root RNA-seq libraries were constructed using Illumina TruSeq Stranded mRNA Sample Preparation Kit (Illumina Inc.) according to the manufacturer’s protocol. The samples were sequenced using paired-end sequencing was performed generating paired-ended 2 × 150 bp reads using TruSeq SBS kit v3 sequencing chemistry (Illumina Inc.) in one lane of Illumina NovaSeq instrument according to the manufacturer’s instructions. A total of 2,048,118,650 paired-end reads of 150 pb were sequenced using an Illumina NovaSeq6000 Sequencer. To remove low quality sequences, the RNA-seq reads were checked and trimmed using Cutadapt^55^ with a minimum quality score of 30 in both 3′ and 5′ end, with the nextseq-trim option enabled. Illumina TruSeq adapter sequences have also been removed. The resulting reads shorter than 35 pb have been discarded. The quality checked RNA-seq reads were then mapped on white lupin reference genome using Hisat2^68^ software. Transcripts were assembled and quantified using Stringtie software. Gene counts were extracted and imported in the R package DESeq2^69^. These counts have been normalized according to the size factor computed by DESeq2.

For small RNA sequencing, 24 independent root RNA-seq libraries were constructed using NEXTflex™ Small RNA-Seq kit according to the manufacturer’s protocol. All small RNA libraries were sequenced on an Illumina NextSeq 500 sequencing platform, using a single-end, 75 nt read metric instrument according to the manufacturer’s instructions. A total of 460,506,072 reads of 75 nt were sequenced. Small RNA-seq reads were trimmed using Cutadapt version 1.11^55^ to remove remnants of the following 3′-adapter sequence. Details on the trimming, assembly, differential expression analysis, and miRNA family identification can be found in Supplementary Note 7.

### Root sampling and expression analysis of cluster root genes

We sampled 2–3 cm of lateral roots 1-cm away from the primary root in the top 5 cm (cluster root region, CRR) and at 10 cm from the top (regular lateral root region, NLR) of the root system of AMIGA and GRAECUS plants, 11 days after germination. Three CRR and 3 NLR independent samples were collected for each accession. Total RNA from these samples was extracted using the Direct-zol RNA MiniPrep kit (Zymo Research, Irvine, CA) according to the manufacturer’s recommendations. RNA concentration was measured on a NanoDrop (ND1000) spectrophotometer. Poly(dT) cDNA were prepared from 2 μg total RNA using the revertaid First Strand cDNA Synthesis (Thermo Fisher). Gene expression was measured by quantitative Real Time - Polymerase Chain Reaction (qRT-PCR) (LightCycler 480, Roche Diagnostics, Basel, Switzerland) using the SYBR Premix Ex Taq (Tli RNaseH, Takara, Clontech, Mountain View, CA) in 384-well plates (Dutscher, Brumath, France). Target quantifications were performed with specific primer pairs described on the Supplementary Table 11. Expression levels were normalized to *LaHelicase* (Lalb_Chr13g0304501). All qRT-PCR experiments were performed in technical quadruplicates. Relative gene expression levels were calculated according to the ΔΔCt method^70^, using as a calibrator the NLR samples. All experiments were performed as three biological replicates.

### Reporting summary

Further information on research design is available in the Nature Research Reporting Summary linked to this article.

## Supplementary information

Supplementary Information

Peer Review File

Reporting Summary

Description of Additional Supplementary Files

Supplementary Data 1-16

## Data Availability

Data supporting the findings of this work are available within the paper and its Supplementary Information files. A reporting summary for this Article is available as a Supplementary Information file. The datasets generated and analyzed during the current study are available from the corresponding author upon request. Full genomic, RNAseq, ChIPseq and raw sequence data are publicly available for download on the White Lupin genome portal [www.whitelupin.fr] that contains a Genome Browser, Expression tools and a Sequence retriever. This Whole Genome Shotgun project has been deposited at DDBJ/ENA/GenBank under the accession WOCE00000000. The version described in this paper is WOCE01000000. The ChIPseq data have been deposited at NCBI under the accession PRJNA593700. The RNAseq data have been deposited at NCBI under the accession PRJNA575804 (10 organs transcriptomics,) and PRJNA593912 (cluster root spatial transcriptomics,). The source data underlying Figs. 1d, 2b, 5d, 5e, and 6b, d are provided as a Source Data file.

## Source Data

Source Data
