# Supplementary material for: High-quality genome sequence of white lupin provides insight into soil exploration and seed quality
Source: Nat Commun. 2020 Jan 24;11:492. doi: 10.1038/s41467-019-14197-9 (PMC6981116; doi:10.1038/s41467-019-14197-9)
Supplement: Supplementary file 1 — Supplementary Information [file 41467_2019_14197_MOESM1_ESM.pdf]

High-quality genome sequence of white lupin provides insight into soil exploration and seed quality

Hufnagel *et al.*

Supplementary Note 1. White lupin genome sequencing and assembly

Single-molecule real-time and short-read sequencing

Genomic DNA was extracted from young leaves of a one single seed descendent (S2 generation) derived plant of *Lupinus albus* cv. AMIGA (from Florimond Desprez, France) using the QIAGEN Genomic-tip 100/G kit following the supplier's recommendations. Sequencing was sub-contracted at Gentyane core facility (INRA, Clermont-Ferrand, France), using a PacBio Sequel E406 with 30 SMRT Cells (V2 chemistry, 6h run), yielding a total of 94.2 Gbp of data and estimated genome coverage of 164x (Supplementary Table 1).

DNA genomic from the same extraction described above was used to generate short-reads using one lane of Illumina HiSeq300, with paired-end 2x150 bp reads at Get-PlaGe core facility (INRA, Toulouse, France). It produced 794,235,810 clean reads corresponding to a total of 119 Gb of reads (208x depth).

Optical maps

Leaves were collected from two white lupin plants (three-leaf seedling stage) grown in the dark for 3 days and originating from the same parent as the plant used for long and short read sequencing. High Molecular Weight DNA was extracted with the IrysPrep® Plant Tissue DNA Isolation kit (BioNano Genomics) from 2 g of fresh leaves following the supplier's recommendations. To preserve the DNA integrity and obtain long DNA fragments, nuclei were embedded in agarose before DNA extraction. A first draft assembly generated from the PacBio reads using CANU[1] was analysed with the Label Density CalculatorTM 1.3.0 software (Bionano Genomics) to assess the frequency of recognition sites for different nicking enzymes. We chose the Nb.BssSI nicking enzyme (New England Biolabs), which had a theoretical label density of 10 labels for 100 kb. DNA molecules were nicked with Nb.BssSI and labelled according to the instruction provided with the IrysPrep® NLRS Labelling Kit (BioNano Genomics). The labelled DNA molecules were stretched on IrysChip® V2 (BioNano Genomics) and pictured on the Irys® System (BioNano Genomics). Raw image data were converted by the AutoDetectTM 2.1.4.9159 software (BioNano Genomics) into .bnx files corresponding to digital representations of the DNA

molecules carrying the motif-specific label pattern. The *de novo* assembly algorithm from BioNano SolveTM data analysis software aligned the digital molecules for the construction of a consensus genome map. 293 maps with a N50 of 2.3 Mb were produced using a selection of molecules above 150 kb. The consensus genome optical map size is 455Mb.

K-mer genome size estimation

We used the 119 Gb data from the 150bp paired-end Illumina library to perform genome size estimation. The library was submitted to 17-mer frequency distribution analysis using Jellyfish[2] and GCE (Genomic Character Estimator - v1.0.0)[3]. A total of 10,4,671,784,125 17-mers were counted. Given the unique k-mer depth of 174, we calculated that the genome size = KmerCount/Depth = 573.769 Mb. This value is close to the 567 ± 21 Mb described previously based on flow cytometry[4,5].

Genome assembly

A meta-assembly strategy similar to the one developed to assemble the Rosa genome[6] was applied. The Supplementary Table 1 provides details of the different steps of the process including data, software and the evolution of the metrics of the assembly. Firstly, three assemblies were performed with CANU[1] using different level of stringency (errorRate=default, 0.015 and 0.025 respectively). Corrected reads generated by CANU were also used to run FALCON[7]. The graph of overlaps of FALCON was filtered using three different sets of parameters of the program til-r [6], in order again to generate alternative assemblies with different level of stringency.

The N50 metrics of the primary assemblies ranged from 1.6 to 7.1Mb. The sequences of these six primary assemblies were first transformed in pseudo long reads of 100kb with an overlap of 50kb. Then, the pseudo long reads were assembled with CANU 1.6 in the mode –trim-assemble to enable the trimming of sequence ends specific to a single primary assembly.

The meta-assembly result displays a N50 of 8.9Mb in only 129 contigs. The Bionano hybridScaffold.pl software was run in order to scaffold the contigs of the meta-assembly using the Bionano Optical map (N50 2.3Mb). 15 putative breakpoints were identified and corrected by the scaffolder. The scaffolds were polished twice,

firstly using arrow and the PacBio raw data mapped with blasr, then with Pilon[8] using 100x of Illumina data mapped with glint software (http://lipm-bioinfo.toulouse.inra.fr/download/glint/).

Finally the pseudo-chromosomes were obtained with ALLMAPS[9] by scaffolding the polished scaffolds with the high density genetic map[10]. A total 96.2% of the data were anchored on the linkage map and 95.3% were oriented (Supplementary Fig. 1).

Assembly and annotation of mitochondrial and chloroplastic genomes

A de novo assembly protocol was used to assemble both cytoplasmic genomes. They were generated using NOVOPlasty 3.210, by using the aforementioned Illumina reads, after adapter-removing step. Assembly of chloroplastic genome (plastome) was performed using as reference the already available *L. albus* plastome (GenBank accession NC_026681) and mitochondrial genome (mitogenome) was assembled using the *Vicia faba* mitogenome (GenBank accession KC189947) as reference. The assemblies were checked with Geneious v. 9.1.9 mapper tool by mapping Illumina and PacBio reads. For the mitogenome annotation we used as reference other legume species with available annotated mitogenomes on NCBI, whereas for the plastome annotation we used as a reference the available *L. albus* plastome. The assembly of the cytoplasmic genomes resulted in single circularized contigs of 151,915 bp for the plastome (Supplementary Fig. 20) and 405,575 bp for the mitogenome (Supplementary Fig. 21).

Supplementary Note 2. Genome annotation

Generation of Illumina RNA-seq data from 10 white lupin tissues

Plants of *L. albus* cv. AMIGA were grown in growth chambers under controlled conditions (16 h light/8 h dark, 25°C day/20°C night, 65% relative humidity and PAR intensity 200 µmol m^{-2} s^{-1}) at B&PMP Institute facilities, Montpellier, France. Tissue was flash frozen in liquid nitrogen and stored at -80 °C. In total, 10 white lupin different samples were isolated for generation of RNA-seq data:

- <u>*Root tissues*</u>: Root tissues were sampled from plants grown in hydroponics conditions. The hydroponic solution was modified from previous medium[11] with (+P, 400 KH2PO4 µM) or without phosphate (-P, K2SO4 200 µM), according to the following composition: MgSO4, 54 µM; Ca(NO3)2 400 µM; Na-Fe-EDTA 10 µM; H3BO3 2.4 µM; MnSO4 0.24 µM; ZnSO4 0.1 µM; CuSO4 0.018 µM and Na2MoO4 0.03 µM. Six different types of root tissues were sampled:
    - AMIGA Roots –P: entire root system of AMIGA grown under –P conditions 20 days after germination;
    - AMIGA Roots +P: entire root system of AMIGA grown under +P conditions 20 days after germination;
    - AMIGA Lateral Roots: mixture of lateral roots of AMIGA (without cluster roots), grown under –P and +P conditions 20 days after germination;
    - AMIGA Primary Roots: mixture of AMIGA primary roots grown under –P and +P conditions 20 days after germination;
    - AMIGA Cluster Roots: mixture of entire cluster roots of AMIGA grown under –P condition 20 days after germination;
    - AMIGA nodulated roots: entire root system of AMIGA in different stages of nodulation after inoculation with bacteria *Bradyrhizobium lupini* strain LL13. Plants were sampled 21 days after germination (18 days after inoculation.
- <u>*Vegetative tissues*</u>:
    - AMIGA Leaves: mixture of AMIGA leaves in different stages of development sampled for plants grown in hydroponic solution (as described in the above section) with +P and –P conditions;

- AMIGA Flower: mixture of entire flowers of 6 AMIGA plants grown in 2 L pots;
- AMIGA Pods: mixture of pods of 3 AMIGA plants grown in 2 L pots in controlled conditions.

- <u>*Seeds:*</u>
  - AMIGA seeds: mixture of tissue from 10 mature seeds of AMIGA.

Total RNA was extracted from all frozen samples using the Direct-zol RNA MiniPrep kit (Zymo Research, Irvine, CA) according to the manufacturer's recommendations. RNA-seq libraries were constructed and sequenced at Montpellier GenomiX facilities. The Illumina TruSeq Stranded mRNA Sample Preparation Kit (Illumina Inc.) was used according to the manufacturer's protocol. One lane of Illumina HiSeq 2500 instruments according to the manufacturer's instructions. Paired-end sequencing was performed generating paired-ended 2 x 125 bp reads using TruSeq SBS kit v3 sequencing chemistry (Illumina Inc.). This produced 69.25 Gb of data (276.99 M reads) in an average of 6.9 Gb of data per sample (Supplementary Table 2).

Prediction of gene models

Protein and non-protein coding gene models were predicted using the integrative EuGene pipeline[12] (http://eugene.toulouse.inra.fr/Downloads/egnep-Linux-x86_64.1.4.tar.gz). Three protein databases were aligned to contribute to translated regions detection: i) Swiss-Prot – October 2016 ii) a plant subset of Uniprot proteins – October 2016 and iii) the proteome of *Medicago truncatula*, release 5.1.6[13]. Proteins with similarities to REPBASE[14] were removed from datasets prior alignment. Chained alignments spanning less than 50% of the length of the database protein were removed.

Illumina-based RNAseq datasets described in 1.6.1 were assembled with an iterative *k*-mer strategy based on velvet [15], parameters: -cov_cutoff 4 -read_trkg yes -exp_cov 100 -min_contig_lgth 150 -max_divergence 0.05 -long_mult_cutoff 0) and used as transcriptional evidences by the EuGene pipeline. Spliced alignments spanning at least 30% of the RNA-seq contig length at a minimum of 97% identity

were retained. The gene-modelling algorithm used the standard EuGene 4.2a parameters, except that non-canonical GC/donor sites were allowed.

Regions spanned either by *Medicago* protein hits or by transcript alignments were preserved from the integrated repeat masking process.

After removal of redundant ncRNA predictions, 38,258 protein-coding genes and 3,129 non protein-coding genes were retained. A large proportion of 5-prime and 3-prime untranslated regions of protein-coding gene models were predicted on the basis of transcript alignments (84% and 83% respectively).

The BUSCO v 3.1.0[16] was run on the set of predicted transcripts. The assessment software detected 97.7% of complete gene models (1,142 complete single copy and 201 duplicated respectively) plus 3 additional fragmented gene models.

Functional annotation of protein-coding genes

Protein coding genes were annotated by integrating five sources of information. Results were successively integrated depending on the expected accuracy of the source of information. Priority was successively given to: i) a BLASTp search of reciprocal best hits with the 1,938 Fabaceae proteins tagged as "reviewed" in the Uniprot database (90% span, 80% identity) as of June 2017 (Uniprot C); ii) 1,523 EC numbers assigned to 10,791 putative enzymes by using the described protocol [17] with BLAST e-value cutoff lowered to $1.e^{-5}$ and pathway-prediction-score set to 0.3 in pathway-tools to increase stringency; iii) the transcription factors and kinases identified (3,284 and 1,358 respectively) by ITAK release 1.7[18] iv) the 4,851 transcription factors identified by PlantTFCat[19] v) the Interpro (release 64.0) search matching 36,298 proteins[20].

The EC numbers were tested against the ENZYME[21] database (November 2017), updated when deprecated and then used to get the description of the enzymes. At each step, the description provided by the source of information was edited when necessary to ensure compliance with a submission to Genbank (Benson DA). Finally, the protein annotations were validated by the tbl2asn software (https://www.ncbi.nlm.nih.gov/genbank/tbl2asn2, November, 2017). A putative function was assigned to 28,019 proteins and 7,625 proteins were tagged as "hypothetical proteins". GO terms were assigned using the BLAST2GO pro software

integrating blastp similarities with Genbank NR (May, 2016, e-value < 1e-5, 20 best-scoring hits) and Interpro release 64 results. The final metrics of the assembly and annotation are described in the Supplementary Table 3.

Evaluation of AMIGA heterogeneity

In order to evaluate the heterogeneity of cv. AMIGA, a bulk of 90 AMIGA plants was resequenced using Illumina HiSeq300, with paired-end 2x150 bp reads at Get-PlaGe core facility (INRA, Toulouse, France). This produced 193,734,276 clean reads corresponding to a total of 64.47x depth.

Cutadapt[22] has been used to remove Illumina Truseq adapter from the sequencing data, and to remove bases with a quality score lower than 30, in both 5' and 3' end of the reads. Reads with a length lower than 35 have been discarded. We used BWA-MEM version 0.7.17[23] to map the resequencing reads to the white lupin reference genome. Picard tools (https://github.com/broadinstitute/picard/issues) have been used to detect and remove PCR and Optical duplicates. We then used GATK 4.0[24] HaplotypeCaller tool to call variants. This identified *ca*. 300,000 SNPs without filtering the data. All the SNPs are evenly distributed on the 25 chromosomes and contigs. We generated a VCF file with this information, available in the white lupin genome browser.

White lupin genome portal

All information regarding white lupin genome sequence is accessible to the scientific community on the white lupin genome portal: www.whitelupin.fr. This website contains a Genome Browser, Expression tools, a Sequence retriever tool as well as all raw data available for download.

Supplementary Note 3. Transposable elements and annotation of repeated sequences

Plant material for cytological analysis

Seeds of *L. albus* cv. AMIGA were germinated on petri dishes with moist paper. After germination root tips were collected. For meiotic tissue, young flower buds were collected from the same accession grown in growth chambers under controlled conditions (16 h light/8 h dark, 25°C day/20°C night, 65% relative humidity and PAR intensity 200 µmol m^{-2} s^{-1}) at B&PMP Institute facilities, Montpellier, France. Pre-treatment and fixation of tissues were performed as described below.

Chromosome preparation for *in situ* hybridization

Chromosome preparations for *in situ* hybridization analysis were conducted as described in [25] with modifications. First, young roots (pre-treated with 8-hydroxyquinoline 2mM for 3-5 h at room temperature) and anthers were fixed in 3:1 (ethanol:acetic acid) for 2-24 h. The fixed tissues were treated with an enzyme mixture (0.7% cellulase R10, 0.7% cellulase, 1.0% pectolyase, and 1.0% cytohelicase in 1× citric buffer) for 1h at 37 °C. Material was then washed twice in water and fragmented in 7 µl of 60% freshly prepared acetic acid into smaller pieces with the help of a needle on a slide. Another 7 µl of 60% acetic acid was added, and the specimen was kept for 2 min at room temperature. Next, a homogenization step was performed with an additional 7 µl 60% acetic acid and the slide was placed on a 55 °C hot plate for 2 min. The material was spread by hovering a needle over the drop without touching the hot slide.

After spreading of cells, the drop was surrounded by 200 µl of ice-cold, freshly prepared 3:1 (ethanol:acetic acid) fixative. More fixative was added and the slide was briefly washed in fixative, then dipped in 60% acetic acid for 10 min and dehydrated in 96% ethanol. The slides were stored until use in 96% ethanol at 4 °C.

Repeat identification and genome-wide repeat annotation

Identification and characterization of moderately to highly repeated genomic sequences was achieved by graph-based clustering of genomic Illumina reads using RepeatExplorer2 pipeline[26]. A total of 1,144,690 of 150bp paired reads, representing

approx. 0.5× genome coverage, were used for the clustering and the 145 largest clusters with genome proportions of at least 0.01% were examined in detail. Clusters containing satellite DNA (satDNA) repeats were identified based on the presence of tandem sub-repeats within their read or assembled contig sequences with TAREAN [27]. Genome-wide TE repeat annotation was performed using the DANTE (Domain-based ANnotation of Transposable Elements) tool[27]. Consensus sequences of satDNA repeats (Supplementary Fig. 2) and rDNA genes were used to perform genome-wide annotation of satDNA and rDNA arrays using the Geneious v. 9.1.8 annotation tool (https://www.geneious.com). The generated GFF3 files were further incorporated on the *L. albus* genome browser.

Probe preparation and fluorescence *in situ* hybridization.

FISH probes were obtained as 5′-Cy3 or 5′-FAM-labeled oligonucleotides (Eurofins MWG Operon, http://www.eurofinsdna.com), or were PCR-amplified as described below. All DNA probes, except oligonucleotides, were labelled with Cy3- or Alexa 488-dUTP (Jena Bioscience) by nick translation, as described by[28] . The sequences of all oligonucleotides and primers are listed in Supplementary Table 4. FISH was performed as described in [25]. Probes were then mixed with the hybridization mixture (50% formamide and 20% dextran sulfate in 2× SSC), dropped onto slides, covered with a cover slip and sealed. After denaturation on a heating plate at 80°C for 3 min, slides were hybridized at 37 °C overnight. Post-hybridization washing was performed in 2× SSC for 20 min at 58°C. After dehydration in an ethanol series, 4′,6–diamidino-2–phenylindole (DAPI) in Vectashield (Vector Laboratories, http://www.vectorlabs.com) was applied. Microscopic images were recorded using a Zeiss Axiovert 200M microscope equipped with a Zeiss AxioCam CCD. Images were analyzed using the ZEN software (Carl Zeiss GmbH).

PCR amplification of tandem repeat and retroelement fragments for probe labeling

Fragments for probe labeling were amplified using gDNA from *L. albus* using the forward and reverse primers as supplied on Supplementary Table 4. Eight PCR reactions for each target repeat were performed in 50 µL reaction volume containing

100 ng of gDNA, 1 µM primers, 1× PCR buffer, 0.2 mM dNTPs, and 1U of Taq polymerase (Qiagen). Thirty-five amplification cycles with proper conditions for each set of primers were run. PCR reactions were sampled, purified and concentrated using Wizard® SV Gel and PCR Clean-Up System (Promega). Correct amplification of PCR fragments was confirmed by Sanger sequencing. After confirmation, the PCR products containing the same class of repeat were collected and used for probe labeling by nick translation as described above.

Identification and generation of LalbCENH3 antibody

The *CENH3* gene was identified *in silico* by BLAST search from the transcriptome data of *L. albus*. The peptide GRVKHTPASLGGPHRRKAKS was used to generate *L. albus* CENH3-specific (*LalbCENH3*) polyclonal antibodies. Peptide synthesis, immunization of rabbits, and peptide affinity purification of antisera were performed by LifeTein (http://www.lifetein.com).

Immunostaining

Immunostaining for CENH3 (Abcam-ab1012) were performed as described before [29] with some modifications. Roots were treated with 8-hydroxyquinoline 2mM for 3-5 h at room temperature and fixed in ice-cold 4% paraformaldehyde in 1×PBS buffer pH 7.5 (1.3 M NaCl, 70 mM Na_2HPO_4, 30 mM NaH_2PO_4) for 1 h and squashed in a drop of the same buffer. Then, the slides were washed in 1×PBS and blocked with 3% BSA for 30 min at 37 °C. The antibodies used were rabbit anti-LalbCENH3 diluted 1:500 in 1% BSA in 1xPBS. The detection of anti-LalbCENH3 and anti-H3K4me3 were done with goat anti-rabbit CF488 (Sigma, SAB4600389), or alternatively goat anti-rabbit Alexa-594 (JacksonImmunoResearch-111-585-008), and donkey anti-mouse Fluorescein (JacksonImmunoResearch-715-097-003), respectively, diluted 1:200 in 1×PBS containing 1% BSA. The slides were counterstained with 2 µg/ml 4',6-diamidino-2-phenylindole (DAPI) in Vectashield H-1000.

Super-resolution microscopy

To analyze the structures and spatial arrangement of FISH signals and the global chromatin at a lateral optical resolution of ~120 nm (super-resolution, achieved with a 488 nm laser), 3D structured illumination microscopy (3D-SIM) was applied using a C-Apo 63×/1.2W Korr objective of an Elyra PS.1 microscope system and the software ZEN (Carl Zeiss GmbH). Image stacks were captured separately for each fluorochrome using the 561, 488, and 405 nm laser lines for excitation and appropriate emission filters, and then merged using the ZEN software[30] .

CENH3-ChIP and ChIP-seq analysis

Chromatin immunoprecipitation experiments were done with Abcam ChIP Kit - Plants (ab117137) following the manufacturer's instructions. First, 1 g of young *L. albus* cv. AMIGA leaves were collected and cross-linked with formaldehyde 1% for 15 min on ice. Leaves were then ground in liquid nitrogen and sonicated using a Diagenode Sonicator. Sonicated chromatin-DNA ranging from 200-1000 bp was immunoprecipitated using anti-LalbCENH3. Immunoprecipitated DNA and, as control, Input chromatin DNA samples (3-7ng for each sample) were sent for ChIPseq at BGI. The original ChIPseq sample data are available at White Lupin Genome Website (http://www.whitelupin.fr)

To identify repeats associated with CENH3-containing chromatin, reads from the ChIPseq experiment obtained by sequencing DNA from isolated chromatin prior to (the input control sample) and after immunoprecipitation with the CENH3 antibody (the ChIP sample) were separately mapped to the repeat clusters. The mapping was based on read similarities to contigs representing individual clusters, using BLASTn (22) with parameters "-m 8 -b 1 -e 1e-20 -W 9 -r 2 -q -3 -G 5 -E 2 -F F" and custom Perl scripts for parsing the results. Each read was mapped to a maximum of one cluster, based on its best similarity detected among the contigs. Ratio of ChIP/input reads assigned to individual clusters was then used to identify repeats enriched in the ChIP sample as compared to the input.

Tandem repeat-rich regions create a technical challenge to genome assembly [31]. By means of *in silico* analysis with RepeatExplorer2, we identified 14 tandem repeat clusters (CL1, CL2, CL10, CL21, CL52, CL53, CL55, CL68, CL77, CL85,

CL114, CL118, CL121, CL127) accounting for ~15% of the genome (Supplementary Table 5 & 6; Fig. 1a). These tandem-repeats varied from short monomer length with 5 bp consensus sequence (CL2) to very long monomers up to 918bp (CL121). After further characterization of the consensus sequence of the identified tandem repeats we observed that CL52 and CL127 are very similar to CL1 and was grouped within a single supercluster, thus representing a subset of the same repeat comprising the most abundant tandem repeat family of WL. CL10 (78bp) and CL21 (38bp) are similar satellites comprising different subfamilies. CL10 represents a dimer organization of CL21. CL118 (182bp) also showed sequence similarity to CL10 and CL21. Among the identified tandem repeats CL1-CL52-CL127 (170bp) and CL2 (5bp) were highly abundant comprising both together ~12% of the genome (Supplementary Table 5). Further characterization of satellite sequences is provided on Supplementary Table 6.

Supplementary Note 4. White lupin diversity analysis

We selected 14 white lupin accessions to evaluate a broader range of the genetic diversity and determine population structure and linkage disequilibrium. The 14 accessions selected for this work, together with the accession AMIGA - used to construct the white lupin reference genome - are described in the Supplementary Table 7. This group of accession comprises cultivated accessions with different pattern of sowing, one landrace and two wild accessions.

Data generation with short-reads technology

Young leaves of 30 plants were used to extract genomic DNA of each accession using the QIAGEN Genomic-tip 100/G kit following the supplier's recommendations. The accessions were sequenced using Illumina technology using paired-end 2x150 bp short-reads. It was generated a total of 310.95 Gb of data with average sequencing depth of 45.99x (Supplementary Table 8).

Mapping and SNP detection

Cutadapt[22] has been used to remove Illumina Truseq adapter from the sequencing data, and to remove bases with a quality score lower than 30, in both 5' and 3' end of the reads. Reads with a length lower than 35 have been discarded. We then used BWA-MEM version 0.7.17[23] to map the resequencing reads from all 15 genotypes to the white lupin reference genome. PCR and Optical duplicates have been detected and removed using Picard Tools. After that, GATK 4 HaplotypeCaller tool have been used in emit-ref-confidence GVCF mode to produce one gvcf file per sample. These files have been merged using GATK CombineGVCFs. Finaly, GATK GenotypeGVCFs have been used to produce a vcf file containing variants from all the 15 samples. This identified a total of 6,620,353 SNPs/indel. After filtering for minimum allele frequency of 0.15 and heterozygosity frequency of 0–0.2, 2,659,837 SNPs were retained to further analysis. All the SNPs are evenly distributed on the 25 chromosomes with a mean of SNP density of 10.74 SNPs per Kb of sequence (Fig. 2A, Supplementary Table 9).

Phylogenetic analysis, population structure and linkage disequilibrium

The genetic distance matrix was calculated based on identity-by-state similarity method and an average cladogram constructed using neighbor-joining algorithm implemented on TASSEL 5.2.51[32]. Then, a phylogenetic tree was prepared using the iTOL v 4.3 [33]. A principal component analysis (PCA) was also performed in R [34] (http://www.R-project.org/) function “prcomp” .

A Bayesian model-based clustering method implemented with STRUCTURE v2.3.4 [35,36] was used to investigate the population structure using all the filtered SNPs. The program was run 10 times for each K value, ranging from 1 to 5, with a 1,000 burn-in time and 1,000 iterations. The optimal K value was determined based on the ΔK from the Structure Harvester v0.6.94 [37] program, through Evanno’s test [38].

Linkage Disequilibrium (LD) was estimated with the r^2 statistics using the R package snpStats [39] (version 1.32.0) using a subset comprising 46,783 SNPs, distributed evenly on all chromosomes (average of 1871 SNPs per chromosome). Heatmaps of pairwise LD between markers were plotted using the R package LDheatmap (version 0.99-5) [40].

Long-read sequencing and de novo assembly of GRAECUS and P27174

We selected the wild accession GRAECUS and the landrace P27174 to a further investigation of the impact of domestication on the white lupin genome. Genomic DNA of both accessions was extracted as described on the section 3.1. Long-read sequencing was realized using Oxford Nanopore technology, using a GridION 18.04.1-0, with a software Minknow 1.10.24-1 at platform at Get-PlaGe core facility (INRA, Toulouse, France). Briefly, high MW DNA was used to prepare a library with the ONT with the Ligation Sequencing Kit 1D (sqk-lsk109). The DNA was sequenced using a single ONT MinION R9.4 flowcell (FLO-MIN106) for 48h. Base-calling was performed using Albacore 2.1.10-1. This produced 1,280,206 sequences for GRAECUS, corresponding to 12.45 Gb of data with a N50 length of 13.6 Kb (27.6 x of sequencing depth). For the accession P27174 this produced a total of 1,738,579 reads corresponding to 14.59 Gb of data with N50 length of 11.8 Kb (32.36 x of sequencing depth).

The *de novo* assembly of the two genotypes were performed using CANU[1]. For P27174, two rounds of correction have been made prior to the assembly step, using the parameters correctedErrorRate=0.16 and corMaxEvidenceErate=0.15. For GRAECUS, only one round of correction has been made, using minOverlapLength=400, correctedErrorRate=0.16 and corMaxEvidenceErate=0.15. The Illumina paired-end data described in 3.1 were used to polish two times the two genome assemblies using Pilon[8]. BUSCO v 3.0.0[16] was run on the set of predicted transcripts. The assessment software detected for GRAECUS 96.8% of complete gene models (1,131 complete single copy and 200 duplicated respectively) plus 9 additional fragmented gene models. For P27174 97.8% of complete gene models (1,125 complete single copy and 220 duplicated respectively) plus 4 additional fragmented gene models. Structural variation of these two accession were performed in Assemblytics software [41] based on whole genomes alignments generated with MUMmer [42]. The final assembly of GRAECUS has 428.06 Mb and P27174 458.23 Mb (Supplementary Table 10).

Supplementary Note 5. Genome comparison and evolution

Evolutionary analysis of legume genomes

The proposed evolutionary scenario was obtained following the method described in Pont et al. [43] based on synteny relationships identified between *L. albus* ('Lal' current manuscript), *L. angustifolius* [44] ('Lan'), *Pisum sativum* ('Ps', [45]), diploid peanut ('Ad', *Arachis duranensis*, [46]), Lotus ('Lj', *Lotus japonicus*, [47]), barrel medic ('Mt', *M. truncatula*, [48]), chickpea ('Ca', *Cicer arietinum*, [49]), pigeon pea ('Cc', *Cajanus cajan*, [50]), soybean ('Gm', *Glycine max*, [51]), common bean ('Pv', *Phaseolus vulgaris*, [52]), mungbean ('Vr', *Vigna radiata*, [53]), and adzuki bean ('Va', *Vigna angularis*, [54]). Genomes were aligned (BlastP of annotated proteins) to define conserved gene pairs based on alignment parameters (CIP for Cumulative Identity Percentage of 50% and CALP for Cumulative Alignment Length Percentage of 50%,[55]) using *Medicago truncatula* and *Phaseolus vulgaris* as pivots for respectively the Galegoid and Millettioid subfamilies. Groups of conserved genes were clustered into synteny blocks (*i.e.* conserved genes on the same chromosomal combination pair excluding blocks with less than 5 genes, see Table S4) defining independent sets of blocks sharing orthologous relationships in modern species. Then, conserved groups of gene-to-gene adjacencies defining identical chromosome-to-chromosome relationships between all the extant genomes were merged into conserved ancestral regions (CARs). Alternatively, CARs are defined from synteny blocks not conserved between all the investigated legume genomes but shared between representatives of the Genistoid, Galegoid and Millettioid subfamilies. The ancestral karyotype is a 'median' or 'intermediate' genome consisting of proto-chromosomes (CARs) defining a clean reference gene content, common to the extant species investigated. From the reconstructed ancestral karyotype an evolutionary scenario was then inferred taking into account the fewest number of genomic rearrangements which may have occurred between the inferred ancestors and the modern genomes. Comparative genomics data described here are made available in the public web tool https://urgi.versailles.inra.fr/synteny/legumes (Raphael Flores, INRA-UNRGI, Université Paris-Saclay, 78026, Versailles, France).

Genome synteny, intragenomic collinearity and gene family identification

To identify intragenomic collinearity blocks inside the white lupin genome we used SynMap (CoGe, www.genomevolution.org) using homologous CDS pairs using the following parameters: Maximum distance between two matches (-D): 20; Minimum number of aligned pairs (-A): 10; Algorithm “Quota Align Merge” with Maximum distance between two blocks (-Dm): 500.

Chromosome collinearity between white lupin and narrow-leafed lupin (*L. angustifolius,* v1.0) and *M. truncatula* (Mt4.0) was carried out with MCscan (phyton version) [56,57]. The following parameters were used to detect syntenic blocks: alignment similarity e $\leq 10^{-05}$; average intergenic distance (u) = 40; number of genes required to call synteny, (s) = 5; gap penalty (g) = 2.

We used a comparative analysis to examine the conservation of gene repertoires among orthologs in the genomes of white lupin, narrow-leafed lupin (v1.0) *M. truncatula* (Mt4.0) and *Arabidopsis thaliana* (TAIR10). First, we aligned all-to-all proteins using BLASTP (e-value of $1e^{-5}$). Genes were then clustered using OrthoMCL (1.4) implemented in OrthoVenn [58] with a Markov inflation index of 1.5 and a minimum e-value of $1e^{-15}$.

Supplementary Note 6. White lupin symbiosis

In silico identification of white lupin symbiotic genes

Symbiotic genes were searched in 96 plant genomes covering different Angiosperm orders and symbiotic abilities (mycorrhization and/or root nodule symbiosis, no symbiosis) as indicated in Supplementary Table 7. Reference proteins previously identified in *M. truncatula* were used as queries for tBLASTn 2.7.1+ [59] and hits with a e-value < 1e-10 were selected for each investigated plant species. Selected sequences were aligned using MAFFT v7.380 [60] with default parameters. When needed, alignments were cleaned using GBlocks [61] implemented in SeaView v4.7 [62]. Alignments were then subjected to phylogenetic analysis using IQ-TREE v1.6.7 [63]. First, the best-fitting evolutionary model was determined for each alignment using ModelFinder [64] before performing maximum likelihood analysis. Branch supports were tested using 10,000 replicates of UltraFast Bootstraps [65]. Tree visualization was performed through the iTOL v4.2.3 platform [33].

Molecular cloning and hairy-root transformation of white lupin

The primers for *pLaSYMRK*(Lalb_Chr09g0325781; F-5'-TCGGGTATAAGCAAGCCAAC-3'; R-5'- CTATTTCTGCATCCTTGCTAAGC -3') and *pLaCCamK* (Lalb_Chr19g0132601; F-5'- GGTGTGTGTTAGATAGATGCCTTTG -3'; R-5'-GATGGCTACATGGATTTTCTCAC-3') were designed using Primer3Plus [66]. They were used to amplify a total of 1 963 and 2 185 bp upstream of the start codon of *LaSYMRK* and *LaCCamK*, respectively, from white lupin genomic DNA with the addition of the attb1 (5'-ACAAGTTTGTACAAAAAAGCAGGCT-3') and attb2 (5'-ACCACTTTGTACAAGAAAGCTGGGT-3') adapters. Amplified fragments were subsequently cloned into the pDONR221 by Gateway reaction (Thermo Fisher). The promoters were then cloned into the binary plasmid pKGW-FS7[67] containing a green fluorescent protein-glucuronidase (GFP-GUS) fusion by Gateway cloning. Hairy root transformation of white lupin plants as well as histochemical and microscopic analysis were performed as described before [68].

White lupin symbiotic genes

The presence of genes known to be specifically required for arbuscular mycorrhizal symbiosis (AMS), root nodule symbiosis (RNS) or both associations was investigated (Supplementary Table 8). Phylogenetic analyses with seven other angiosperm genomes covering the diversity of symbiotic abilities [69,70] (revealed that RNS specific genes (such as NFP, EPR3, NIN, RPG, PUB1) along with gene required for both symbiosis (such as SYMRK, CYCLOPS, CCamK, VAPYRIN…) are retained in *Lupinus albus* and are expressed (Supplementary Fig. 10). By contrast, AMS specific genes (PT4, RAM1, RAM2, FatM, HA1, STR1, STR2 and Exo70) were not retrieved in the *Lupinus albus* genome. This result expands previous findings on the draft genome *of Lupinus angustifolius* and supports the hypothesis that loss of AMS occurred early during the evolution of the *Lupinus* genus or their genistoid progenitors.

Supplementary Note 7. Candidate genes controlling cluster root formation

Spatial transcriptome for mRNA and small RNA

Seeds of white lupin (*L. albus* L. cv. AMIGA) were germinated for 4 days on vermiculite soaked with water. Thereafter, the seedlings were transferred to an aerated nutrient solution in the absence of phosphate in growth chambers under controlled conditions (16h light/8h dark, 25°C day/20°C night, 65% relative humidity and PAR intensity 200 µmol.m^{-2}.s^{-1}).

After 12 days of culture, ten cluster roots coming from four grown plants were harvested and dissected in eight parts of 0.5-cm from the apex of the lateral root that carries the cluster root. As control, 1-cm of lateral roots without cluster roots, sampled 1-cm away from the primary root, were collected. Four biological replications were produced for each experiment.

Total RNA was extracted from all frozen samples using the Direct-zol RNA MiniPrep kit (Zymo Research, Irvine, CA) according to the manufacturer's recommendations.

For mRNA sequencing, 36 independent root RNA-seq libraries were constructed and sequenced at Get-PlaGe core facility (INRA, Toulouse, France). The Illumina TruSeq Stranded mRNA Sample Preparation Kit (Illumina Inc.) was used according to the manufacturer's protocol. The samples were sequenced using Paired-end sequencing was performed generating pair-ended 2 x 150 bp reads using TruSeq SBS kit v3 sequencing chemistry (Illumina Inc.) in one lane of Illumina NovaSeq instrument according to the manufacturer's instructions. A total of 2,048,118,650 paired-end reads of 150 pb were sequenced using an Illumina NovaSeq6000 Sequencer. To remove low quality sequences, the RNA-seq reads were checked and trimmed with a minimum quality score of 30 in both 3' and 5' end. The resulting reads shorter than 35 pb have been discarded. The quality checked RNA-seq reads were then mapped on white lupin reference genome using Hisat2 software. Transcripts were assembled and quantified using Stringtie software. Gene counts were extracted and imported in the R package DESeq2. These counts have been normalized according to the size factor computed by DESeq2.

For small RNA sequencing, 24 independent root RNA-seq libraries were constructed and sequenced at Transcriptomic Platform IPS2 (IPS2, Paris, France).

The NEXTflex™ Small RNA-Seq kit was used for generation of small RNA-seq libraries according to the manufacturer's protocol. All small RNA libraries were sequenced on an Illumina NextSeq 500 sequencing platform, using a single-end, 75 nt read metric instrument according to the manufacturer's instructions. A total of 460,506,072 reads of 75 nt were sequenced. Small RNA-seq reads were trimmed using cutadapt version 1.11 [22] to remove remnants of the following 3'-adapter sequence. Only sequences that had the adapter were kept, ensuring that they are small fragments. The adapter trimming was performed based on the adapter sequence: TGGAATTCTCGGGTGCCAAGGAACTCCAGTCAC of 33 nucleotides long, removing the adapter if the length of the match is at least 10 nucleotides. A second round of trimming was performed with cutadapt to remove the four nucleotides on the 5' and 3' ends of the reads (which corresponded to the four degenerated nucleotides on each "High Definition" (HD) adapter). Empty reads (adapter dimer) and all the sequences shorter than 15 nucleotides were discarded. A total of 424,385,998 trimmed reads were kept for final analysis. The sequence files produced were collapsed to include only non-redundant reads and the number of occurrences of each. To quantify the level of expression of Lupin miRNAs we used the high conservation in sequence of the main miRNA families among plant species. We therefore use the publicly available mature miRNA of three well studied species in the Fabaceae clade max present in miRBase v22[71]: *Medicago truncatula, Lotus japonicas* or *Glycine max*. Only sequences corresponding exactly to the published mature miRNA sequence were retained and counted.

AMIGA and GRAECUS root sampling and expression analysis of cluster root initiation genes

We sampled 2-3 cm of lateral roots 1 cm away from the primary root in the top 5 cm (cluster root region, CRR) and at 10 cm from the top (regular lateral root region, NLR) of the root system of AMIGA and GRAECUS plants 9 days after germination. Three CRR and 3 NLR independent samples were collected for each accession. Total RNA from these samples was extracted using the Direct-zol RNA MiniPrep kit (Zymo Research, Irvine, CA) according to the manufacturer's recommendations. RNA concentration was measured on a NanoDrop (ND1000) spectrophotometer. Poly(dT) cDNA were prepared from 2 µg total RNA using the revertaid First Strand cDNA Synthesis (Thermo Fisher). Gene expression was measured by quantitative

Real Time - Polymerase Chain Reaction (qRT-PCR) (LightCycler 480, Roche Diagnostics, Basel, Switzerland) using the SYBR Premix Ex Taq (Tli RNaseH, Takara, Clontech, Mountain View, CA) in 384-well plates (Dutscher, Brumath, France). Target quantifications were performed with specific primer pairs designed using Universal Probe Library software (Roche Diagnostics, Basel, Switzerland). The primer pairs used in the parallel reactions are described on the Supplementary Table 11. Expression levels were normalized to *LaHelicase* (Lalb_Chr13g0304501). All qRT-PCR experiments were performed in technical quadruplicates. Relative gene expression levels were calculated according to the ΔΔCt method[72], using as a calibrator the NLR samples. All experiments were performed as three biological replicates.

To check the quality among the experimental replicates, we verified the clustering of the samples in a principal component analysis (PCA), Supplementary Figure 11. The first two principal components are explaining 79% of the total variance among the samples. The PCA plot of the samples in the 2D plane spanned by their first two principal components shows that they are grouped by root region (LR-S7).

Supplementary Note 8. Identification and characterization of seed storage protein

White lupin protein extracts and electrophoresis

Total soluble proteins were extracted from mature seeds of AMIGA, P27174 and GRAECUS using 500 µl of the Tris-HCl/SDS lysis buffer for 10 mg of seed powder (two independent extractions, A and B, realized). For AMIGA, seeds were split in two samples based on seed color (dark brown, DB; light brown, LB). The protein extracts were stirred for 30 min at room temperature and then centrifuged (35,000g) for 10 min. The supernatant was subjected to a second clarifying centrifugation step as above. Protein concentration was determined using the Protein assay from Bio-Rad (Marnes-la-Coquette, France). For each seed sample, 10 µg proteins were separated by one-dimensional electrophoresis (1D) in a SDS polyacrylamide gel (SDS-PAGE) using the XCell4 *Surelock*^{TM} Midi-Cell system (Life Technology) [Resolution gel: 4.1 mM Tris-HCl pH 8.8, 40% (v/v) acrylamide/bisacrylamide (30%/0.8%), 1% (g/v) SDS, 0.05% (g/v) ammonium persulfate, 0.05% (v/v) TEMED; Concentration gel: 0.6 mM Tris-HCl pH 6.8, 13% (v/v) acrylamide/bisacrylamide (30%/0.8%), 1% SDS, 0.05% ammonium persulfate, 0.05% TEMED; Electrophoresis buffer: 50 mM Trizma base, 380 mM glycine, 0.1% (g/v) SDS]. Gels were stained with Coomassie Blue R250 (Bio-Rad) and scanned using an Odyssey Infrared Imaging System (LI COR BIOSCIENCES GMBH, Bad Homburg, Germany), with an intensity of 7.5 and a resolution of 84 µm. Protein band detection and quantification were performed using Phoretix 1D (v11.2, Total Lab Limited, Newcastle, UK). The quantitative data of each band was normalized by dividing its volume by total band volume in the well, and the molecular weight (kDa) of each band was calculated using a low-range protein ladder (Bio-Rad).

Mass spectrometry analysis

Gel pieces were washed in water: acetonitrile solution (1:1, 5 min) followed by 100% acetonitrile (10 min). Reduction and cysteine alkylation were performed by successive incubation with 10 mM dithiothreitol in 50 mM NH_4HCO_3 (30 min, 56 °C), then 55 mM iodoacetamide in 50 mM NH_4HCO_3 (20 min, RT, in dark). Pieces were then incubated with 50 mM NH4HCO3 and acetonitrile (1:1, 10 min) followed by acetonitrile (15 min). Proteolytic digestion was carried out overnight using 25 mM

NH_4HCO_3 with 12.5 ng/µl Trypsin (Sequencing grade, Roche diagnostics, Paris, France). Resultant peptides were extracted by incubation in 5% formic acid (sonicated) with the supernatant removed and saved, followed by incubation in acetonitrile and 1% formic acid (1:1, 10 min) and a final incubation with acetonitrile (5 min), again supernatant was removed and saved. These two peptide extractions were pooled and dried using a SPD1010 speedvac system (Thermosavant, Thermofisher Scientific, Bremen, Germany) and the resultant peptide mixture was analyzed by nanoflow liquid chromatography tandem mass spectrometry (Nano LC-MS/MS).

All experiments were performed on a dual linear ion trap Fourier Transform Mass Spectrometer (FT-MS) LTQ Orbitrap Velos (Thermo Fisher Scientific, Bremen, Germany) coupled to an Ultimate® 3000 RSLC Ultra High Pressure Liquid Chromatographer (Dionex, Amsterdam, The Netherlands) controlled by Chromeleon Software (version 6.8 SR11; Dionex, Amsterdam, The Netherlands).

Samples were desalted and concentrated for 10 min at 5µL/min on an LCPackings trap column (Acclaim PepMap 100 C18, 75 µm inner diameter x 2cm long, 3µm particles, 100Å pores).

The peptide separation was conducted using a LCPackings nano-column (Acclaim PepMap C18, 75µm inner diameter x 50cm long, 2µm particles, 100Å pores) at 300nL/min.

Gradient profile:

- Columns equilibration with 96% solvent A (0.1% formic acid, 97.9 % water, 2% acetonitrile (v/v/v)) and 4% solvent B (0.1% formic acid, 15.9 % water, 84% acetonitrile (v/v/v)).
- Gradient of 4-60% solvent B for 90min.
- Stage at 99% solvent B for 10min.

Data were acquired in positive mode in data-dependent mode to automatically switch between high resolution full-scan MS spectra (R 60 000) collected in profile mode and low-resolution CID-MS/MS in centroid mode (m/z 300-1800). The 20 most intense peptide ions with charge states ≥ 2 were sequentially isolated and fragmented in the high-pressure linear ion trap by low energy CID (collision energy 35%, activation time 10ms, Qz 0.25). Dynamic exclusion is activated during 30 seconds with a repeat count of 1.

For protein identification, MS/MS ion searches were performed using Mascot search engine version 2.3.2 (Matrix Science, London, UK) via Proteome Discoverer 2.1 software (ThermoFisher Scientific, Bremen, Germany) against *L. albus*_protein database and NCBI prot_viridiplantae. The search parameters included trypsin as a protease with two allowed missed cleavages and carbamidomethylcysteine, methionine oxidation and acetylation of N-term protein as variable modifications. The tolerance of the ions was set to 5 ppm for parent and 0.8 Da for-fragment ion matches. Peptides and proteins identified by MASCOT were validated using « Peptid Prophet » and « Protein Prophet » algorithm with Scaffold software (version 4.8.7, Proteome Software, Portland, USA). Protein identifications were accepted if they contained at least two identified peptides.

Identification of conglutin protein genes in the *L. albus* genome and phylogenetic relationship

Conglutin cDNA sequences of *L. angustifolius* described previously [73] were used to identify orthologs on *L. albus* genome using BLAST tool search. The sequences used as query were: Lang_ALPHA1 [Genbank:HQ670406], Lang_ALPHA2 [Genbank:HQ670407], Lang_ALPHA3 [Genbank:HQ670408], Lang_BETA1 [Genbank:HQ670409], Lang_BETA2 [Genbank:HQ670410], Lang_BETA3 [Genbank: HQ670411], Lang_BETA4 [Genbank:HQ670412], Lang_BETA5 [Genbank:HQ670413], Lang_BETA6 [Genbank:HQ670414], Lang_BETA7 [Genbank:HQ670415], Lang_GAMMA1 [Genbank: HQ670416], Lang_GAMMA2 [Genbank:HQ670417], Lang_DELTA1 [Genbank: HQ670418], Lang_DELTA2 [Genbank:HQ670419], Lang_DELTA3 [Genbank:HQ670420] and Lang_DELTA4 [Genbank:HQ670421].

A phylogenetic tree with all the *L. angustifolius* conglutin aminoacid sequences and the amino acid ortholog sequences identified on *L. albus* genome were constructed using the pipeline on the Phylogeny.fr platform [74]. Sequences were aligned with MUSCLE (v3.7) conFig.d for highest accuracy (MUSCLE with default settings). The phylogenetic tree was reconstructed using the maximum likelihood method implemented in the PhyML program (v3.0). The WAG substitution model was selected assuming an estimated proportion of invariant sites and 4 gamma-distributed rate categories to account for rate heterogeneity across sites. The gamma

shape parameter was estimated directly from the data. Reliability for internal branch was assessed using the aLRT test (SH-Like).

L. albus conglutin identification

We identified a total of 15 genes coding for conglutin proteins in the white lupin genome (Supplementary Table 13), a similar number (16) to the one described for the narrow-leafed lupin genome [73]. We identified 3 distinct α-conglutin – also known as 11S globulin – orthologs one for each narrow-leafed lupin α-conglutin. These proteins were already described in the literature [73], and in this work we could localize the genes coding for each one of them that were positioned on the chromosomes 02, 21 and 25 (Supplementary Fig. 21 and Supplementary Data 13). We identified 6 genes that are orthologs of β-conglutin (or 7S globulin), one less than found in *L. angustifolius*. One of them is located on chromosome 22 and the other five are positioned on chromosome 6. The β-conglutin genes on chromosome 6 are tandem duplications and form a cluster of 87.5 kb positioned from 6,684,640 bp to 6,772,112 bp. They showed highest homology among themselves than to β-conglutins of narrow-leafed lupin (Supplementary Figure 3). The same pattern was shown for the 4 δ-conglutin identified (Supplementary Table 13): they form a paralogs cluster on chromosome 24 and with a high conservation level. We also identified 2 basic 7S proteins, that are specific proteins for lupin seeds [75], that have a high level of homology with the 2 *L. angustifolius* γ-globulin (Supplementary Figure 3). They are single genes on chromosomes 4 and 15, respectively.

L. albus seed storage protein characterization

We analyzed seed protein composition of the three white lupin accessions explored in this study (domesticated, landrace, and wild accessions, Fig. 6a-c). The seed protein profile of GRAECUS was highlight contrasted to that of the other accessions. Five protein bands showing striking differences in their abundance between the three accessions were selected for mass spectrometry analyses (Fig. 6b, red arrows). With the availability of the full-length sequences of white lupin, the most abundant proteins in these bands were identified as corresponding to β-conglutins from the gene cluster on chromosome 6 (Supplementary Table 14).

It was previously described that β-conglutins are the major component of lupin seed storage protein and account for about 43.4% [75], followed by α-conglutins (33%). β-conglutins are relevant in human nutrition since they have been identified as being hypocholesterolemic [76] and preventing or reducing hypertension [77]. According to Duranti et al. [75], β-conglutins have pronounced structural variation, being composed of over 20 polypeptide chains with no disulfide bridges, with a broad range of molecular masses (M_r 15–65 kDa). This revealed that domestication was associated with the disappearance of high molecular weight storage proteins (~60 kDa, Extended Fig. 6b-c). These corresponded to ß-conglutin precursors that can be proteolytically processed during seed development to give rise to polypeptides of lower molecular weights. The accumulation of these processed forms at the expense of precursor forms in seeds of the domesticated accession AMIGA could be linked to an increased germination capacity. Importantly, the unprocessed ß-conglutins of high molecular weight have high allergenicity[78], a trait that might have been counter-selected during domestication.

Supplementary Note 9. Alkaloid characterization

Tissue sampling for alkaloid analysis

Seeds of WL accessions. AMIGA, GRAECUS and P27174, were germinated on germination paper over 4 days. Seedlings were cultivated in growth chambers under controlled conditions (16 h light – from 8:00 to 23:59 / 8 h dark – from 00:00 to 7:59, 25°C day/20°C night, 65% relative humidity and PAR intensity 200 µmol m^{-2} s^{-1}). After germination, seedlings were transferred to 200-L tanks. The hydroponic solution was modified from previously described medium [11] without phosphate, according to the following composition: $MgSO_4$, 54 µM; $Ca(NO_3)_2$ 400 µM; K_2SO_4 200 µM; Na-Fe-EDTA 10 µM; H_3BO_3 2.4 µM; $MnSO_4$ 0.24 µM; $ZnSO_4$ 0.1 µM; $CuSO_4$ 0.018 µM and Na_2MoO_4 0.03 µM. The nutrient solution was continuously aerated and was renewed every 7 days.

At the 3-leaf stage, young leaf material was collected from each plant at 11:00 h. At 5-leaf stage young leaf, stem, root system and the rest of the shoot material were also collected at 11:00 h. All tissues were placed immediately in liquid nitrogen and stored at −50 °C until further analyses. Each plant represents a biological replicate. There were 4 biological replicates for each genotype.

Alkaloid quantification

Frozen tissue samples were pulverized in liquid nitrogen using metal balls and a ball grinder. Around 50 mg of frozen powder were mixed with 500 µl of 60% methanol containing 0.06% formic acid and 5 mg/l caffeine as internal standard. The mixtures were agitated vigorously for 2 h at room temperature. Following a brief centrifugation to separate the solids, the extracts were diluted 1:5 (v:v) with water, cleared through a 0.22-µm filter, and analyzed by reversed-phase LC-MS.

The diluted, cleared extracts were analyzed using a Thermo Fisher Dionex UltiMate 3000 RS HPLC/UHPLC system coupled to a Bruker compact QqTOF mass spectrometer via an ESI source. Analyte separations were performed on a Kinetex XB-C18 column (100 x 2.1 mm, 1.7 µm, 100Å, Phenomenex). The eluent flow rate was 0.3 ml/min and the column temperature was kept constant at 40°C. Mobile phases A and B consisted of 0.05% formic acid in water and 0.05% formic acid in acetonitrile, respectively. The elution profile was 0 – 1 min, 2% B (constant); 1 – 16 min, 2 – 25% B (linear); 16 – 24 min, 25 – 65% B (linear), 24 – 26 min, 65 – 100% B

(linear); 26 – 27 min, 100% B (constant); 27 – 27.5 min, 100 – 2 % B (linear); and 27.5 – 33 min, 2% B (constant). ESI mass spectra were acquired in positive ionization mode with the following parameters: capillary voltage of 4500 V; end plate offset of -500 V; source temperature of 250°C; desolvation gas flow of 8.0 l/min; and nebulizer pressure of 2.5 bar. N_2 was used as desolvation, nebulizer and collision cell gas.

The identity of lupanine was confirmed by comparison with a standard (Sigma-Aldrich), while that of the other alkaloids was inferred from their exact masses and their ESI(+) fragmentations at different collision energies. For quantification, alkaloid peaks were normalized to the signal of the internal standard and the fresh weight of the samples.

Expression analysis

Total RNA from leaves harvested on 3-leaf and 5-leaf stage plants was extracted using the Direct-zol RNA MiniPrep kit (Zymo Research, Irvine, CA) according to the manufacturer's recommendations. RNA concentration was measured on a NanoDrop (ND1000) spectrophotometer. Poly(dT) cDNA were prepared from 1.5 µg total RNA using the revertaid First Strand cDNA Synthesis (Thermo Fisher). Gene expression was measured by quantitative Real Time - Polymerase Chain Reaction (qRT-PCR) (LightCycler 480, Roche Diagnostics, Basel, Switzerland) using the SYBR Premix Ex Taq (Tli RNaseH, Takara, Clontech, Mountain View, CA) in 384-well plates (Dutscher, Brumath, France). Target quantifications were performed with specific primer pairs designed using Universal Probe Library software (Roche Diagnostics, Basel, Switzerland). The primers pairs used in the parallel PCR reaction are described in the Supplementary Table 15. Expression level was normalized to LaEIF-4 (Lalb_Chr07g0195211). All qRT-PCR experiments were performed in two technical replicates as well as a negative control were included in each assay and the values presented represent means ± SD. Relative gene expression levels were calculated according to the ΔΔCt method [72]. All experiments were performed as four biological replicates.

Delineating the *pauper* region

Wild type and landraces of white lupin all contain high levels of quinolizidine alkaloids while modern cultivars incorporate one or more mutations conferring low alkaloid content [79] . The most commonly used mutation is *pauper*, a single gene locus that was mapped to linkage group 18[10]. We re-examined marker segregation data from [10] and, taking in account the small degree of marker order ambiguity present in the data, we identified a 6.2 cM map interval flanked by markers TP1572 and TP70046 that we conservatively predicted must contain the *pauper* locus. Sequence tags for TP1572 and TP70046 were aligned to the AMIGA reference genome and physical coordinates defined.

Identification of genes on the *pauper* region

Measurement of the abundance of key alkaloids in various organs confirmed that the cultivated accession AMIGA contains low levels of lupanine, 13-hydroxylupanine and 13-tigloyloxylupanine (Fig. 6d). We were not able to match this lower amount of alkaloids with significantly a reduced expression of the known genes coding for enzymes involved in the biosynthetic pathway (Extended Fig. 6e). Next, we explored the gene content of the *pauper* region on Chr18 that is responsible for the low-alkaloid trait in most modern WL accession, including AMIGA [10,80,81]. Based on a reappraisal of mapping data presented by [10], we predicted conservatively that pauper is contained within a 958 Kb region containing 66 genes (Supplementary Table 16), including genes coding for transcription factors, transporters, and enzymes. Functional characterization of these genes will lead to a better understanding of the alkaloid content reduction observed in modern accessions and provide the genetic mechanisms underlying the *pauper* locus.

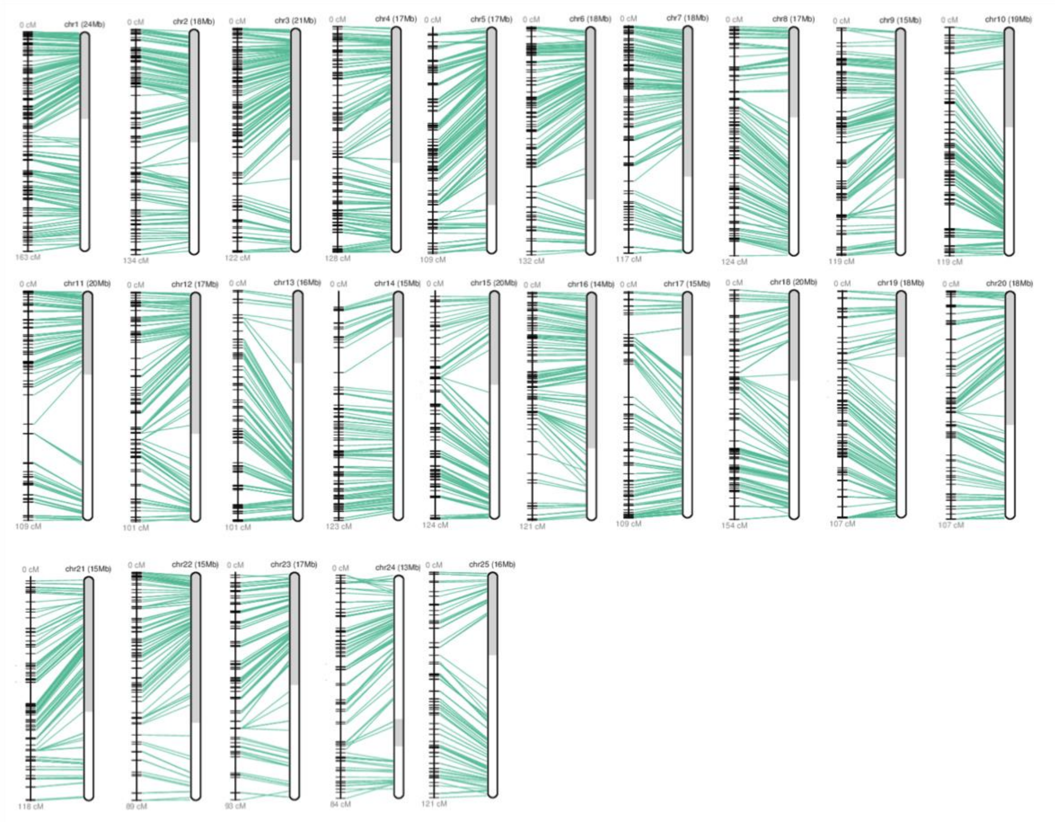

Supplementary Figure 1. Integration of sequence data and genetic map as provided by ALLMAPS. Each chromosome is represented by its linkage group (right) and chromosome arms (left).

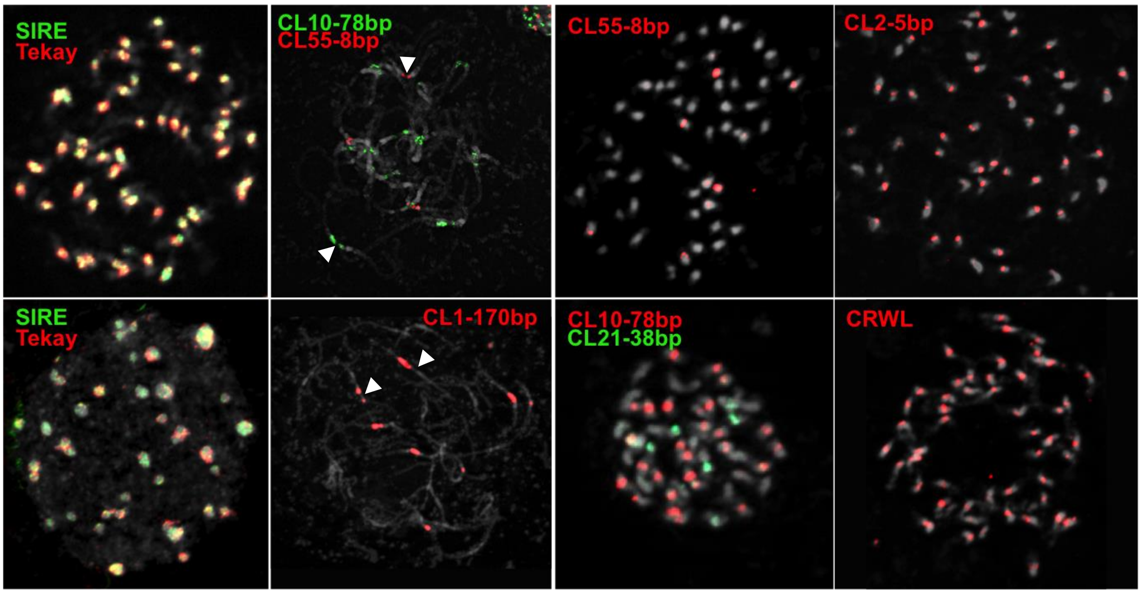

Supplementary Figure 2. FISH with main repeats of WL genome showing the distribution of pericentromeric and centromeric repeats. Arrowheads point to core centromeres. CRWL, CL2-5bp, CL10-78bp, CL21-38bp and CL55-8bp repeats localize specifically to core centromeres, while CL1-170bp repeat localizes aside core centromeres.

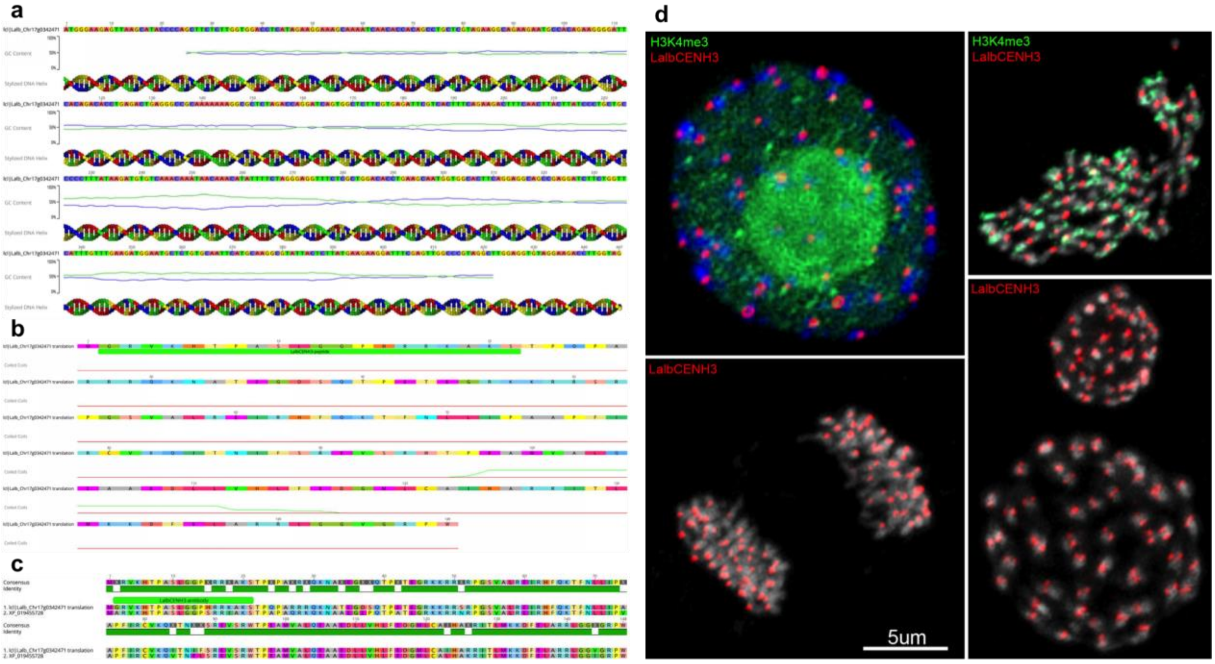

Supplementary Figure 3. Characterization of *Lupinus albus* CENH3. (a) Coding DNA sequence; **(b)** Deduced amino acid sequence; **(c)** Alignment of LalbCENH3 and target peptide site for specific antibody generation. XP_019455728 refers to the Genbank accession number of the *Lupinus angustifolius* CENH3 sequence. **(d)** Immunostaining of *L. albus* chromosomes with LaCENH3 (red) and H3K4me3 (green).

a

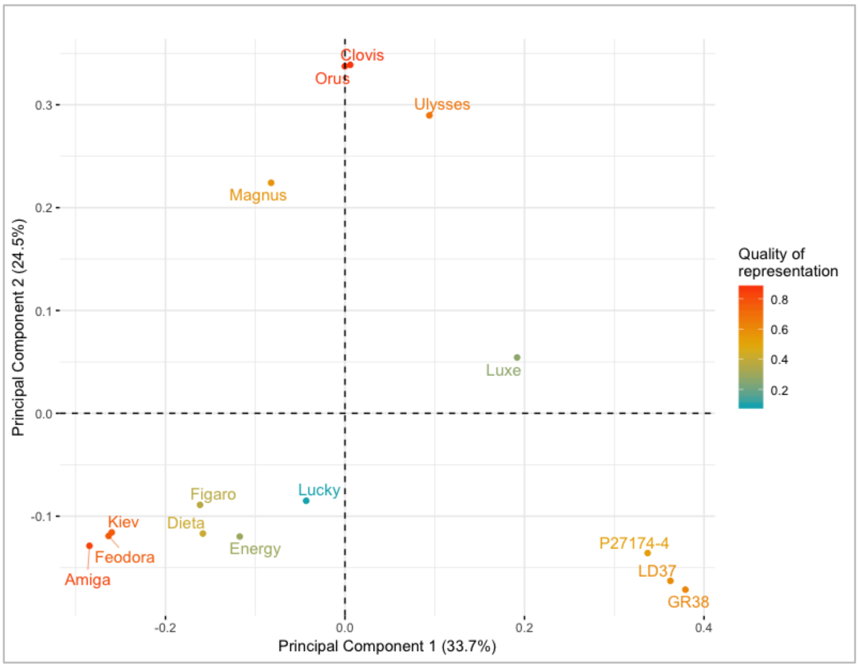

b

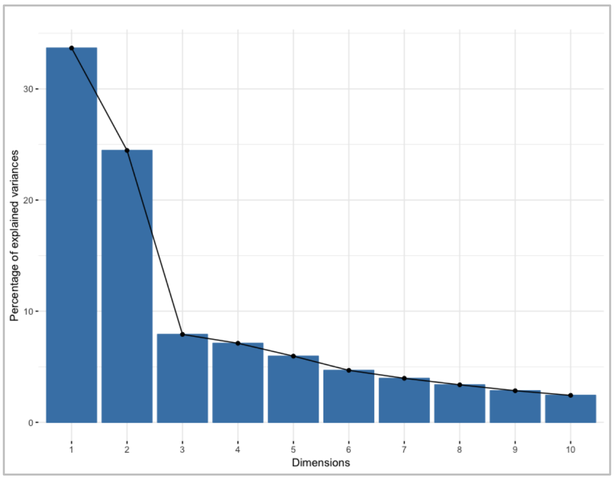

Supplementary Figure 4. Principal component analysis of 15 white lupin accessions. (a) Screen plot showing the percentage of variances explained by the 10 first principal components. **(b)** Scatter plot of the distribution of the 15 accessions in the two first principal components, explaining 58.2% of the total variance. The distribution of the accessions clearly reflects the pattern observed in the phylogenetic tree.

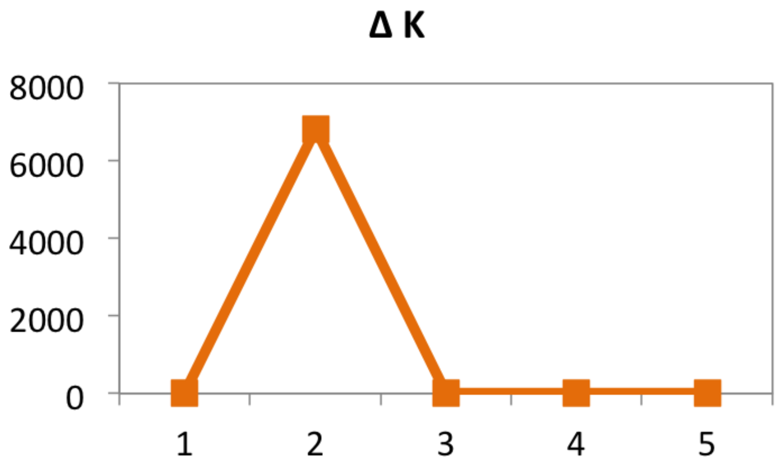

Supplementary Figure 5. Admixture analysis of 15 *L. albus* accessions – Evanno's test. Delta K values calculated by Evanno method using Structure Harvester v0.6.94. ΔK based on the rate of change of LnP(D) between successive K. The value of ΔK reached the highest (68117) when K = 2, indicating that 15 white lupin accessions might be divided into two subpopulations.

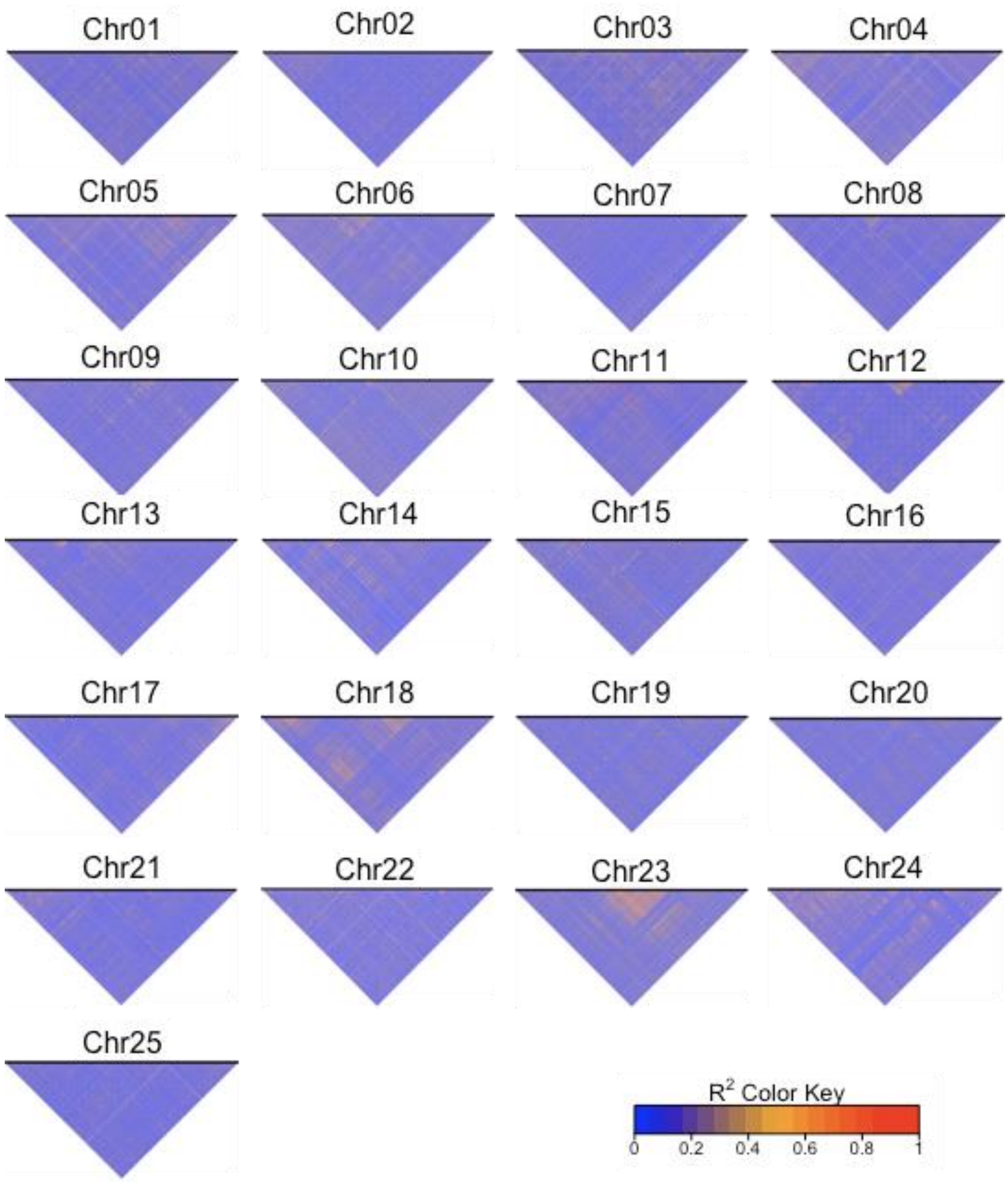

Supplementary Figure 6. Linkage disequilibrium analysis for the 25 white lupin chromosomes. Genotypic linkage disequilibrium (LD, r^2) represented by heatmap for all white lupin chromosomes. The black bar in the top of the figure is a graphical representation of physical location of the SNPs on the chromosomes. The color coding represents high LD (red) and low LD (blue).

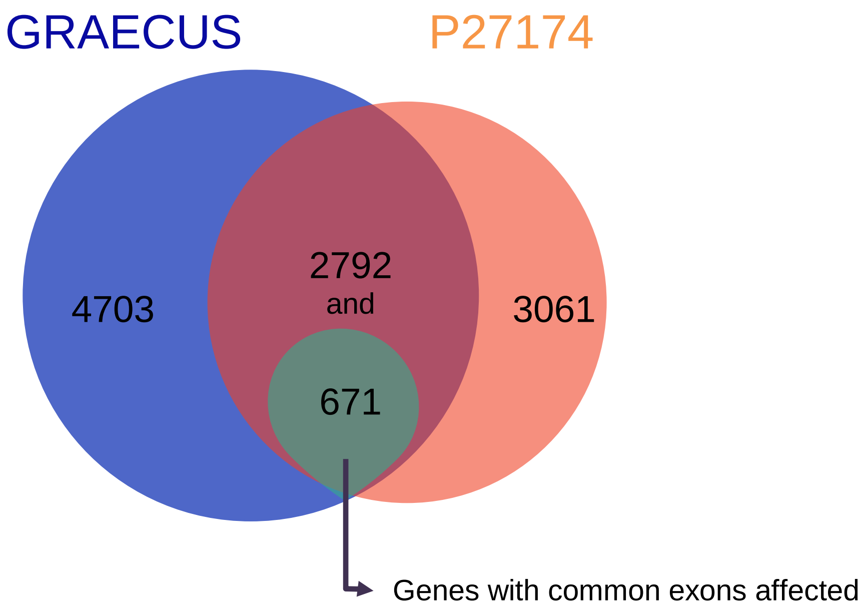

Supplementary Figure 7. Genes affected by structural variations in GRAECUS and P27174 when compared with AMIGA reference genome. The number of genes affected by structural variations in the two accessions are represented in the diagram. The overlapped intersection shows the genes that are common altered by the variations in the two genotypes, highlighting the number of genes with common exons affected.

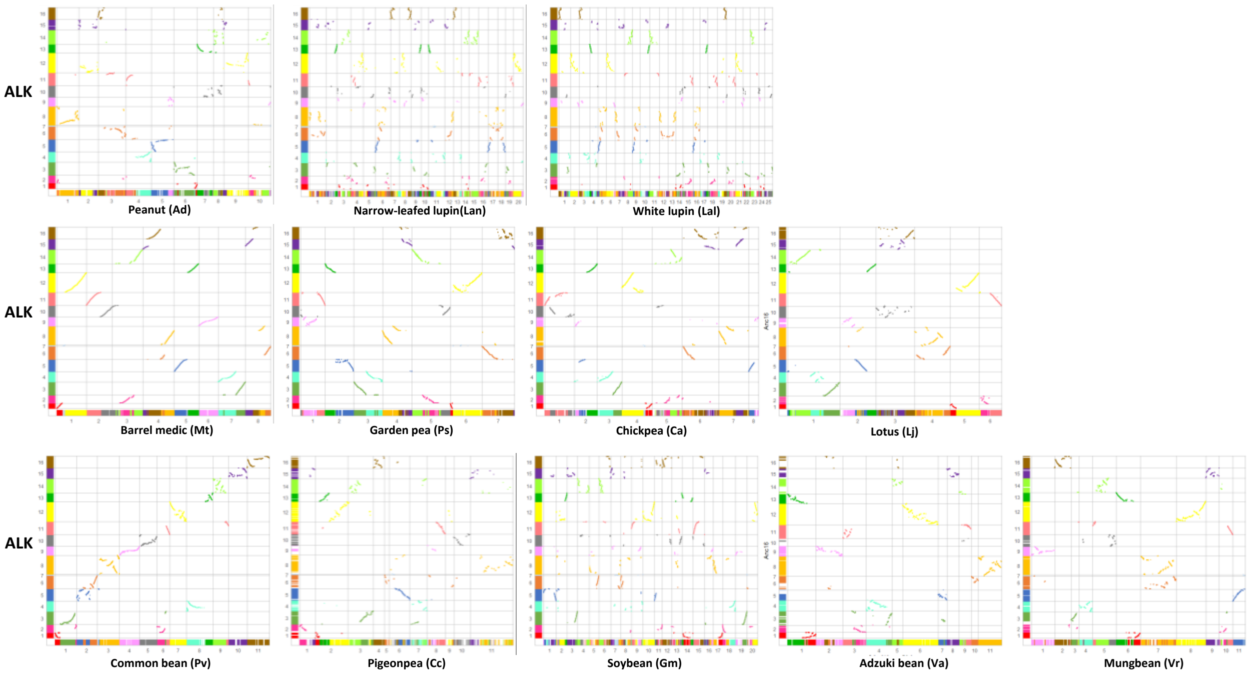

Supplementary Figure 8. Legume genome synteny. Dot plot-based deconvolution of the synteny relationships between ALK (y-axis) and the 12 legume genomes (x-axis). The chromosomes are depicted as a mosaic of a 16 color-code reflecting the 16 inferred CARs in ALK. The synteny relationships identified between the ancestral genome and the modern species are illustrated with colored diagonals in dot plots.

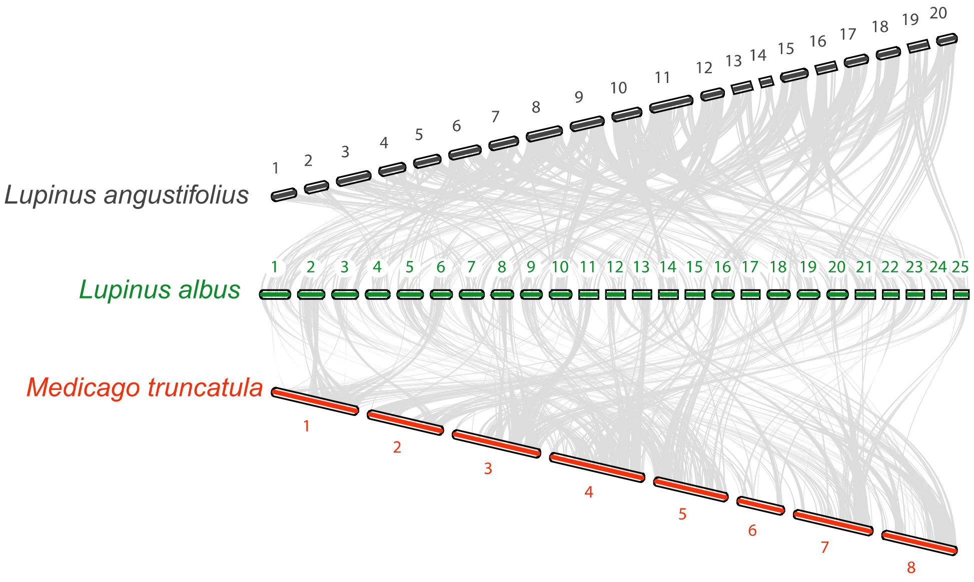

Supplementary Figure 9. Synteny blocks shared between white lupin, its close relative *L. angustifolius* and the legume model *Medicago truncatula.* We identified *ca*. 420 syntenic blocks between white Lupin *M. truncatula* genomes and 250 synteny blocks between the two lupin species genomes.

a

b

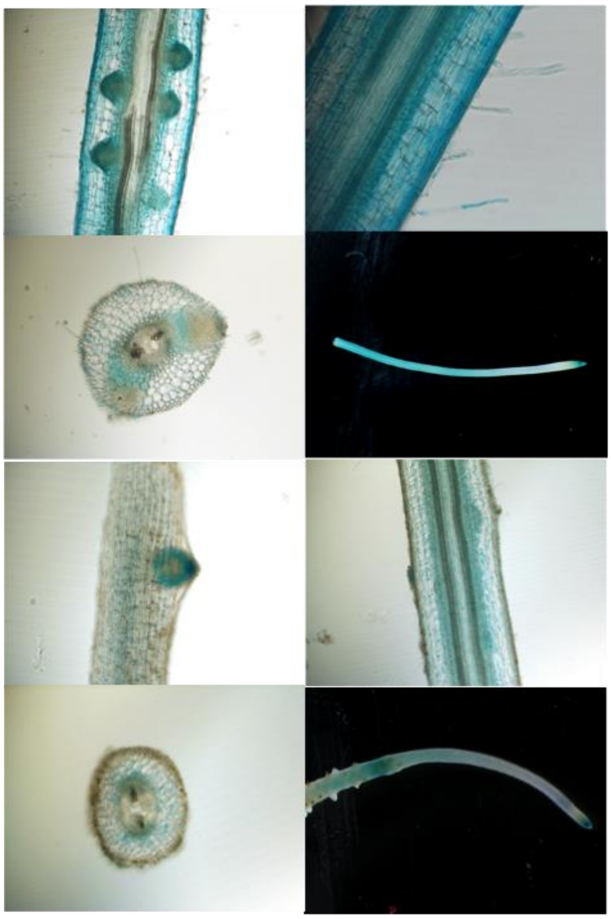

Supplementary Figure 10. Symbiotic genes remain active in white lupin roots. Expression pattern of *pLaSYMRK:GUS* **(a)** and *pLaCCamK:GUS* **(b)** in *hairy roots* of 4-week-old transformed plants grown in low nitrate condition and presence of *Bradyrhizobium lupini LL13.*

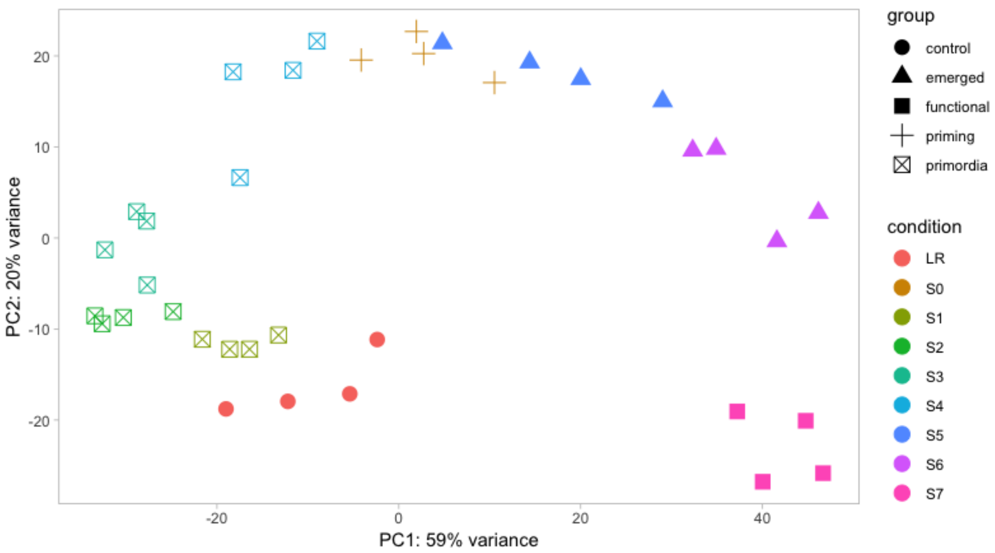

Supplementary Figure 11. Scatterplot of the two first principal components over counts matrices of cluster root detailed development RNAseq. The two axes represent the cumulative variance of principal component 1 (PC1, axis x) and principal component 2 (PC2, axis y). The samples are represented by colour (cluster root type) and shapes (developmental stage of the cluster root). The four biological replicates of each condition are grouped together, displaying the uniformity of the sampling method.

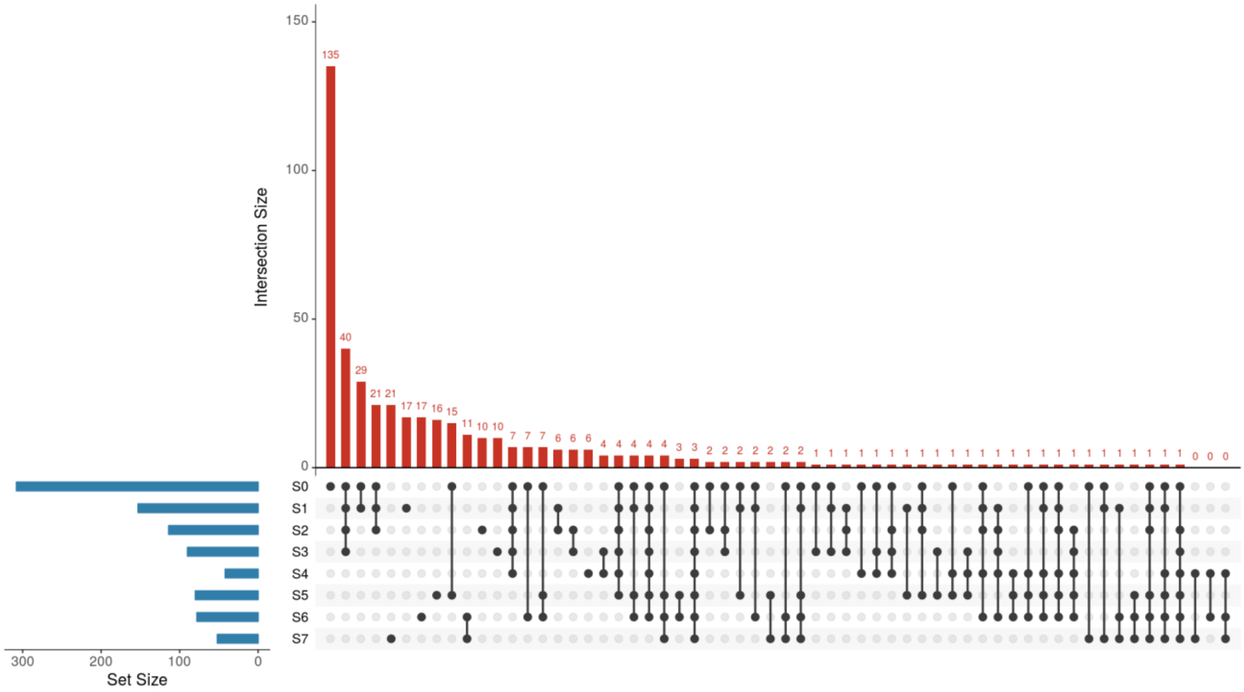

Supplementary Figure 12. Comparative matrix layout of down-regulated genes in the 8 CR segments. Black dots indicate which sample parts (S0 to S7) are grouped and the number of down-regulated genes in the group is indicated on top of each bar. Set size indicates the total number of differentially expressed genes for each sampled fragment.

a

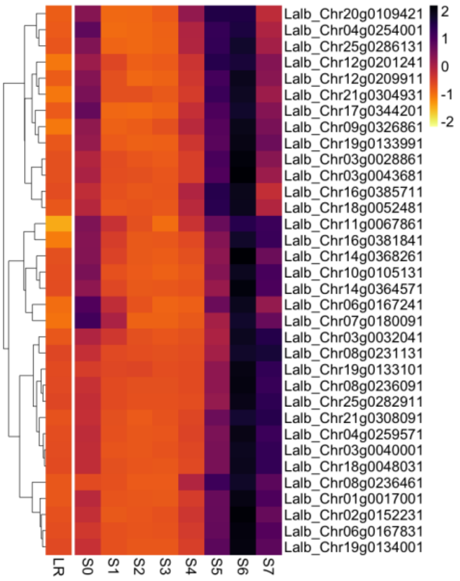

b

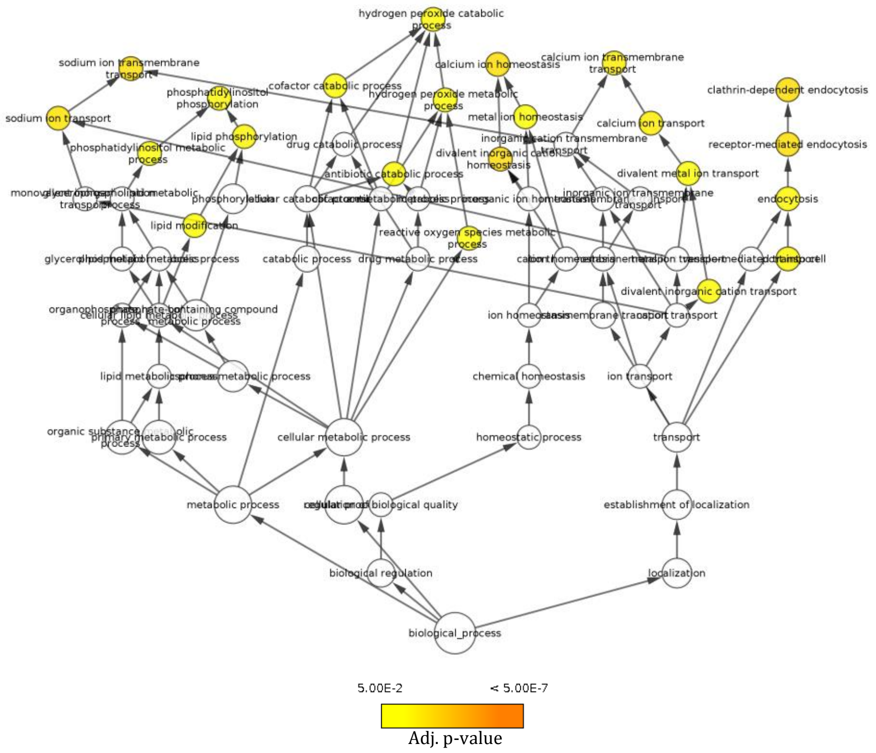

Supplementary Figure 13. GO terms enrichment for the genes up regulated in region S6. **(a)** Heatmap of the 34 genes up-regulated in the zone S6 of the cluster root. **(b)** Network of the GOs of these genes. The colour represents the p-value.

a

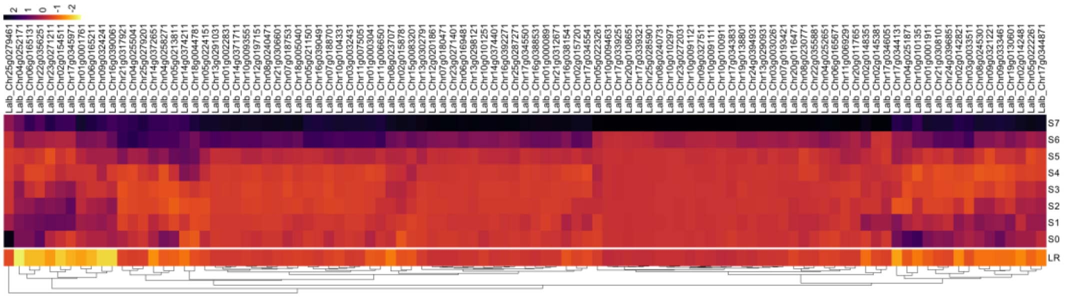

b

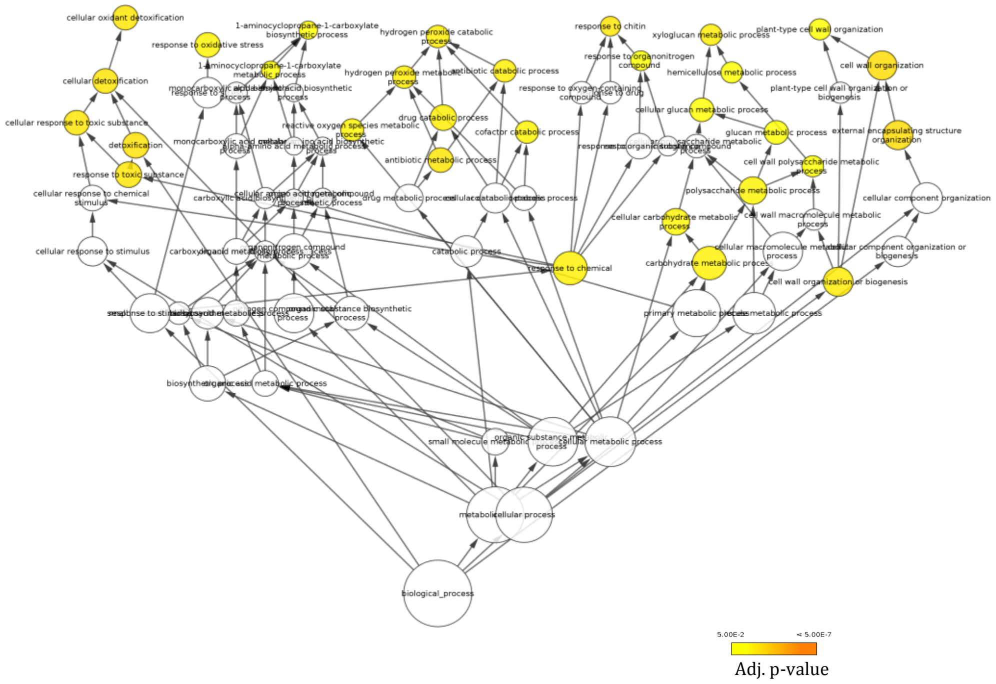

Supplementary Figure 14. GO terms enrichment for the genes up regulated in region S7. (a) Heatmap of the 101 genes up-regulated in the zone S7 of the cluster root. **(b)** Network of the GOs of these genes. The colour represents the p-value.

a

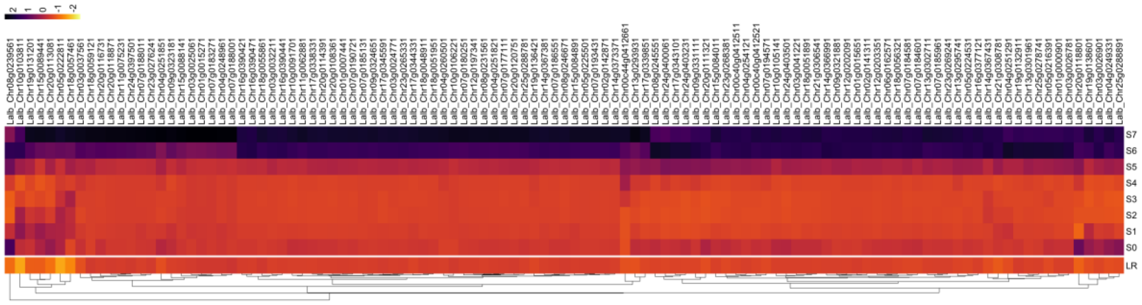

b

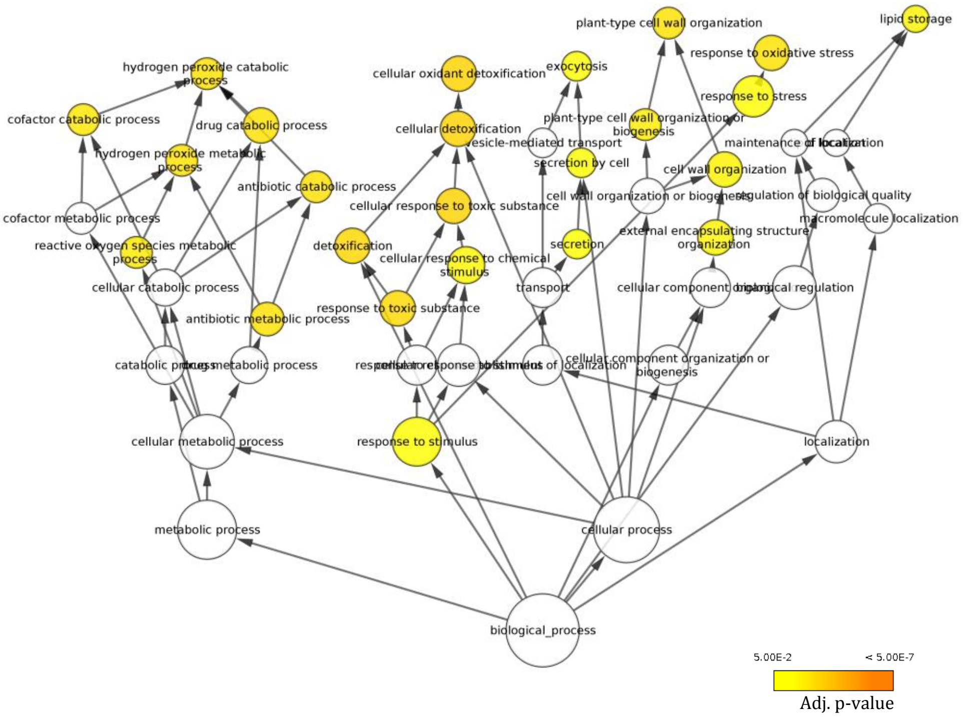

Supplementary Figure 15. GO terms enrichment for the genes up regulated in region S6 and S7. (a) Heatmap of the 111 genes up-regulated in the zones S6 and S7 of the cluster root. **(b)** Network of the GOs of these genes. The colour represents the p-value.

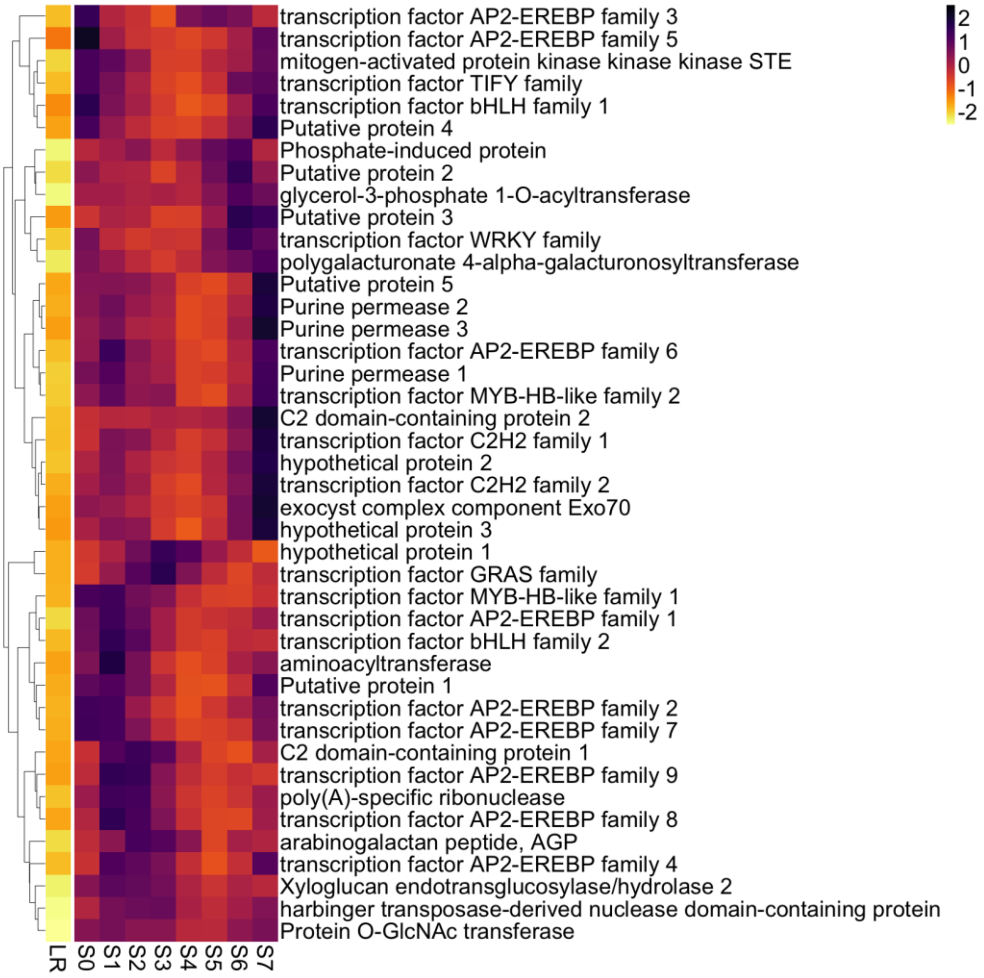

Supplementary Figure 16. Group of 42 genes overexpressed in all cluster root zones. Automatic annotation is shown. There is an enrichment of transcription factors (43%).

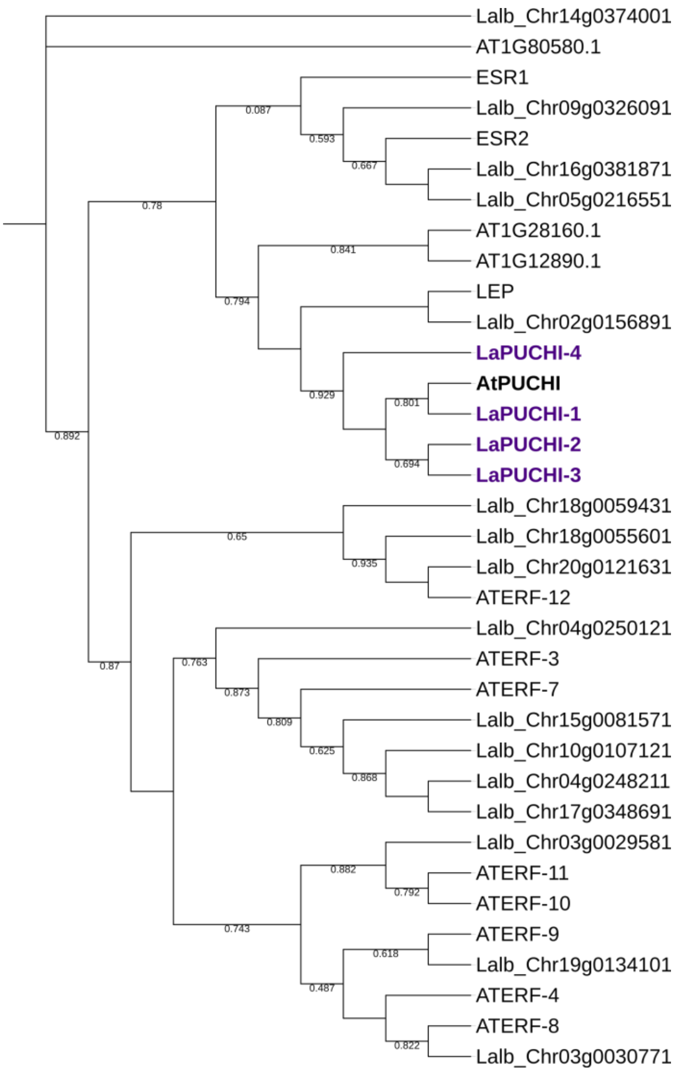

Supplementary Figure 17. Maximum likelihood phylogenetic tree of Arabidopsis ERF (ethylene response factor) subfamily B-1 of ERF/AP2 transcription factor family and white lupin orthologs. These proteins contain an AP2 domain. There are 15 members in this subfamily in Arabidopsis and there are 20 orthologs in white lupin genome. The Arabidopsis thaliana PUCHI (*AtPUCHI*) is highlighted, as well the four white lupin homologs named as *LaPUCHI-1* (Lalb_Chr07g0177601), *LaPUCHI-2* (Lalb_Chr13g0303751), *LaPUCHI-3* (Lalb_Chr187g0059441) and *LaPUCHI-4* (Lalb_Chr15g0086951).

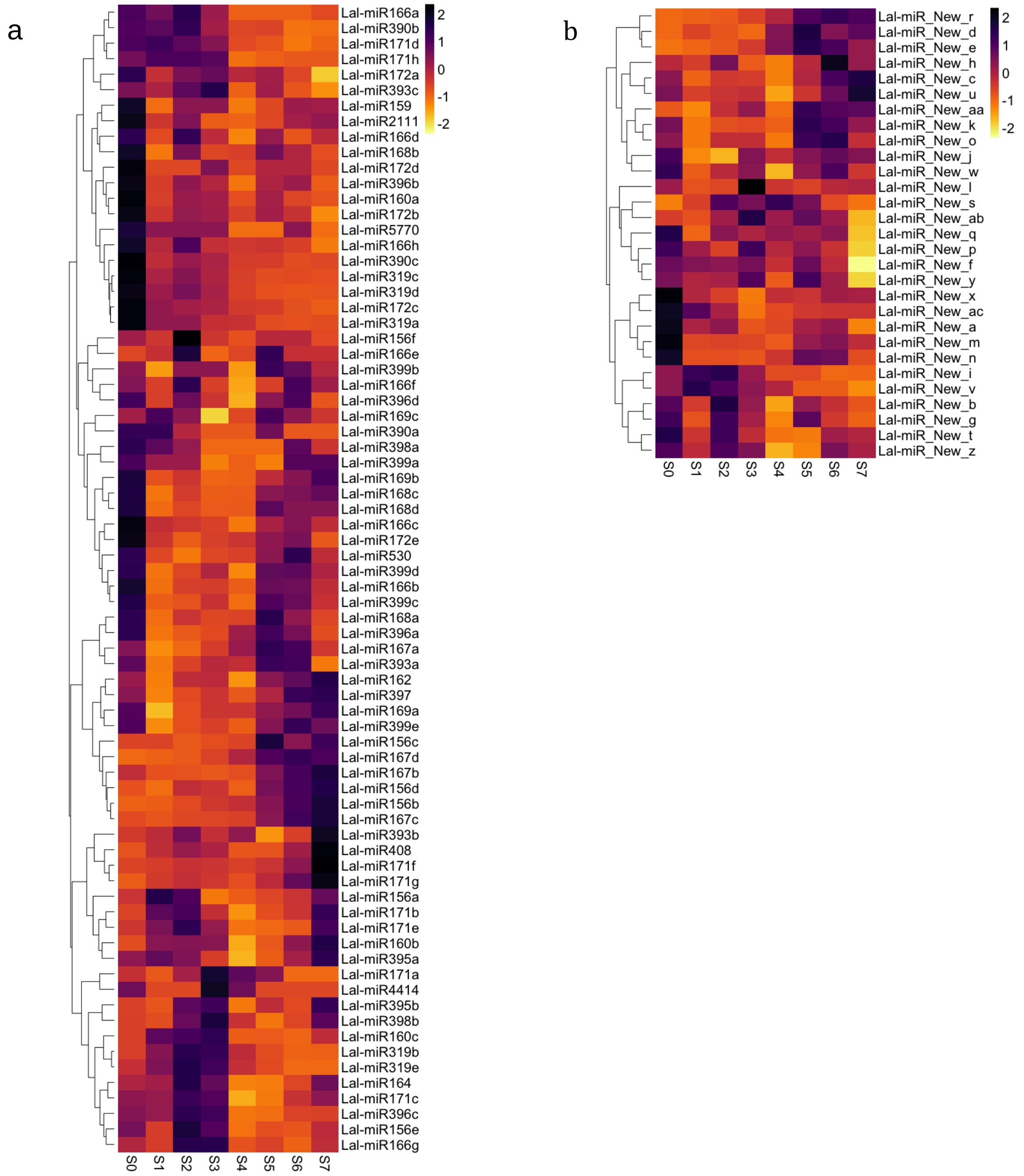

Supplementary Figure 18. Heatmap of expression pattern of known miRNA families in the cluster root zones. 103 miRNA cluster families were identified in the CR, out of which 74 are known **(a)** and 28 are new-described miRNA cluster families **(b)**. More information about the white lupin miRNA cluster families can be found in Supplementary Table 16.

a

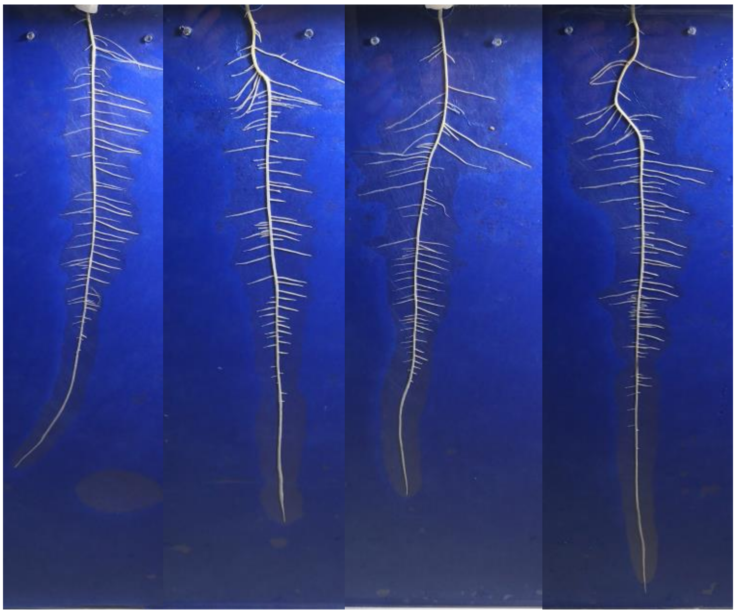

b

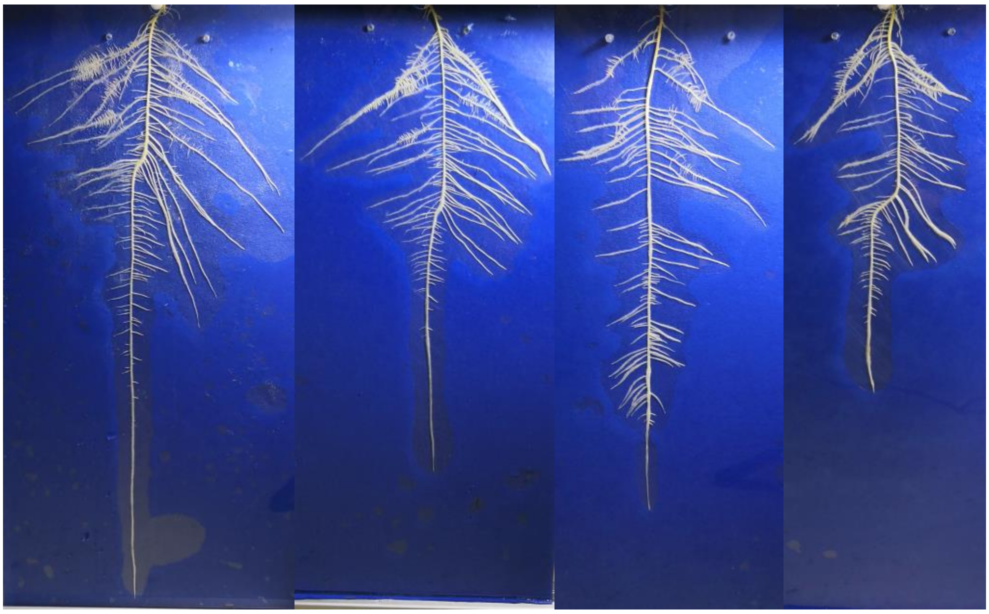

Supplementary Figure 19. Root system architecture of wild accession GRAECUS (a) and *cv*. AMIGA (b). Four representative images of 11-day-old white lupin grown vertically on a blotting paper in low phosphate conditions.

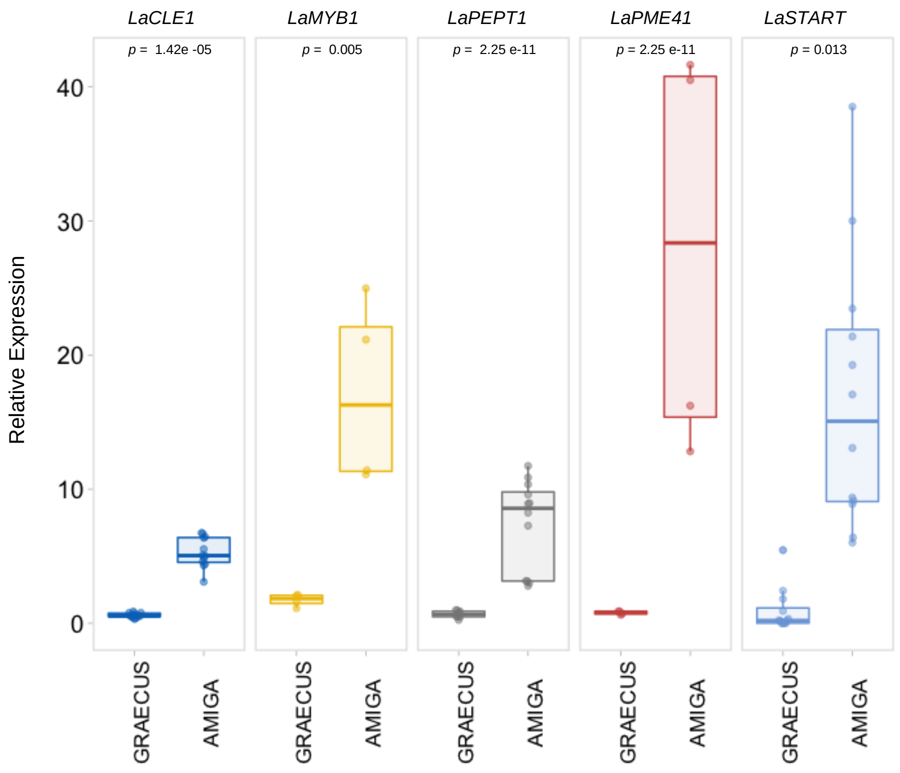

Supplementary Figure 20. Relative expression level in top lateral roots of AMIGA and GRAECUS of 5 genes overexpressed in the cluster roots S1 developmental zone. The *L. albus* genes *LaSTART* (Lalb_Chr20g0121101), *LaCLE-1* (Lalb_Chr19g0133921), *LaPEPT-1* (Lalb_Chr16g0388291), *LaPME41*(Lalb_Chr06g0166531) and *LaMYB1* (Lalb_Chr20g0122341) are overexpressed in the top lateral roots of the cultivated variety AMIGA. This suggests that activation of key regulatory genes can trigger the early establishment of cluster roots. Box edges represent the 0.25 quantile and 0.75 quantile with the median values shown by bold lines. Whiskers extend to data no more than 1.5 times the interquartile range, and remaining data are indicated by dots. n=6.

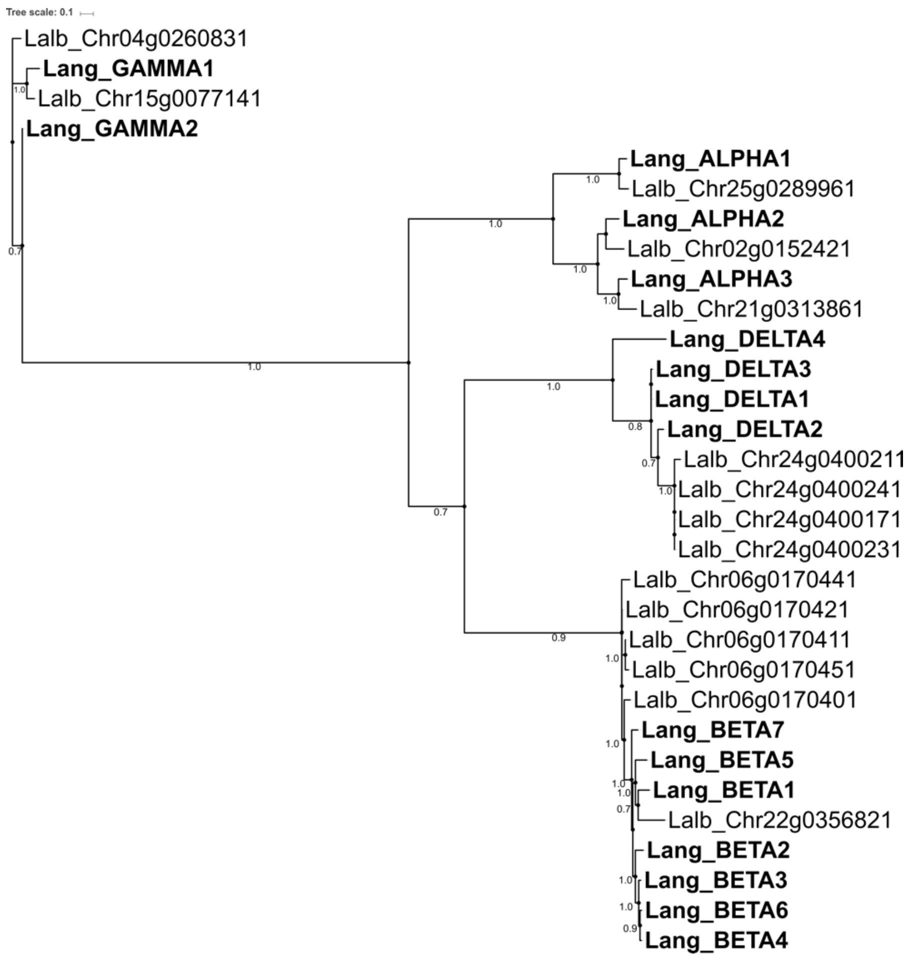

Supplementary Figure 21. White lupin conglutins. Maximum likelihood phylogenetic tree of conglutin orthologs of *L. albus* and *L. angustifolius* (in bold).

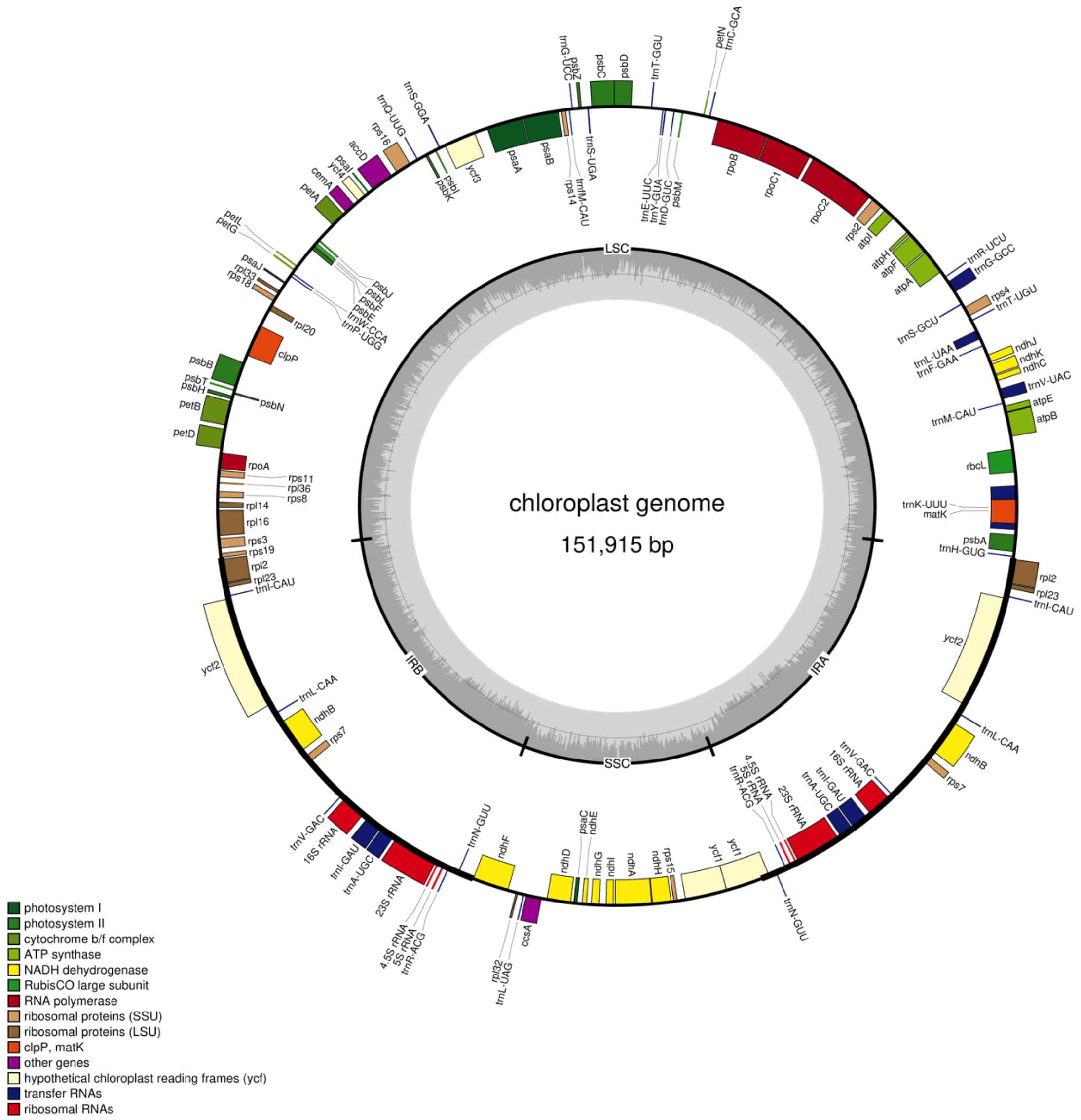

Supplementary Figure 22. A gene map of *Lupinus albus* chloroplast genome. Genomic features on transcriptionally clockwise and counter-clockwise strands are drawn on the inside and outside of the circle, respectively. Genes belonging to different functional groups are color-coded. GC content is represented on the inner circle by a dark gray plot, whereas the light shading represents the AT content.

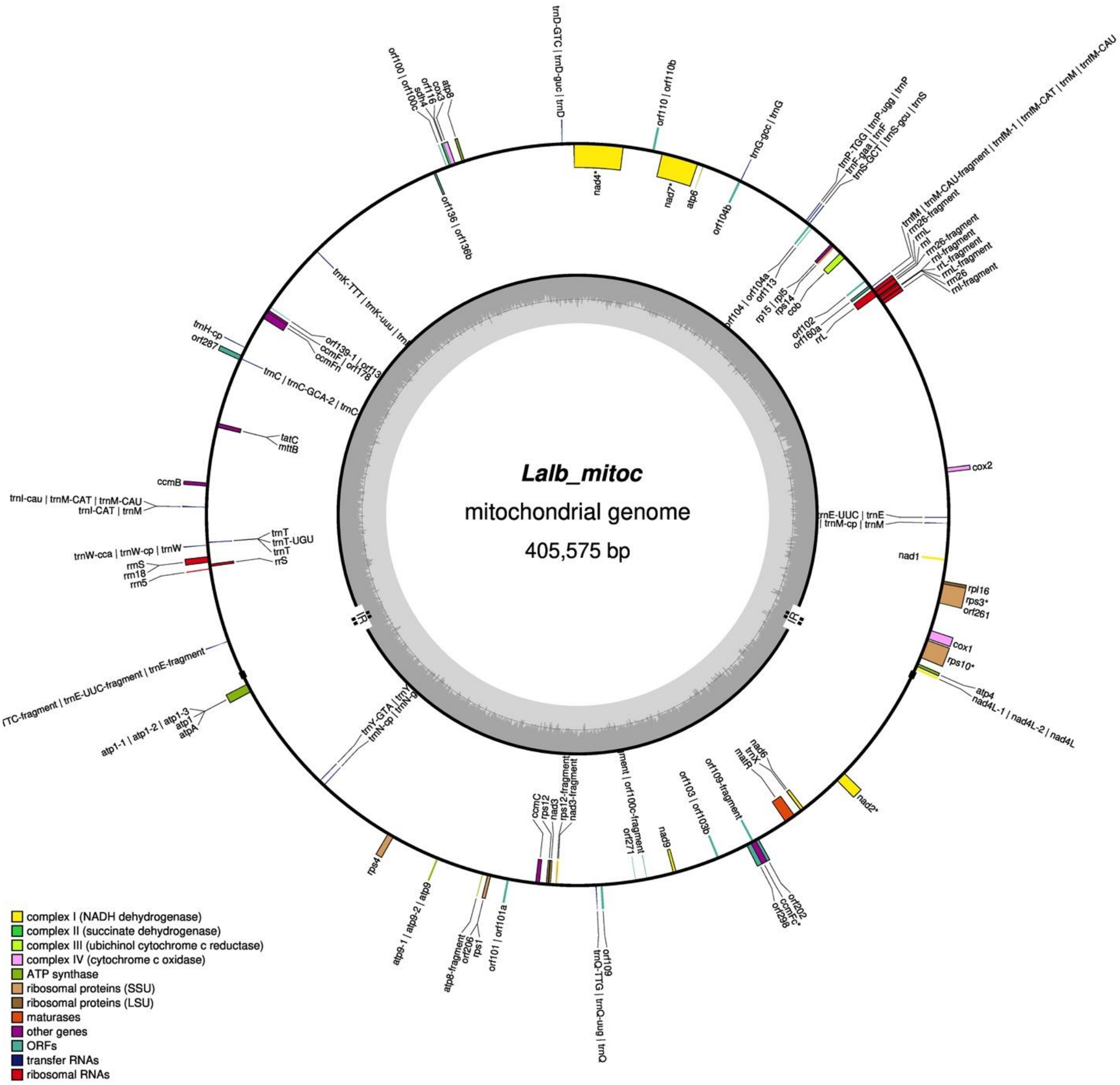

Supplementary Figure 23. A gene map of *Lupinus albus* mitochondrial genome. Genomic features on transcriptionally clockwise and counter-clockwise strands are drawn on the inside and outside of the circle, respectively. Genes belonging to different functional groups are color-coded. GC content is represented on the inner circle by a dark gray plot, whereas the light shading represents the AT content.

Supplementary Table 1. PacBio White lupin genome sequencing data.

	Number of reads	Max read length (bp)	N50 (bp)	Mean read length (bp)	Median read length (bp)	Sequence data per SMRT cell (Gb)
**Max**	733 203	18 3920	17 575	11 825	10 795	6.67
**Mean**	345 841	89 407	15 047	9 394	7 842	3.14

Maximal and mean values (from 30 SMRT cells) relative to sequencing efficiency are indicated. The N50 value indicates that 50% of the sequenced nucleotides are contained in reads equal to or larger than this value.

Supplementary Table 2. Summary of data generated in the RNA sequencing of 10 samples of white lupin tissues.

Sample	# Filtered reads	Sequence data (Gb)
AMIGA Root -P	28 699 273	7.17
AMIGA Root +P	38 270 774	9.57
AMIGA Cluster Root	24 488 390	6.12
AMIGA Pods	22 075 950	5.52
AMIGA Flower	27 047 695	6.76
AMIGA Seeds	24 521 334	6.13
AMIGA Lateral Roots	35 512 338	8.88
AMIGA Primary Root	24 777 784	6.19
AMIGA Nodulated Root	26 467 897	6.62
AMIGA Leaves	25 128 577	6.28
Total	276 990 012	69.25
Mean	27 699 001	6.92

Supplementary Table 3. Summary of final assembly and annotation of white lupin cv. AMIGA.

	**Total assembly**	
**Total number of nucleotides**	**450,969,408**	
Number of nucleotides (without 'N')	448,704,169	
	Per cent GC	33.71
**Total number of genes**	**41,387**	
	Total nucleotides (bp)	132,508,751
	**Protein coding genes**	
**Number of protein coding genes**	**38,258**	
	Mean gene length (bp)	3,411
	Coding nucleotides (bp)	42,471,860
	Per cent genes with introns	78.00
	Per cent genes with 5'-UTR	84.00
	Per cent genes with 3'-UTR	83.00
Exons		
	Mean number per gene	5.13
	Mean length (bp)	309.47
	GC per cent	39.28
Introns		
	Mean number per gene	4.13
	Mean length (bp)	441.78
	GC per cent	32.19
CDS		
	Mean length (bp)	1,110.14
	Min length (bp)	123.00
	Max length (bp)	15,306.00
	GC per cent	41.86
five_prime_UTR		
	Mean length (bp)	229.85
	GC per cent	34.44
three_prime_UTR		
	Mean length (bp)	340.45
	GC per cent	32.50
	**Non protein coding genes**	
**Number of non-protein coding genes**	**3,129**	
	Mean ncRNA gene length (bp)	640.55
	Min length (bp)	23
	Max length (bp)	14,112
	GC percent	41.57
	Percent ncRNA genes with introns	-
	Mean exon number per ncRNA gene	1.00
	**Intergenic (inter protein-coding genes)**	
	Mean length	7,695
	GC per cent	32.68

Supplementary Table 4. **List of primers and direct-labeled oligo-probes for white lupin studied repeats.**

Primer name	Sequence	Annotation.:
CL1-170bp-F1	GTGACATATGCATTTGGGGTAG	
CL1-170bp-R1	ACGTCCAATTCTGATATACGGAA	
CL10-78bp-F1	AAAASTAGCCACAAARAAGT	Centromeric DNA
CL10-78bp-R1	MAGCACAAATAACTCCCAA	Centromeric DNA
INT-Tekay-SC1-F1	CAGAAAGCCAAGATTGAGCATC	
INT-Tekay-SC1-R1	CCCAATATCAAGTTCTCCCCAG	
RT-Tekay-SC1-F1	GTGCGCCTGTTTTGTTGGTT	
RT-Tekay-SC1-R1	TGTTCCTCTCGGCTTTTGGAG	
INT-CRM-SC5-F1	CATGGGCTTTACACGCCCTT	Centromeric DNA
INT-CRM-SC5-R1	GTAAGTCCAGCGGGGTCAAA	Centromeric DNA
RT-CRM-SC5-F1	CAGAGCATGAGCCCTTGTG	Centromeric DNA
RT-CRM-SC5-R1	TGATCCACGCAAAAGGAACA	Centromeric DNA
RT-CRM-SC5-F2	CAACAGAGCATGAGCCCTTG	Centromeric DNA
RT-CRM-SC5-R2	TGACATGATCCACGCAAAAGG	Centromeric DNA
RT-SIRE-SC3-F1	ATTGAGGATGTGCACTGGGG	
RT-SIRE-SC3-R1	CCAAGCCTCTTTCGAACCCT	
INT-SIRE-SC3-F1	CGCGTTGCACACATCCATAT	
INT-SIRE-SC3-R1	TTTGTTGTGGAGTGCGAGGA	
**Oligo-probe name**	**Sequence and fluorescent dye**	
CL1-sat170bp	[Cy3]GTATATCMGAATTGGACGTRTGACATAT GM	
CL10-sat78bp	[Cy3]MAAATWACTCCCAAACTTSTTTGTGGC	Centromeric DNA
CL21-sat38bp	[FAM]GAAAAGTAGCCAAACAAACAAAAG	Centromeric DNA
CL2-microsat5bp	[Cy3]GGATAGGATAGGATAGGATAGGATA	Centromeric DNA
CL55-sat8bp	[Cy3]CTAAACTCCTAAACTCCTAAACTCCT	Centromeric DNA

Supplementary Table 5. **Detailed repetitive DNA composition of the *Lupinus albus* genome**

<table>
<tr><th colspan="3">Class</th><th>Genomic abundance [%]</th></tr>
<tr><td colspan="2" rowspan="6">LTR retroelements Ty1/copia</td><td>Ale</td><td>0.18</td></tr>
<tr><td>Ikeros</td><td>1.21</td></tr>
<tr><td>Ivana</td><td>0.20</td></tr>
<tr><td>SIRE</td><td>6.26</td></tr>
<tr><td>TAR</td><td>0.43</td></tr>
<tr><td>Tork</td><td>0.96</td></tr>
<tr><td rowspan="4">LTR retroelements Ty3/gypsy</td><td rowspan="2">non-chromovirus</td><td>Athila</td><td>1.24</td></tr>
<tr><td>Ogre</td><td>0.27</td></tr>
<tr><td rowspan="2">Chromovirus</td><td>CRM</td><td>3.40</td></tr>
<tr><td>Tekay</td><td>16.68</td></tr>
<tr><td colspan="2" rowspan="4">DNA transposons</td><td>EnSpm CACTA</td><td>0.34</td></tr>
<tr><td>hAT</td><td>0.04</td></tr>
<tr><td>MuDR Mutator</td><td>0.27</td></tr>
<tr><td>Helitron</td><td>0.11</td></tr>
<tr><td colspan="3">Other TEs</td><td>11.64</td></tr>
<tr><td colspan="2" rowspan="2">rDNA</td><td>35S rDNA</td><td>2.43</td></tr>
<tr><td>5S rDNA</td><td>0.15</td></tr>
<tr><td colspan="3">Tandem repeats</td><td>14.71</td></tr>
<tr><td colspan="3">Total</td><td>60.52</td></tr>
</table>

Supplementary Table 6. Satellite DNA genomic abundance, monomer length and features.

satDNA clusters	Genomic abundance [%]	Monomer length (bp)	Annotation.:
CL1, CL52, CL127	6,129	170	Most abundant satDNA; No enrichment with CENH3-immunoprecipitated DNA
CL2	5,473	5	Relative enrichment with CENH3-immunoprecipitated DNA
CL10	1,290	78	Enrichment with CENH3-immunoprecipitated DNA
CL21	0,766	38	Enrichment with CENH3-immunoprecipitated DNA
CL53	0,303	24	No enrichment with CENH3-immunoprecipitated DNA
CL55	0,285	8	High enrichment with CENH3-immunoprecipitated DNA
CL68	0,190	7	Telomere motif
CL77	0,117	36	No enrichment with CENH3-immunoprecipitated DNA
CL85	0,085	76	No enrichment with CENH3-immunoprecipitated DNA
CL114	0,027	247	No enrichment with CENH3-immunoprecipitated DNA
CL118	0,024	182	Similarity to CL10 and CL21; No enrichment with CENH3-immunoprecipitated DNA
CL121	0,023	918	No enrichment with CENH3-immunoprecipitated DNA
Total	14,713		

Supplementary Table 7. White lupin accessions used in this study.

Genotype	Accession type	Sowing time	Seed color	Country of origin
Amiga	Cultivated	Spring	White	Chile
Clovis	Cultivated	Winter	White	France
Dieta	Cultivated	Spring	White	Ukraine
Energy	Cultivated	Spring	White	France
Feodora	Cultivated	Spring	White	Germany
Figaro	Cultivated	Spring	White	France
GR38 (Megalopolis)	Non-domesticated	Winter	Marble	Greece
GRAECUS	Non-domesticated	Winter	Dark Marble	Greece
Kiev	Cultivated	Spring	White	Ukraine
Lucky	Cultivated	Spring	White	France
Luxe	Cultivated	Winter	White	France
Magnus	Cultivated	Winter	White	France
Orus	Cultivated	Winter	White	France
P27174	Landrace	Winter	White	Ethiopia
Ulysse	Cultivated	Winter	Pink	France

Supplementary Table 8. Summary of re-sequencing of white lupin accession using short reads.

Acession	Number of reads	Sequencing depth (x)
Clovis	85072762	28.31
Dieta	88382474	29.41
Energy	120147660	39.98
Feodora	111688474	37.17
Figaro	88274518	29.38
GR38	123360898	41.05
GRAECUS	148837522	49.53
Kiev	247673360	82.42
Lucky	107121722	35.65
Luxe	89069200	29.64
Magnus	216658716	72.10
Orus	93626420	31.16
P27174	256870228	85.48
Ulysse	102510626	34.11

Supplementary Table 9. Summary of SNP distribution along the 25 white lupin chromosomes.

Chromosome	Chr. size (Mb)	SNPs numbers	SNP density (per Kb)
Lalb_Chr01	23.521	251648	10.70
Lalb_Chr02	17.964	189793	10.57
Lalb_Chr03	21.293	209454	9.84
Lalb_Chr04	16.824	178403	10.60
Lalb_Chr05	17.322	166236	9.60
Lalb_Chr06	17.860	180397	10.10
Lalb_Chr07	17.716	173366	9.79
Lalb_Chr08	16.982	195090	11.49
Lalb_Chr09	15.103	178221	11.80
Lalb_Chr10	19.347	202619	10.47
Lalb_Chr11	20.058	178485	8.90
Lalb_Chr12	17.349	206723	11.92
Lalb_Chr13	16.178	185099	11.44
Lalb_Chr14	14.546	168640	11.59
Lalb_Chr15	20.045	236105	11.78
Lalb_Chr16	14.402	152366	10.58
Lalb_Chr17	15.015	160319	10.68
Lalb_Chr18	20.222	168727	8.34
Lalb_Chr19	18.232	189237	10.38
Lalb_Chr20	18.249	217106	11.90
Lalb_Chr21	15.132	149269	9.86
Lalb_Chr22	15.014	182937	12.18
Lalb_Chr23	16.633	205286	12.34
Lalb_Chr24	12.487	121927	9.76
Lalb_Chr25	16.406	195986	11.95

Supplementary Table 10. Statistics of GRAECUS and P27174 *de novo* genome assembly.

**Data acquisition**			
	Total number of Reads	Total sequencing length (Gb)	N50 reads length (Kb)
GRAECUS	1 280 690	12.45	13.6
P27174	1 739 129	14.59	11.8

**Assembly features**			
	Total number of Contigs	Total size assembled (Mb)	N50 (Kb)
GRAECUS	5385	428.06	251.63
P27174	5869	458.23	207.64

Supplementary Table 11. Genes and primer pairs used to study relative expression of cluster root initiation genes in AMIGA and GRAECUS

Gene	Id	Primer forward	Primer reverse
LaPUCHI-1	Lalb_Chr07g0177601	gagggagaagaaagcaagca	gcagcatatctaccccaagg
LaSTART	Lalb_Chr20g0121101	caaccaaaagaaggggttga	tgaggcttcttccctatcagc
LaCLE-1	Lalb_Chr19g0133921	caacaataggcaaaagggttagt	cctagcattgacctctttgaga
LaPEPT-1	Lalb_Chr16g0388291	atgcattcagacccacatca	tttcttctgcatatttgtgacca
LaPME41	Lalb_Chr06g0166531	ggacgggttaccatgtgatt	ggtgtaagtaactcccgtctgtg
LaMYB1	Lalb_Chr20g0122341	aaagagaggccggtttacct	gccaagaatgctatgtagctga
LaHelicase	Lalb_Chr13g0304501	caattctgaaggctatactatgtgc	tcaccagctgctacattaggg

Supplementary References

1. Koren, S. *et al.* Canu: scalable and accurate long-read assembly via adaptive k-mer weighting and repeat separation. *Genome Res.* **27**, 722–736 (2017).
2. Marçais, G. & Kingsford, C. A fast, lock-free approach for efficient parallel counting of occurrences of k-mers. *Bioinformatics* **27**, 764–770 (2011).
3. Liu, B. *et al.* Estimation of genomic characteristics by analyzing k-mer frequency in *de novo* genome projects. *arXiv Prepr. arXiv1308.2012* (2013).
4. Naganowska, B., Wolko, B., Śliwińska, E. & Kaczmarek, Z. Nuclear DNA content variation and species relationships in the genus *Lupinus* (Fabaceae). *Ann. Bot.* **92**, 349–355 (2003).
5. Bennett, M. D., Smith, J. B. & Riley, R. Nuclear DNA amounts in angiosperms. *Philos. Trans. R. Soc. London. B, Biol. Sci.* **274**, 227–274 (1976).
6. Raymond, O. *et al.* The *Rosa* genome provides new insights into the domestication of modern roses. *Nat. Genet.* **50**, 772–777 (2018).
7. Chin, C. S. *et al.* Phased diploid genome assembly with single-molecule real-time sequencing. *Nat. Methods* **13**, 1050–1054 (2016).
8. Walker, B. J., Abeel, T. , Shea, T., Priest, M. & Abouelliel, A. Pilon: an integrated tool for comprehensive microbial variant detection and genome assembly improvement. *PLoS ONE* **9**, 112963 (2014).
9. Tang, H. *et al.* ALLMAPS: robust scaffold ordering based on multiple maps. *Genome Biol.* **16**, 1–15 (2015).
10. Książkiewicz, M. *et al.* A high-density consensus linkage map of white lupin highlights synteny with narrow-leafed lupin and provides markers tagging key agronomic traits. *Sci. Rep.* **7**, 15335 (2017).
11. Abdolzadeh, A., Wang, X., Veneklaas, E. J. & Lambers, H. Effects of phosphorus supply on growth, phosphate concentration and cluster-root formation in three *Lupinus* species. *Ann. Bot.* **105**, 365–374 (2010).
12. Sallet, E., Gouzy, J. & Schiex, T. EuGene: an automated integrative gene finder for eukaryotes and prokaryotes. In *Gene Prediction* (ed. Kollmar, M.) 97–120 (Springer New York, 2019). doi:10.1007/978-1-4939-9173-0_6
13. Pecrix, Y. *et al.* Whole-genome landscape of *Medicago truncatula* symbiotic genes. *Nat. Plants* **4**, 1017–1025 (2018).
14. Bao, W., Kojima, K. K. & Kohany, O. Repbase Update, a database of repetitive elements in eukaryotic genomes. *Mob. DNA* **6**, 11 (2015).

15. Zerbino, D. R. Using the velvet *de novo* assembler for short-read sequencing technologies. *Curr. Protoc. Bioinforma.* **31**, 11.5.1-11.5.12 (2010).

16. Simão, F. A., Waterhouse, R. M., Ioannidis, P., Kriventseva, E. V. & Zdobnov, E. M. BUSCO: assessing genome assembly and annotation completeness with single-copy orthologs. *Bioinformatics* **31**, 3210–3212 (2015).

17. Schläpfer, P. *et al.* Genome-wide prediction of metabolic enzymes, pathways, and gene clusters in plants. *Plant Physiol.* **173**, 2041–2059 (2017).

18. Zheng, Y. *et al.* iTAK: a program for genome-wide prediction and classification of plant transcription factors, transcriptional regulators, and protein kinases. *Mol. Plant* **9**, 1667–1670 (2016).

19. Dai, X., Sinharoy, S., Udvardi, M. & Zhao, P. X. PlantTFcat: an online plant transcription factor and transcriptional regulator categorization and analysis tool. *BMC Bioinformatics* **14**, 321 (2013).

20. Finn, R. D. *et al.* InterPro in 2017- beyond protein family and domain annotations. *Nucleic Acids Res.* **45**, D190–D199 (2016).

21. Bairoch, A. The ENZYME database in 2000. *Nucleic Acids Res.* **28**, 304–305 (2000).

22. Martin, M. Cutadapt removes adapter sequences from high-throughput sequencing reads. *EMBnet.journal* **17**, 10 (2011).

23. Li, H. & Durbin, R. Fast and accurate long-read alignment with Burrows-Wheeler transform. *Bioinformatics* **26**, 589–595 (2010).

24. McKenna, A. *et al.* The genome analysis toolkit: a map reduce framework for analyzing next-generation DNA sequencing data. *Genome Res.* **20**, 1297–1303 (2010).

25. Marques, A. *et al.* Holocentromeres in *Rhynchospora* are associated with genome-wide centromere-specific repeat arrays interspersed among euchromatin. *Proc. Natl. Acad. Sci.* **112**, 13633–13638 (2015).

26. Novak, P., Neumann, P., Pech, J., Steinhaisl, J. & Macas, J. RepeatExplorer: a Galaxy-based web server for genome-wide characterization of eukaryotic repetitive elements from next-generation sequence reads. *Bioinformatics* **29**, 792–793 (2013).

27. Neumann, P., Novák, P., Hoštáková, N. & Macas, J. Systematic survey of plant LTR-retrotransposons elucidates phylogenetic relationships of their polyprotein domains and provides a reference for element classification. *Mob. DNA* **10**, 1 (2019).

28. Kato, A. *et al.* Sensitive fluorescence *in situ* hybridization signal detection in maize using directly labeled probes produced by high concentration DNA polymerase nick translation. *Biotech. Histochem.* **81**, 71–78 (2006).

29. Marques, A., Schubert, V., Houben, A. & Pedrosa-Harand, A. Restructuring of holocentric centromeres during meiosis in the plant *Rhynchospora pubera*. *Genetics* **204**, 555–568 (2016).

30. Weisshart, K., Fuchs, J. & Schubert, V. Structured Illumination Microscopy (SIM) and Photoactivated Localization Microscopy (PALM) to analyze the abundance and distribution of RNA polymerase II molecules on flow-sorted *Arabidopsis* Nuclei. *Bio-protocol* **6**, e1725 (2016).

31. Treangen, T. J. & Salzberg, S. L. Repetitive DNA and next-generation sequencing: computational challenges and solutions. *Nat. Rev. Genet.* **13**, 36–46 (2012).

32. Bradbury, P. J. *et al.* TASSEL: software for association mapping of complex traits in diverse samples. *Bioinformatics* **23**, 2633–5 (2007).

33. Letunic, I. & Bork, P. Interactive tree of life (iTOL) v3: an online tool for the display and annotation of phylogenetic and other trees. *Nucleic Acids Res.* **44**, W242–W245 (2016).

34. R Core Team. R: a language and environment for statistical computing. (2018). http://www.R-project.org/

35. Pritchard, J. K., Stephens, M. & Donnelly, P. Inference of population structure using multilocus genotype data. *Genetics* **155**, 945–959 (2000).

36. Hubisz, M. J., Falush, D., Stephens, M. & Pritchard, J. K. Inferring weak population structure with the assistance of sample group information. *Mol. Ecol. Resour.* **9**, 1322–1332 (2009).

37. Earl, D. A. & vonHoldt, B. M. STRUCTURE HARVESTER: a website and program for visualizing STRUCTURE output and implementing the Evanno method. *Conserv. Genet. Resour.* (2012). doi:10.1007/s12686-011-9548-7

38. Evanno, G., Regnaut, S. & Goudet, J. Detecting the number of clusters of individuals using the software STRUCTURE: a simulation study. *Mol. Ecol.* **14**, 2611–20 (2005).

39. Clayton, D. snpStats: SnpMatrix and XSnpMatrix classes and methods. R package version 1.32.0. *R Packag. version 1.32.0* Code Snippet 3 (2018).

40. Shin, J.-H., Blay, S., McNeney, B. & Graham, J. LDheatmap: an R function for graphical display of pairwise linkage disequilibria between single nucleotide polymorphisms. *J Stat Soft* **16**, Code Snippet 3 (2006).

41. Nattestad, M. & Schatz, M. C. Assemblytics: a web analytics tool for the detection of variants from an assembly. *Bioinformatics* **32**, 3021–3023 (2016).

42. Kurtz, S. *et al.* Versatile and open software for comparing large genomes. *Genome Biol.* **5**, R12 (2004).

43. Pont, C. *et al.* Paleogenomics: reconstruction of plant evolutionary trajectories from modern and ancient DNA. *Genome Biol.* **20**, 29 (2019).

44. Hane, J. K. *et al.* A comprehensive draft genome sequence for lupin (*Lupinus angustifolius*), an emerging health food: insights into plant-microbe interactions and legume evolution. *Plant Biotechnol. J.* **15**, 318–330 (2017).

45. Kreplak, J. *et al.* A reference genome for pea provides insight into legume genome evolution. *Nat. Genet.* **51**, 1411–1422 (2019).

46. Bertioli, D. J. *et al.* The genome sequences of *Arachis duranensis* and *Arachis ipaensis*, the diploid ancestors of cultivated peanut. *Nat. Genet.* **48**, 438–446 (2016).

47. Sato, S. *et al.* Genome structure of the legume, *Lotus japonicus*. DNA Res. **15**, 227–239 (2008).

48. Young, N. D. *et al.* The *Medicago* genome provides insight into the evolution of rhizobial symbioses. *Nature* **480**, 520–524 (2011).

49. Varshney, R. K. *et al.* Draft genome sequence of chickpea (*Cicer arietinum*) provides a resource for trait improvement. *Nat. Biotechnol.* **31**, 240–246 (2013).

50. Singh, N. K. *et al.* The first draft of the pigeon pea genome sequence. *J. Plant Biochem. Biotechnol.* **21**, 98–112 (2012)

51. Schmutz, J. *et al.* Genome sequence of the paleopolyploid soybean. *Nature* **463**, 178–183 (2010).

52. Schmutz, J. *et al.* A reference genome for common bean and genome-wide analysis of dual domestications. *Nat. Genet.* **46**, 707–713 (2014).

53. Kang, Y. J. *et al.* Genome sequence of mungbean and insights into evolution within Vigna species. *Nat. Commun.* **5**, 5443 (2014).

54. Kang, Y. J. *et al.* Draft genome sequence of adzuki bean, *Vigna angularis*. *Sci. Rep.* **5**, 8069 (2015).

55. Salse, J. Ancestors of modern plant crops. *Curr. Opin. Plant Biol.* **30**, 134–142 (2016).

56. Tang, H. *et al.* tanghaibao/jcvi: JCVI v0.7.5. (2017). doi:10.5281/ZENODO.846919. https://github.com/tanghaibao/jcvi/tree/v0.7.5

57. Tang, H. *et al.* Synteny and collinearity in plant genomes. *Science* **320**, 486–488 (2008).

58. Wang, Y., Coleman-Derr, D., Chen, G. & Gu, Y. Q. OrthoVenn: a web server for genome wide comparison and annotation of orthologous clusters across multiple species. *Nucleic Acids Res.* **43**, W78–W84 (2015).

59. Camacho, C. *et al.* BLAST+: architecture and applications. *BMC Bioinformatics* **10**, 421 (2009).

60. Katoh, K. & Standley, D. M. MAFFT Multiple sequence alignment software version 7: improvements in performance and usability. *Mol. Biol. Evol.* **30**, 772–780 (2013).

61. Castresana, J. Selection of conserved blocks from multiple alignments for their use in phylogenetic analysis. *Mol. Biol. Evol.* **17**, 540–552 (2000).

62. Gouy, M., Guindon, S. & Gascuel, O. SeaView version 4: a multiplatform graphical user interface for sequence alignment and phylogenetic tree building. *Mol. Biol. Evol.* **27**, 221–224 (2009).

63. Nguyen, L.-T., Schmidt, H. A., von Haeseler, A. & Minh, B. Q. IQ-TREE: a fast and effective stochastic algorithm for estimating maximum-likelihood phylogenies. *Mol. Biol. Evol.* **32**, 268–274 (2015).

64. Kalyaanamoorthy, S., Minh, B. Q., Wong, T. K. F., von Haeseler, A. & Jermiin, L. S. ModelFinder: fast model selection for accurate phylogenetic estimates. *Nat. Methods* **14**, 587 (2017).

65. Hoang, D. T., Chernomor, O., von Haeseler, A., Minh, B. Q. & Vinh, L. S. UFBoot2: Improving the ultrafast bootstrap approximation. *Mol. Biol. Evol.* **35**, 518–522 (2018).

66. Untergasser, A. *et al.* Primer3-new capabilities and interfaces. *Nucleic Acids Res.* **40**, e115–e115 (2012).

67. Karimi, M., Inzé, D. & Depicker, A. GATEWAY™ vectors for *Agrobacterium*-mediated plant transformation. *Trends Plant Sci.* **7**, 193–195 (2002).

68. Gallardo, C. *et al.* Anatomical and hormonal description of rootlet primordium development along white lupin cluster root. *Physiol. Plant.* **165**, 4–16 (2019).

69. Griesmann, M. *et al.* Phylogenomics reveals multiple losses of nitrogen-fixing root nodule symbiosis. *Science.* **1743**, eaat1743 (2018).

70. Bravo, A., York, T., Pumplin, N., Mueller, L. A. & Harrison, M. J. Genes conserved for arbuscular mycorrhizal symbiosis identified through phylogenomics. *Nat. Plants* **2**, 15208 (2016).

71. Kozomara, A., Birgaoanu, M. & Griffiths-Jones, S. miRBase: from microRNA sequences to function. *Nucleic Acids Res.* **47**, D155–D162 (2018).

72. Livak, K. J. & Schmittgen, T. D. Analysis of relative gene expression data using real-time quantitative PCR and the 2−ΔΔCT method. *Methods* **25**, 402–408 (2001).

73. Foley, R. C. *et al.* Identification and characterisation of seed storage protein transcripts from *Lupinus angustifolius*. *BMC Plant Biol.* **11**, 59 (2011).

74. Dereeper, A. *et al.* Phylogeny.fr: robust phylogenetic analysis for the non-specialist. *Nucleic Acids Res.* **36**, W465–W469 (2008).

75. Duranti, M., Restani, P., Poniatowska, M. & Cerletti, P. The seed globulins of *Lupinus albus*. *Phytochemistry* **20**, 2071–2075 (1981).

76. Fontanari, G. G., Batistuti, J. P., Cruz, R. J. Da, Saldiva, P. H. N. & Arêas, J. A. G. Cholesterol-lowering effect of whole lupin (*Lupinus albus*) seed and its protein isolate. *Food Chem.* **132**, 1521–1526 (2012).

77. Boschin, G., Scigliuolo, G. M., Resta, D. & Arnoldi, A. Optimization of the enzymatic hydrolysis of lupin (*Lupinus*) proteins for producing ACE-inhibitory peptides. *J. Agric. Food Chem.* **62**, 1846–1851 (2014).

78. Jimenez-Lopez, J. C. *et al.* Characterization of narrow-leaf lupin (*Lupinus angustifolius* L.) recombinant major allergen IgE-binding proteins and the natural β-conglutin counterparts in sweet lupin seed species. *Food Chem.* **244**, 60–70 (2018).

79. Words, I., Harrison, J. E. M. & Williams, W. Genetical control of alkaloids in *Lupinus albus*. *Euphytica* **31**, 357–364 (1982).

80. Lin, R. *et al.* Development of a sequence-specific PCR marker linked to the gene ‘pauper’ conferring low-alkaloids in white lupin (*Lupinus albus* L.) for marker assisted selection. *Mol. Breed.* **23**, 153–161 (2009).

81. Phan, H. T. T., Ellwood, S. R., Adhikari, K., Nelson, M. N. & Oliver, R. P. The first genetic and comparative map of white lupin (*Lupinus albus* L.): identification of QTLs for anthracnose resistance and flowering time, and a locus for alkaloid content. *DNA Res.* **14**, 59–70 (2007).
