# Supplementary material for: High-quality genome sequence of white lupin provides insight into soil exploration and seed quality
Source: Nat Commun. 2020 Jan 24;11:492. doi: 10.1038/s41467-019-14197-9 (PMC6981116; doi:10.1038/s41467-019-14197-9)
Supplement: Supplementary file 4 — Description of Additional Supplementary Files [file 41467_2019_14197_MOESM4_ESM.doc]

# Description of Additional Supplementary Files

File name: Supplementary Data 1
Description: Summary of assembly metrics.

File name: Supplementary Data 2
Description: Matrix of genetic distance of 15 varieties of white lupin.

File name: Supplementary Data 3
Description: Summary of structural variants (SV) of P27174 and Graecus when compared with the white lupin reference genome (AMIGA).

File name: Supplementary Data 4
Description: Reconstructed legume ancestral karyotypes.

File name: Supplementary Data 5
Description: Intragenomic syntenic blocks of white lupin.

File name: Supplementary Data 6
Description: OrthoMLC clusters unique of *L. albus* proteins, which did not cluster with proteins of *Arabidopsis thaliana, Medicago truncatula* and *Lupinus angustifolius*.

File name: Supplementary Data 7
Description: Plant genomes covering different Angiosperm orders and symbiotic abilities used in this study.

File name: Supplementary Data 8
Description: Ortolog genes known to be specifically required for arbuscular mycorrhizal symbiosis (myc), root nodule symbiosis (nod) or both associations in 8 different species.

File name: Supplementary Data 9
Description: Clustering of up-regulated genes in the 8 cluster root sections (S0-S7) in comparison to lateral roots.

File name: Supplementary Data 10
Description: Clustering of down-regulated genes in the 8 cluster root sections (S0-S7) in comparison to lateral roots.

File name: Supplementary Data 11
Description: White lupin genes overexpressed in the all the CR sections (S0-S7).

File name: Supplementary Data 12
Description: White lupin miRNA family classification, sequence and mean reads count in the cluster root sections (S0-S7).

File name: Supplementary Data 13
Description: *L. albus* (Lalb) and *L. angustifolius* (Lang) aplha, beta and gamma conglutin orthologs.

File name: Supplementary Data 14
Description: Results from protein mass spectomety analysis (MS/MS) with *L. albus* database and and NCBI prot_viridiplantae database.

File name: Supplementary Data 15
Description: Genes and primer pairs used to study relative expression and alkaloid accumulation on white lupin leaves.

File name: Supplementary Data 16
Description: Genes of the *pauper* region in Chr18, which is responsible for a major low-alkaloid QTL.
