# Supplementary material for: High-quality genome sequence of white lupin provides insight into soil exploration and seed quality
Source: Nat Commun. 2020 Jan 24;11:492. doi: 10.1038/s41467-019-14197-9 (PMC6981116; doi:10.1038/s41467-019-14197-9)
Supplement: Supplementary file 2 — Peer Review File [file 41467_2019_14197_MOESM2_ESM.pdf]

Reviewers' comments:

Reviewer #1 (Remarks to the Author):

Hufnagel et al. "Genome sequence of the cluster root forming white lupin" was published in BioRXiv (July 2019) and reports the first version of the white lupin genome. White lupin is a seed protein-rich crop that has developed an original adaptation to low soil phosphorus. Its genome sequence will help deciphering these traits in this species. The manuscript provides a high quality genome sequence for variety AMIGA, as well as two genome sequences of white lupin germplasm accessions. The authors analysed the structure, evolution, diversity and expression of the white lupin genome. However, the extremely condensed and sometimes over simplified form of the manuscript does not do justice to the work that has been done.

In the abstract and in the text, the use of the term domestication is mostly inadequate. Comparing Graecus and Amiga gives information about the diversity between these two lines. To investigate the impact of domestication, the authors should have included much more wild and cultivated genotypes in their analysis. Similarly, the claim that LaPUCHI-1 was identified as a potential regulator of cluster root establishment is a bit excessive. Authors identified a set of genes (including TFs) regulated in the root region developing cluster roots but to qualify one of these genes as regulator would require more in depth analysis and validation.

Overall, it appears that some parts of the text would deserve more emphasis.

In the paragraph about genome assembly, some background should be given on the advantages of the technologies used. During the annotation process, only the M. truncatula proteome was used. Using the proteomes of other related species such as Glycine max and Arachis duranensis would probably have contributed to improve the annotation. The annotation of repetitive DNA identified 60% of repeats but 25% of these TEs were uncharacterised. Can the authors provide some information about these uncharacterised repeats? The paragraph about the location of the different repeats and the FISH data should be better integrated.

The paloegenomic analysis of the evolution leading to the white lupin genome looks powerful. But more background on the different approaches already published, the rationale for the choice of the approach used, and its comparison with previous reports would be useful to the reader.

The de novo assembly of three genomes of white lupin using the latest long-read technologies is a significant output of this study. The white lupin diversity study should better take advantage of these genome sequences that define a white lupin pangenome. What is the impact of structural variation on the genome? what are the regions impacted and their link with TE rich regions? A more in-depth analysis of these data would probably bring interesting results. Extended data Figure 3a should be included in the main text.

In the diversity study, some background on white lupin region of origin (why pick up lines from Greece, Ethiopia, but also France, Germany, Ukraine), domestication, breeding, and area of production would be really useful to discuss more fully these results. Also, the description of accessions should be revised. Do the 14 accessions really cover a wide range of diversity? 8 accessions come from France… In Supplemental Table 7, Amiga and Feodora appear to come from Chile and Germany but at another place in the manuscript (Suppl. 3.2.1) it is indicated that Amiga and Feodora come from a French breeding program. In the same paragraph (suppl 3.2.1), winter and spring accessions' attributes are mixed up. In different places in the text, the term variety is used irrelevantly, because a wild accession is not a variety. It is surprising that the data on seed trait diversity were not integrated into the main text. Why?

The present order of paragraphs do not appear to follow a logical order. As well as figures that mix up different biological question in one figure (for example, Extended Figure 3a should go with Figure 2a and b; Extended Figure 3a should go with Figure 2 c and d). Some reshuffling would be helpful for the general understanding of the paper.

Minor points :

Line 4 suggest that cluster roots are widespread in the plant kingdom. Is that so? Next line, is the only interest of these plant structures to improve fertilizer efficiency -or also better cope without fertilizer?

The maximum number of sequenc gaps …: reword these 3 lines.
RNAseq data from 10 organs …: list them
End of page 3: why " " at "wild" ?
Top of page 4: what means retraced?
Top of page 5: some species developed cluster roots … list the species
Top of page 5: this resulted … rather this was accompanied by ?
Replace : Deprived of cluster roots by devoid of cluster roots
End of page 5: the fact that gene expression is different in Graecus and Amiga does not necessarily prove that these genes are causal of the cluster too phenotype, they are just associated with the phenotype.
Top p6 : wording not clear
Table 1: annotated non-cpoding proteins genes … reword.
Figure 1d: what does this show?
Figure 2 a&c: too small. 2b: non-domesticated is incorrect. A landrace is domesticated. In legend; "Non domesticated /landrace varieties": change varieties to accessions.
Supplementary Figure 5: TOP & BOTTOM change to a & b

Reviewer #2 (Remarks to the Author):

This paper reports the genome sequence and annotation of white lupin (Lupinus albus).
Some findings that I found interesting:
o Identification of the centromeric repeats (computationally and with immunostaining).
o The finding of three clades, both by sequence and by domestication history: winter varieties, spring varieties, and landraces.
o Inference of an ancestral lupin karyotype of 9 chromosomes, followed by a triplication.
o Loss of mycorrhizal-specific genes
o Identification of candidate genes and regions for control of phosphate acquisition.

The assembly and annotations are available as described.

The supplementary methods text looks well organized and thorough.

Suggestions:

Regarding the Ancestral Legume Karyotypes (ALK): this is an interesting result, and one that I find plausible. The conclusion (16 ancestral chromosomes) is similar to the model of a minimum of 19 proto-chromosomes reported in Kreplak et al. (2019; https://doi.org/10.1038/s41588-019-0480-1). The inference of 9 chromosomes early in the papilionoid radiation is similar to the conclusion of Ren et al. (2019; DOI: 10.1111/nph.15770) - who also hypothesize that 9 chromosomes represent an intermediate state, relative to an earlier complement of 14 chromosomes in the Papilionoideae. It would seem appropriate to cite both of these recent works.

Also regarding the ALK: it would be helpful to other researchers (and reasonable for the purposes of reproducibility) to provide data about the composition of the AKL blocks. A summary of chromosomes involved is given in ST4, but this is not useful for the purpose of identifying particular blocks in other species. I imagine that block composition could be given in terms of genes contained within each block, from some reference species (say, Medicago, lupin, or Phaseolus).

Minor (wording suggestions, for grammatical consistency and clarity):
"contain a single sequence gap illustrating" -->
"contain only a single sequence gap, illustrating"

"ten different organs widely covering gene expression" -->
"ten different organs, widely covering gene expression"

"gathers a genome browser" --> "provides a genome browser"

"questions whether these developmental structures appeared independently" -->
"raises the question of whether these developmental structures appeared independently"

"Since phosphate is a limited resource, it could represent an"
"Since phosphate is a limited resource, improved phosphate acquisition could represent an"

Reviewer #3 (Remarks to the Author):

The manuscript "Genome sequence of the cluster root forming white lupin" by Hufnagel et al. describes genome and transcriptome sequencing of white lupins, unravels its complicated paleohistory, and identifies a potential regulator of cluster root development. The work that is presented is huge, and the detailed analysis appears sound. Fig 2C alone, showing the complicated paleohistory within the legume family is amazing and informative, demonstrating relevance of this work beyond white lupin. The paper also offers a computational toolbox, the WL Genome portal, which includes a genome browser and several user-friendly tools that will be of interest to the white lupin community.
White lupin has become a model plant for the study of plant adaptations to phosphorus deficiency, due to its ability to form specialized root structures called cluster roots. Such cluster roots are found in a number of plant families, and an interesting question is how their development is regulated. The current manuscript shows a correlation of expression that points to LaPUCHI-1 as a potential regulator of cluster root development, though no causation is shown; this will be an interesting candidate gene to analyze in the future.

While I am impressed with the content, the presentation of this paper needs work. The manuscript lacks some important information: there is almost no introduction, no headers and subheaders, and some approaches are not sufficiently described (e.g. FISH is only mentioned in a figure legend, but not in the text). On the other hand, 46 supplementary figures and a 50 page "supplementary note" that reads like a thesis make this paper an overwhelming and redundant read.
I suggest to incorporate some of the "supplementary note" into the main paper, while reducing the supplements to additional information, avoiding redundancy.

Reviewers' comments:

Reviewer #1 (Remarks to the Author):

Hufnagel et al. “Genome sequence of the cluster root forming white lupin” was published in BioRXiv (July 2019) and reports the first version of the white lupin genome. White lupin is a seed protein-rich crop that has developed an original adaptation to low soil phosphorus. Its genome sequence will help deciphering these traits in this species. The manuscript provides a high quality genome sequence for variety AMIGA, as well as two genome sequences of white lupin germplasm accessions. The authors analysed the structure, evolution, diversity and expression of the white lupin genome. However, the extremely condensed and sometimes over simplified form of the manuscript does not do justice to the work that has been done.

The manuscript was originally formatted as a « Letter » and has now been reformatted with extensive rewriting. We hope that this new version will do justice to our work.

In the abstract and in the text, the use of the term domestication is mostly inadequate. Comparing Graecus and Amiga gives information about the diversity between these two lines. To investigate the impact of domestication, the authors should have included much more wild and cultivated genotypes in their analysis.

We agree with this comment and have now removed several occurrences of the word domestication and only use it with conditional wording.

Similarly, the claim that LaPUCHI-1 was identified as a potential regulator of cluster root establishment is a bit excessive. Authors identified a set of genes (including TFs) regulated in the root region developing cluster roots but to qualify one of these genes as regulator would require more in depth analysis and validation.

This comment is consistent with the comment of Reviewer #3: “The current manuscript shows a correlation of expression that points to LaPUCHI-1 as a potential regulator of cluster root development, though no causation is shown; this will be an interesting candidate gene to analyse in the future.” We have therefore tried to provide a common answer to both comments by removing mention to *LaPUCHI-1* from the abstract and toning down our conclusions about the role of this gene in cluster root development.

Overall, it appears that some parts of the text would deserve more emphasis.

This comment has been addressed through major re-writing of the text.

In the paragraph about genome assembly, some background should be given on the advantages of the technologies used.
In the new version of the text, we provide more information regarding the data produced (read size). Today, long read technologies are widely used in plant

genomics. The benefit of these data is an established knowledge and we believe that we don't need to argue and compare with short reads based strategies in this work. We prefer to keep space in order to focus our article on the exploitation of our high-quality genome.

During the annotation process, only the M. truncatula proteome was used. Using the proteomes of other related species such as Glycine max and Arachis duranensis would probably have contributed to improve the annotation.

This is incorrect; the annotation process used 3 databases, including Medicago proteome but also Swiss-Prot and a subset of Uniprot. Since this was not clear enough, we amended the text to read "Three protein databases (Swiss-Prot, a plant subset of Uniprot proteins and the proteome of *Medicago truncatula*) were aligned to contribute to translated regions detection."

The annotation of repetitive DNA identified 60% of repeats but 25% of these TEs were uncharacterised. Can the authors provide some information about these uncharacterised repeats?

In fact, only approximately 12% of TEs were uncharacterised not 25% (Fig 1a; Suppl. Note Table 5). Any TE lacking a protein domain that does not enable a proper TE classification to main TE classes based on BLAST search against the REXdb from RepeatExplorer were classified as an uncharacterized TE. REXdb database integrates all main repeat databases and is the most used for plant repeat annotation (Neumann et al. 2019; DOI: 10.1186/s13100-018-0144-1). Thus, most likely uncharacterized TEs are either degenerated or lack important TE domains so we do not expand our description of these elements in the text.

The paragraph about the location of the different repeats and the FISH data should be better integrated.
This subsection has now been fully re-written and gives a better description of the cytogenetic work.

The paloegenomic analysis of the evolution leading to the white lupin genome looks powerful. But more background on the different approaches already published, the rationale for the choice of the approach used, and its comparison with previous reports would be useful to the reader.

The method used to reconstruct the ancestral genomes based on conserved genes identified between the investigated genomes is described in previous articles mentioned in the method section notably Pont et al., 2019 (Paleogenomics: reconstruction of plant evolutionary trajectories from modern and ancient DNA. Genome Biol. 20, 29), and Salse, 2016 (Ancestors of modern plant crops. Curr Opin Plant Biol. 30:134-42), referenced in the Supplementary Note 5.1. In these articles, the approach, parameters and tools used to identify (1) conserved gene between pairs of species, (2) syntenic blocks of conserved genes between pairs of species, and (3) conserved ancestral regions (CARs) of syntenic

blocks conserved between all the investigated genomes, are described. In order to answer the reviewer's demand, we have developed the Supplementary Note 4.1 of the method used to reconstruct the ancestral legume genomes. Also, the subsection entitled "White lupin genome evolution" now gives a better description of the approach and its conclusions.

The de novo assembly of three genomes of white lupin using the latest long-read technologies is a significant output of this study. The white lupin diversity study should better take advantage of these genome sequences that define a white lupin pangenome. What is the impact of structural variation on the genome? what are the regions impacted and their link with TE rich regions? A more in-depth analysis of these data would probably bring interesting results. Extended data Figure 3a should be included in the main text.

The new version of the text in the subsection entitled "White lupin diversity and genomic structural variations" now partly answers these questions but an in-depth analysis will constitute follow-up studies and goes beyond the scope of the present manuscript. However, we analysed the genes affected by structural variations and used this analysis to create Supp. Fig 7. Interestingly, 671 genes are affected both in GREACUS and P27174. Extended data have been largely remodelled and Fig 3a is now part of main Figure 2 as suggested.

In the diversity study, some background on white lupin region of origin (why pick up lines from Greece, Ethiopia, but also France, Germany, Ukraine), domestication, breeding, and area of production would be really useful to discuss more fully these results. Also, the description of accessions should be revised. Do the 14 accessions really cover a wide range of diversity? 8 accessions come from France…

Varieties were chosen because they represent largely used commercial varieties of White Lupin produced in the past 20 years and may therefore be useful for breeding programs using these varieties. We believe that our reference genome will open the way to re-sequencing work that may encompass larger pools of accessions and provide more information related to their origin and specificities. However, this will require that the community provides a better characterization of the available stocks present in the seed banks worldwide.

In Supplemental Table 7, Amiga and Feodora appear to come from Chile and Germany but at another place in the manuscript (Suppl. 3.2.1) it is indicated that Amiga and Feodora come from a French breeding program.

This is totally consistent, these accessions come from Chile and Germany respectively and were both used in a French breeding program.

In the same paragraph (suppl 3.2.1), winter and spring accessions' attributes are mixed up. In different places in the text, the term variety is used irrelevantly, because a wild accession is not a variety.

In the new version of the text, we corrected errors regarding the attribute of different WL accessions and we have been very careful to use the word “accession” where we used incorrectly the word “variety”.

It is surprising that the data on seed trait diversity were not integrated into the main text. Why?

It was only due to space constraint due to the Letter format of a previous submission. In the new version of the text, we have now integrated the results on seed analysis in a subsection called “Seed quality”

The present order of paragraphs do not appear to follow a logical order. As well as figures that mix up different biological question in one figure (for example, Extended Figure 3a should go with Figure 2a and b; Extended Figure 3a should go with Figure 2 c and d). Some reshuffling would be helpful for the general understanding of the paper.

This has been done, we hope that the new logical order of the text will be helpful to the general understanding of the paper.

Minor points :
Line 4 suggest that cluster roots are widespread in the plant kingdom. Is that so?
Species from 10 botanical families can form cluster roots as now stated in the Discussion, this is larger than the ability to form nitrogen-fixing nodules but still not that widely spread.

Next line, is the only interest of these plant structures to improve fertilizer efficiency -or also better cope without fertilizer?
These structures improve phosphate acquisition efficiency, so they can do both.

The maximum number of sequenc gaps …: reword these 3 lines.
Done.

RNAseq data from 10 organs …: list them
Done. (entire root system in +P and – P condition, lateral roots, primary roots, cluster roots, nodulated root system, leaves, flowers, pods and seeds).

End of page 3: why “ ” at “wild” ?
The quotes have been removed.

Top of page 4: what means retraced?
We believe it is a nice way to say that we “performed” the paleohistory analysis.

Top of page 5: some species developed cluster roots … list the species
The list of species forming cluster root is fairly long and wouldn’t add much to the manuscript. The cited reference from Skene 2000 provides many complementary

information.

Top of page 5: this resulted … rather this was accompanied by ?
Done.

Replace : Deprived of cluster roots by devoid of cluster roots
Done.

End of page 5: the fact that gene expression is different in Graecus and Amiga does not necessarily prove that these genes are causal of the cluster too phenotype, they are just associated with the phenotype.
Agreed.

Table 1: annotated non-cpoding proteins genes … reword.

Changed to "Annotated non-protein coding genes »

Figure 1d: what does this show?
This figure shows which reads from the ChIPseq analysis map to the genome, this identifies sites for CENH3 interactions with chromosomes that therefore correspond to centromeric regions.

Figure 2 a&c: too small. 2b: non-domesticated is incorrect. A landrace is domesticated. In legend; "Non domesticated /landrace varieties": change varieties to accessions.
Changes were made accordingly (Figure 2 panels are now bigger, Non-domesticated was amended to "Wild and landraces").

Supplementary Figure 5: TOP & BOTTOM change to a & b
This figure has now been incorporated into Figure 3 panel b.

Reviewer #2 (Remarks to the Author):

This paper reports the genome sequence and annotation of white lupin (Lupinus albus).
Some findings that I found interesting:
o Identification of the centromeric repeats (computationally and with immunostaining).
o The finding of three clades, both by sequence and by domestication history: winter varieties, spring varieties, and landraces.
o Inference of an ancestral lupin karyotype of 9 chromosomes, followed by a triplication.
o Loss of mycorrhizal-specific genes
o Identification of candidate genes and regions for control of phosphate acquisition.

The assembly and annotations are available as described.

The supplementary methods text looks well organized and thorough.

Suggestions:

Regarding the Ancestral Legume Karyotypes (ALK): this is an interesting result, and one that I find plausible. The conclusion (16 ancestral chromosomes) is similar to the model of a minimum of 19 proto-chromosomes reported in Kreplak et al. (2019; https://doi.org/10.1038/s41588-019-0480-1). The inference of 9 chromosomes early in the papilionoid radiation is similar to the conclusion of Ren et al. (2019; DOI: 10.1111/nph.15770) - who also hypothesize that 9 chromosomes represent an intermediate state, relative to an earlier complement of 14 chromosomes in the Papilionoideae. It would seem appropriate to cite both of these recent works.

Agreed, we have now included the Kreplak et al. (2019) reference in the Supplementary Note 5.1 where Ren et al. (2019) was already mentioned.

Also regarding the ALK: it would be helpful to other researchers (and reasonable for the purposes of reproducibility) to provide data about the composition of the AKL blocks. A summary of chromosomes involved is given in ST4, but this is not useful for the purpose of identifying particular blocks in other species. I imagine that block composition could be given in terms of genes contained within each block, from some reference species (say, Medicago, lupin, or Phaseolus).

The Supplementary Table 4 (ST4) allows any reader to identify conserved chromosomal regions among the investigated species at the basis of the 16 inferred ancestral chromosomes. In order to give access not only the conserved blocks (ST4) but to all the catalogue of conserved genes (as requested by the reviewer) identified between the legume genomes, we made the comparative genomics data are going to be made publicly available at https://urgi.versailles.inra.fr/synteny/legumes at the time of acceptance of the manuscript (referenced in the Supplementary Note 4.1 section).

Minor (wording suggestions, for grammatical consistency and clarity):
"contain a single sequence gap illustrating" -->
"contain only a single sequence gap, illustrating"
Done.

"ten different organs widely covering gene expression" -->
"ten different organs, widely covering gene expression"
Done.

"gathers a genome browser" --> "provides a genome browser"
Done.

"questions whether these developmental structures appeared independently" -->
"raises the question of whether these developmental structures appeared independently"
Done.

"Since phosphate is a limited resource, it could represent an"
"Since phosphate is a limited resource, improved phosphate acquisition could represent an"
Done.

Reviewer #3 (Remarks to the Author):

The manuscript “Genome sequence of the cluster root forming white lupin” by Hufnagel et al. describes genome and transcriptome sequencing of white lupins, unravels its complicated paleohistory, and identifies a potential regulator of cluster root development. The work that is presented is huge, and the detailed analysis appears sound. Fig 2C alone, showing the complicated paleohistory within the legume family is amazing and informative, demonstrating relevance of this work beyond white lupin. The paper also offers a computational toolbox, the WL Genome portal, which includes a genome browser and several user-friendly tools that will be of interest to the white lupin community.

White lupin has become a model plant for the study of plant adaptations to phosphorus deficiency, due to its ability to form specialized root structures called cluster roots. Such cluster roots are found in a number of plant families, and an interesting question is how their development is regulated. The current manuscript shows a correlation of expression that points to LaPUCHI-1 as a potential regulator of cluster root development, though no causation is shown; this will be an interesting candidate gene to analyze in the future.

While I am impressed with the content, the presentation of this paper needs work. The manuscript lacks some important information: there is almost no introduction, no headers and subheaders, and some approaches are not sufficiently described (e.g. FISH is only mentioned in a figure legend, but not in the text). On the other hand, 46 supplementary figures and a 50 page “supplementary note” that reads like a thesis make this paper an overwhelming and redundant read.

I suggest to incorporate some of the “supplementary note” into the main paper, while reducing the supplements to additional information, avoiding redundancy.

We fully appreciate the very positive feedback on our extensive genomic study. We provide here a major revision of the text that should answer all comments raised by Reviewer #3. The new text also addresses all comments made in the attached PDF, except the following.

“and some species developed cluster roots ca. 2.5My ago.” some species? Explain does this imply that different species develop cluster roots independently at the same time?
This question remains unanswered and we discuss this point in the new version of the Discussion. Cluster roots may have been developed independently by

various species (as far as we understand), whether it is at the same time or not is not known. We believe that our work will open the way to identification of molecular regulators of cluster root formation and therefore help towards this answering these questions.

Reviewers' comments:

Reviewer #1 (Remarks to the Author):

The new version of the manuscript by Hufnagel et al., entitled "High-quality genome sequence of white lupin provides insight into soil exploration and seed quality" has been substantially improved. Some minor points still need to be addressed:
In the abstract, line 39-40 modern accessions display increased soil exploration … > which accessions ? increased relative to which accessions ? it appears from line 331-344 that only Amiga and Graecus were compared; this sentence should be changed as well as line 331 (no vision of domestication by comparing only 2 accessions). Similarly, line 350, the evidence of the impact of domestication on seed quality is weak because too few wild accessions used > Amiga seeds were associated with the disappearance of …
Line 56: reword
Line 67: according to Plant and Soil (2005) 274:101–125, cucurbita pepo also displays cluster roots …
Line 24: in silico annotation rather than distribution
L192-207: condense the message
Line 218-232: reword; no clear
Line 457: publicly available rather than already?
Line 459 : Vicia faba in italics
Line 521-22: labelling
Line 541: Immunoprecipitated DNA samples, and …
Line 577: new paragraph?
Line 593-598: reword
Line 613: …using Assemblytics …
Line 969: wild instead of non-domesticated?
Line 982-983: reword
Line 994: delet Dot-plot based deconvolution?
Line 1013: not clear. When only 1 dot, is it an intersection? Or only section specific?

Reviewer #2 (Remarks to the Author):

This revision is substantial improvement over the previous version - mostly through changes made in response to recommendations to the other reviewers. This revision adequately addresses my suggestions from the previous version. I have no further suggestions.

Reviewer #3 (Remarks to the Author):

The manuscript "Genome sequence of the cluster root forming white lupin" by Hufnagel et al. has been revised and significantly extended. My previous review concerns have been addressed, and the structure of the paper has improved. Some of the result sections will benefit from shortening to tell a more concise story, especially the sections "Repetitive elements and structure of centromeric regions" and "White lupin diversity and genomic structural variations". At the same time, these sections should be at least briefly interpreted in the discussion. Please find specific points directly in the attached pdf.

Responses to reviewers are indicated in bold.

Editorial comment about data availability:

In addition, you state “All data have been deposited to the NCBI public repository, waiting for final acceptance”. Since data submission and the response of NCBI can be a lengthy process, and we strongly suggest that you begin to release the sequences data to public in advance of potential publication. Please include a statement about data availability in your point-by-point letter accompanying your revisions. Please see below for more detailed information about data requirements and policy. If you are unable to make your data publicly available for exceptional reasons, please get in touch with me now to discuss this further.
In response to this comment, we want to attract your attention on 2 points:
-Firstly, all data used in the manuscript are already publicly available on the White Lupin portal (www.whitelupin.fr) since July 19th (publication as a preprint).
-Secondly, we started the submission process at the NCBI repository on October 3rd and we are very close to final acceptance (Biosample accession number SAMN12906581). The release date has been set to December 31st or the date of publication of the present article, whichever comes first.
We are open to discussion about this point but we believe that final acceptance by NCBI should not delay further steps of manuscript handling on your side since anyway the data is already publicly available.

Reviewer #1 (Remarks to the Author):

The new version of the manuscript by Hufnagel et al., entitled “High-quality genome sequence of white lupin provides insight into soil exploration and seed quality” has been substantially improved.
Some minor points still need to be addressed:
In the abstract, line 39-40 modern accessions display increased soil exploration … > which accessions ? increased relative to which accessions ? it appears from line 331-344 that only Amiga and Graecus were compared; this sentence should be changed as well as line 331 (no vision of domestication by comparing only 2 accessions).
We agree that 2 accessions are not enough to conclude about domestication. The text was amended accordingly in the abstract.

Similarly, line 350, the evidence of the impact of domestication on seed quality is weak because too few wild accessions used > Amiga seeds were associated with the disappearance of …
The text was amended to remove the mention to “domestication”.

Line 56: reword
We are not sure about the issue here, the text now reads : “This crop is recognized as a traditional food due to its very high protein content…”.

Line 67: according to Plant and Soil (2005) 274:101–125, cucurbita pepo also displays cluster roots …
The text now reads : “ White Lupin is one of the few crops…”

Line 124: in silico annotation rather than distribution

Done.

L192-207: condense the message.
This paragraph was condensed (also according to Reviewer #3).

Line 218-232: reword; no clear
The entire paragraph was simplified by rephrasing part of it and removing another part (also according to Reviewer #3).

Line 457: publicly available rather than already?
Done.

Line 459 : Vicia faba in italics
Done.

Line 521-22: labelling
Done.

Line 541: Immunoprecipitated DNA samples, and …
Done.

Line 577: new paragraph?
Done.

Line 593-598: reword
Done.

Line 613: …using Assemblytics …
Done.

Line 969: wild instead of non-domesticated?
Done.

Line 982-983: reword
Done.

Line 994: delet Dot-plot based deconvolution?
Done.

Line 1013: not clear. When only 1 dot, is it an intersection? Or only section specific?
We amended the text to better explain the fact that dots represent sample parts displayed by groups of up-regulated gees.

Reviewer #2 (Remarks to the Author):

This revision is substantial improvement over the previous version - mostly through changes made in response to recommendations to the other reviewers. This revision adequately addresses my suggestions from the previous version. I have no further suggestions.

Thank you !

Reviewer #3 (Remarks to the Author):

The manuscript “Genome sequence of the cluster root forming white lupin” by Hufnagel et al. has been revised and significantly extended. My previous review concerns have been addressed, and the structure of the paper has improved. Some of the result sections will benefit from shortening to tell a more concise story, especially the sections “Repetitive elements and structure of centromeric regions” and “White lupin diversity and genomic structural variations”. At the same time, these sections should be at least briefly interpreted in the discussion. Please find specific points directly in the attached pdf.
Comments were addressed in the response to Reviewer #1. Specific comments in the PDF are listed below (with line number).

Line 76
start a new paragraph here
Done.

Line 93
208x? I don't understand, coverage?
Yes, it is the coverage, we added « a depth of 208x ».

Line 108
"integrated", what does this mean? Where they mapped to the genome?
Yes, we changed it to “mapped”.
Line 120
This section is interesting, but a bit tedious to read; perhaps some details can be moved to supplements? I appreciate the main information, but don't believe that the typical reader needs to know that much detail
This section was simplified by removing text and light rewording.

Line 150
indication?
That was a typo. Modified to « indication ».

Line 210
used a subset for what?
The sentence was amended to « We used a subset of 46,783 high-quality genomic-random distributed SNPs.”

Line 309
rephrase, how about "a zone of CR initiation"?
This now reads “where CR initiation occurs”.

Line 311
where is this going; why did you identify all those homologues? I understand that one needs to be careful with any interpretations, but there is still a story to tell
We removed our interpretation about PUCHI being a potential regulator of cluster root development in the previous round of reviews.

Line 380
present? how about "results from" or "influenced by"?
We used « noticeable ».

Line 1016
I don't understand set size, the total number of genes for each comparison? Does this mean "the total number of up-regulated genes" for each developmental stage?
We amended the text accordingly (see response to Reviwer #1).

End of introduction
I'm unsure about the last paragraph of the introduction. I learned that the end of the introduction should state research questions and objectives of the study. But I see frequently a summary of results instead, as has been done here; perhaps an editor can advise

Note from the Editor : According to Nature style, we ask authors to give a brief summary of the main conclusion of the study and the description of the results should be written in present tense.

We modified the text to be at the present tense (marked in red).

REVIEWERS' COMMENTS:

None.
